# Supplementary material for: Active trachoma among children aged 1–9 years in Ethiopia: A meta-analysis from 2019 to 2024
Source: PLoS One. 2025 May 9;20(5):e0323601. doi: 10.1371/journal.pone.0323601 (PMC12063883; doi:10.1371/journal.pone.0323601)
Supplement: S3 File — (DOCX) [file pone.0323601.s003.docx]

| S.N | study | Included/excluded | Reason for exclusion |
| --- | --- | --- | --- |
| 1 | Genet A, Dagnew Z, Melkie G, Keleb A, Motbainor A, Mebrat A, et al. Prevalence of active trachoma and its associated factors among 1–9 years of age children from model and non-model kebeles in Dangila district, northwest Ethiopia. Plos one. 2022;17(6):e0268441. | Included |  |
| 2 | Asmare ZA, Seifu BL, Mare KU, Asgedom YS, Kase BF, Shibeshi AH, et al. Prevalence and associated factors of active trachoma among 1–9 years of age children in Andabet district, northwest Ethiopia, 2023: A multi-level mixed-effect analysis. PLOS Neglected Tropical Diseases. 2023;17(8):e0011573. | Included |  |
| 3 | Altaseb T, Lingerew M, Adane M. Prevalence of trachomatous inflammation-follicular and associated factors among children aged 1-9 years in northeastern Ethiopia. BMC pediatrics. 2024;24(1):128. | Included |  |
| 4 | Melkie G, Azage M, Gedamu G. Prevalence and associated factors of active trachoma among children aged 1-9 years old in mass drug administration graduated and non-graduated districts in Northwest Amhara region, Ethiopia: A comparative cross-sectional study. PLoS One. 2020;15(12):e0243863. | Included |  |
| 5 | Shimelash A, Alemayehu M, Dagne H, Mihiretie G, Lamore Y, Tegegne E, et al. Prevalence of active trachoma and associated factors among school age children in Debre Tabor Town, Northwest Ethiopia, 2019: a community based cross-sectional study. Italian Journal of Pediatrics. 2022;48(1):61 | Included |  |
| 6 | Mekonnen J, Kassim J, Ahmed M, Gebeyehu N. Prevalence of active trachoma and associated factors among children 1–9 years old at Arsi Negele Town, West Arsi Zone, Oromia Regional State, Southern Ethiopia. Plos one. 2022;17(10):e0273808. | Included |  |
| 7 | Prevalence of active trachoma and associated factors among school age children in Debre Tabor Town, Northwest Ethiopia, 2019: a community based cross-sectional study | Excluded | Dupilcate |
| 8 | Kassim, Kemal, et al. "Prevalence of active trachoma and associated risk factors among children of the pastoralist population in Madda Walabu rural district, Southeast Ethiopia: a community-based cross-sectional study." *BMC infectious diseases* 19 (2019): 1-7. | Excluded | Duplicate |
| 9 | ABDULWHAB NA, ELHAG WI. Molecular Detection of Chlamydia Trachomatis Associated with Ocular Infection among Children in Gadarif State-Sudan. | Excluded | Outcome of interest not reported and the study done outside Ethiopia |
| 10 | Olamiju F, Isiyaku S, Olobio N, Mogaji H, Achu I, Muhammad N, Boyd S, Bakhtiari A, Ebenezer A, Jimenez C, Solomon AW. Prevalence of Trachoma following implementation of the SAFE strategy in three local government areas of Taraba State, North Eastern Nigeria. Ophthalmic epidemiology. 2023 Nov 2;30(6):619-27. | Excluded | Outcome of interest not reported and the study done outside Ethiopia |
| 11 | Chen X, Munoz B, Mkocha H, Wolle MA, K. West S. Children as messengers of health knowledge? Impact of health promotion and water infrastructure in schools on facial cleanliness and trachoma in the community. PLoS neglected tropical diseases. 2021 Feb 1;15(2):e0009119. | Excluded | Outcome of interest not reported and the study done outside Ethiopia |
| 12 | Mekonnen, Jemal, Jeylan Kassim, Muluneh Ahmed, and Negeso Gebeyehu. "Prevalence of active Trachoma and associated factors among children 1–9 years old at Arsi Negele town, West Arsi zone, Oromia regional state, Southern Ethiopia." *Plos one* 17, no. 10 (2022): e0273808. | Excluded | Duplicate |
| 13 | Solomon AW, Burton MJ, Gower EW, Harding-Esch EM, Oldenburg CE, Taylor HR, Traoré L. Trachoma (Primer). Nature Reviews: Disease Primers. 2022;8(1). | Excluded | Wrong study design and Outcome of interest not reported |
| 14 | Tuke D, Etu E, Shalemo E. Active trachoma prevalence and related variables among children in a pastoralist community in southern Ethiopia in 2021: a community-based cross-sectional study. The American Journal of Tropical Medicine and Hygiene. 2023;108(2):252. | Included |  |
| 15 | Astale T, Ebert CD, Nute AW, Zerihun M, Gessese D, Melak B, Sata E, Ayele Z, Ayenew G, Callahan EK, Haile M. The population-based prevalence of trachomatous scarring in a trachoma hyperendemic setting: results from 152 impact surveys in Amhara, Ethiopia. BMC ophthalmology. 2021 May 13;21(1):213. | Excluded | Outcome of interest not reported |
| 16 | Kassim, Kemal, et al. "Prevalence of active trachoma and associated risk factors among children of the pastoralist population in Madda Walabu rural district, Southeast Ethiopia: a community-based cross-sectional study." *BMC infectious diseases* 19 (2019): 1-7. | Excluded | Duplicate |
| 17 | Butcher R, Tagabasoe J, Manemaka J, Bong A, Garae M, Daniel L, Roberts C, Handley BL, Hu VH, Harding-Esch EM, Bakhtiari A. Conjunctival scarring, corneal pannus, and herbert’s Pits in adolescent children in trachoma-endemic populations of the Solomon Islands and Vanuatu. Clinical Infectious Diseases. 2021 Nov 1;73(9):e2773-80. | Excluded | study done outside Ethiopia |
| 18 | Tanywe AC, Matchawe C, Fernandez R, Lapkin S. Perceptions and practices of community members relating to trachoma in Africa: a qualitative systematic review protocol. JBI Evidence Synthesis. 2019 Nov 1;17(11):2350-6. | Excluded | Wrong study design, Outcome of interest not reported and the study done outside Ethiopia |
| 19 | Macleod CK, Bailey RL, Dejene M, Shafi O, Kebede B, Negussu N, Mpyet C, Olobio N, Alada J, Abdala M, Willis R. Estimating the intracluster correlation coefficient for the clinical sign “trachomatous inflammation—follicular” in population-based trachoma prevalence surveys: results from a meta-regression analysis of 261 standardized preintervention surveys carried out in Ethiopia, Mozambique, and Nigeria. American journal of epidemiology. 2020 Jan 31;189(1):68-76. | Excluded | Outcome of interest not reported |
| 20 | Ramadhani AM, Derrick T, Macleod D, Massae P, Mafuru E, Malisa A, Mbuya K, Roberts CH, Makupa W, Mtuy T, Bailey RL. Progression of scarring trachoma in Tanzanian children: A four-year cohort study. PLoS neglected tropical diseases. 2019 Aug 14;13(8):e0007638. | Excluded | Wrong study design, Outcome of interest not reported and the study done outside Ethiopia |
| 21 | Prevalence of active trachoma and associated factors among school age children in Debre Tabor Town, Northwest Ethiopia, 2019: a community based cross-sectional study | Excluded | Duplicate |
| 22 | Altaseb T, Lingerew M, Adane M. Prevalence of trachomatous inflammation-follicular and associated factors among children aged 1-9 years in northeastern Ethiopia. BMC pediatrics. 2024 Feb 19;24(1):128. | Excluded | duplicate |
| 23 | Asmare, Z. A., Seifu, B. L., Mare, K. U., Asgedom, Y. S., Kase, B. F., Shibeshi, A. H., ... & Asebe, H. A. (2023). Prevalence and associated factors of active trachoma among 1–9 years of age children in Andabet district, northwest Ethiopia, 2023: A multi-level mixed-effect analysis. *PLOS Neglected Tropical Diseases*, *17*(8), e0011573. | Excluded | duplicate |
| 24 | Tedijanto C, Solomon AW, Martin DL, Nash SD, Keenan JD, Lietman TM, Lammie PJ, Aiemjoy K, Amza A, Aragie S, Arzika AM. Monitoring transmission intensity of trachoma with serology. Nature communications. 2023 Jun 5;14(1):3269. | Excluded | Outcome of interest not reported |
| 25 | Kassaw MW, Tegegne KD, Tegegne KM, Ahmed M. Stakeholder’s perception on the slow progress towards trachoma elimination and suggested recommendations for future intervention: An interpretive qualitative study in Bugna District, Northeast Ethiopia. | Excluded | Outcome of interest not reported |
| 26 | Kassaw, M. W., Abebe, A. M., Tegegne, K. D., Getu, M. A., & Bihonegn, W. T. (2020). Prevalence and associations of active trachoma among rural preschool children in Wadla district, northern Ethiopia. *BMC ophthalmology*, *20*, 1-10. | Excluded | Duplicate |
| 27 | Amza A, Kadri B, Nassirou B, Cotter SY, Stoller NE, West SK, Bailey RL, Porco TC, Keenan JD, Lietman TM, Oldenburg CE. Community-level association between clinical trachoma and ocular chlamydia infection after MASS azithromycin distribution in a mesoendemic region of Niger. Ophthalmic epidemiology. 2019 Jul 4;26(4):231-7. | Excluded | Outcome of interest not reported and the study done outside Ethiopia |
| 28 | Alambo, Melese Menta, et al. "Prevalence of active trachoma and associated factors in Areka Town, south Ethiopia, 2018." *Interdisciplinary Perspectives on Infectious Diseases* 2020.1 (2020): 8635191. | Excluded | Duplicate |
| 29 | Alambo, Melese Menta, et al. "Prevalence of active trachoma and associated factors in Areka Town, south Ethiopia, 2018." *Interdisciplinary Perspectives on Infectious Diseases* 2020.1 (2020): 8635191. | Excluded | Duplicate |
| 30 | Asfaw, Mekuria, et al. "Towards the trachoma elimination target in the Southern region of Ethiopia: How well is the SAFE strategy being implemented?." *The Journal of Infection in Developing Countries* 14.06.1 (2020): 3S-9S. | Excluded | Duplicate |
| 31 | Kassim, Kemal, et al. "Prevalence of active trachoma and associated risk factors among children of the pastoralist population in Madda Walabu rural district, Southeast Ethiopia: a community-based cross-sectional study." *BMC infectious diseases* 19 (2019): 1-7. | Excluded | Duplicate |
| 31 | West SK, Nanji AA, Mkocha H, Munoz B, Gaydos C, Quinn TC. Evidence for contamination with C. trachomatis in the household environment of children with active Trachoma: A cross-sectional study in Kongwa, Tanzania. PLoS neglected tropical diseases. 2019 Dec 23;13(12):e0007834. | Excluded | Outcome of interest not reported and the study done outside Ethiopia |
| 32 | Dyer CE, Kalua K, Chisambi AB, Wand H, McManus H, Liu B, Kaldor JM, Vaz Nery S. Water, Sanitation, and Hygiene (WASH) Factors Influencing the Effectiveness of Mass Drug Administration to Eliminate Trachoma as a Public Health Problem in Malawi. Ophthalmic Epidemiology. 2024 Mar 3;31(2):127-33. | Excluded | Outcome of interest not reported and the study done outside Ethiopia |
| 33 | Mekonnen, Jemal, Jeylan Kassim, Muluneh Ahmed, and Negeso Gebeyehu. "Prevalence of active Trachoma and associated factors among children 1–9 years old at Arsi Negele town, West Arsi zone, Oromia regional state, Southern Ethiopia." *Plos one* 17, no. 10 (2022): e0273808. | Excluded | Duplicate |
| 34 | Lietman TM, Oldenburg CE, Keenan JD. Trachoma: time to talk eradication. Ophthalmology. 2020 Jan 1;127(1):11-3. | Excluded | Outcome of interest not reported |
| 35 | Lietman TM, Ayele B, Gebre T, Zerihun M, Tadesse Z, Emerson PM, Nash SD, Porco TC, Keenan JD, Oldenburg CE. Frequency of mass azithromycin distribution for ocular chlamydia in a trachoma endemic region of Ethiopia: a cluster randomized trial. American journal of ophthalmology. 2020 Jun 1;214:143-50. | Excluded | Outcome of interest not reported and wrong study design |
| 36 | Prevalence of active trachoma and associated factors among school age children in Debre Tabor Town, Northwest Ethiopia, 2019: a community based cross-sectional study | Exluded | Duplicate |
| 37 | Altaseb T, Lingerew M, Adane M. Prevalence of trachomatous inflammation-follicular and associated factors among children aged 1-9 years in northeastern Ethiopia. BMC pediatrics. 2024 Feb 19;24(1):128. | Excluded | Duplicate |
| 38 | Getachew, Dawit, et al. "High prevalence of active trachoma and associated factors among school-aged children in Southwest Ethiopia." PLOS Neglected Tropical Diseases 17.12 (2023): e0011846. | Excluded | Duplicate |
| 39 | Wolle MA, Muñoz BE, Naufal F, Kashaf MS, Mkocha H, West SK. Risk factors for the progression of trachomatous scarring in a cohort of women in a trachoma low endemic district in Tanzania. PLoS neglected tropical diseases. 2021 Nov 19;15(11):e0009914. | Excluded | Outcome of interest not reported and the study done outside Ethiopia |
| 40 | West SK. Milestones in the fight to eliminate trachoma. Ophthalmic and Physiological Optics. 2020 Mar;40(2):66-74. | Excluded | Outcome of interest not reported |
| 41 | Last A, Versteeg B, Shafi Abdurahman O, Robinson A, Dumessa G, Abraham Aga M, Shumi Bejiga G, Negussu N, Greenland K, Czerniewska A, Thomson N. Detecting extra-ocular Chlamydia trachomatis in a trachoma-endemic community in Ethiopia: Identifying potential routes of transmission. PLoS neglected tropical diseases. 2020 Mar 4;14(3):e0008120. | Excluded | Outcome of interest not reported |
| 42 | Bella AL, Einterz E, Huguet P, Bensaid P, Amza A, Renault D. Effectiveness and safety of azithromycin 1.5% eye drops for mass treatment of active trachoma in a highly endemic district in Cameroon. BMJ open ophthalmology. 2020 Nov 1;5(1). | Excluded | Outcome of interest not reported |
| 43 | Gower EW, Munoz B, Rajak S, Habtamu E, West SK, Merbs SL, Harding JC, Alemayehu W, Callahan EK, Emerson PM, Gebre T. Pre-operative trichiatic eyelash pattern predicts post-operative trachomatous trichiasis. PLoS neglected tropical diseases. 2019 Oct 7;13(10):e0007637. | Excluded | Outcome of interest not reported |
| 44 | Oldenburg CE, Aragie S, Amza A, Solomon AW, Brogdon J, Arnold BF, Keenan JD, Lietman TM. Can we eradicate trachoma? A survey of stakeholders. British Journal of Ophthalmology. 2021 Aug 1;105(8):1059-62. | Excluded | Outcome of interest not reported and the study done outside Ethiopia |
| 45 | Salam AS, Qayumi R, Majeed Siddiqi A, Naseem M, Mansoor M, Butcher R, Bakhtiari A, Renneker K, Willis R, Jimenez C, Dejene M. Prevalence of trachoma in 72 districts of Afghanistan in 2018− 2019: results of 35 population-based prevalence surveys. Ophthalmic Epidemiology. 2023 Nov 2;30(6):608-18. | Excluded | the study done outside Ethiopia |
| 46 | \| Bah MM, Sakho F, Goepogui A, Nieba LC, Cisse A, Courtright P, Harte AJ, Burgert-Brucker C, Jimenez C, Lama PL, Sagno M. The Prevalence of Trachomatous Trichiasis in People Aged 15 Years and Over in Six Evaluation Units of Gaoual, Labé, Dalaba and Beyla Districts, Guinea. Ophthalmic epidemiology. 2024 Nov 1;31(6):526-33. \| \| --- \| | Excluded | Outcome of interest not reported and the study done outside Ethiopia |
| 47 | Gebrie, Alemu, Animut Alebel, Abriham Zegeye, Bekele Tesfaye, and Fasil Wagnew. "Prevalence and associated factors of active trachoma among children in Ethiopia: a systematic review and meta-analysis." *BMC infectious diseases* 19 (2019): 1-12. | Excluded | Duplicate |
| 48 | Quesada-Cubo V, Damián-González DC, Prado-Velasco FG, Fernández-Santos NA, Sánchez-Tejeda G, Correa-Morales F, Domínguez-Zárate H, García-Orozco A, Saboyá-Díaz MI, Sánchez-Martín MJ. The elimination of trachoma as a public health problem in Mexico: From national health priority to national success story. Plos Neglected Tropical Diseases. 2022 Aug 29;16(8):e0010660. | Excluded | Outcome of interest not reported and the study done outside Ethiopia |
| 49 | \| Ofoegbu OO. Evaluation of trichiasis surgeons' performance in selected trachoma endemic African countries. \| \| --- \| | Excluded | Outcome of interest not reported and the study done outside Ethiopia |
| 50 | \| Abayo G, Gessesse GW, Asaminew T. Prevalence and pattern of ocular morbidity among school children in southern Ethiopia. Ethiopian Journal of Health Sciences. 2021 Jul 1;31(4). \| \| --- \| | Excluded | Duplicate |
| 51 | Shafi Abdurahman O, Last A, Macleod D, Habtamu E, Versteeg B, Dumessa G, et al. Trachoma risk factors in Oromia Region, Ethiopia. PLoS Neglected Tropical Diseases. 2023;17(11):e0011679. | Included |  |
| 52 | Glagn Abdilwohab M, Hailemariam Abebo Z. High prevalence of clinically active trachoma and its associated risk factors among preschool-aged children in arba Minch health and demographic surveillance site, southern Ethiopia. Clinical Ophthalmology. 2020:3709-18. | Included |  |
| 55 | Getachew, Dawit, et al. "High prevalence of active trachoma and associated factors among school-aged children in Southwest Ethiopia." PLOS Neglected Tropical Diseases 17.12 (2023): e0011846. | Excluded | Duplicate |
| 56 | Kedir S, Lemnuro K, Yesse M, Abdella B, Muze M, Mustefa A, et al. Prevalence and Factors Associated with Active Trachoma among Children 1-9 years of Age in the Catchment Population of Tora Primary Hospital, Silte zone, Southern Ethiopia, 2020. The Open Ophthalmology Journal. 2021;15(1). | Included |  |
| 57 | Alemayehu A, Mekonen A, Mengistu B, Mihret A, Asmare A, Bakhtiari A, et al. Prevalence of Trachoma After Three Rounds of Antibiotic Mass Drug Administration in 13 Woredas of Gambella Region, Ethiopia. Ophthalmic epidemiology. 2023:1-9. | Included |  |
| 58 | Senbete L, Adhena G. Magnitude of Active Trachoma Among Host and Refugee Children in Gambella Regional State, Ethiopia. Clinical Ophthalmology. 2024:777-89. | Included |  |
| 59 | Mengistu B, Wirtu F, Alemayehu A, Alene S, Asmare A, Backers S, et al. Prevalence of trachoma in Benishangul Gumuz Region, Ethiopia, after implementation of the SAFE strategy: Results of four population-based surveys. Ophthalmic epidemiology. 2022:1-9. | Included |  |
| 60 | Gebrie, Alemu, Animut Alebel, Abriham Zegeye, Bekele Tesfaye, and Fasil Wagnew. "Prevalence and associated factors of active trachoma among children in Ethiopia: a systematic review and meta-analysis." *BMC infectious diseases* 19 (2019): 1-12. | Excluded | Duplicate |
| 61 | Astale T, Ebert CD, Nute AW, Zerihun M, Gessese D, Melak B, Sata E, Ayele Z, Ayenew G, Callahan EK, Haile M. The population-based prevalence of trachomatous scarring in a trachoma hyperendemic setting: results from 152 impact surveys in Amhara, Ethiopia. BMC ophthalmology. 2021 May 13;21(1):213. | Excluded | Outcome of interest not reported |
| 62 | Belsti Y, Fekadu SA, Assem AS. Active trachoma prevalence and its associated factors among children aged 1-9 years in rural residents of Lare District, Southwest Ethiopia. International Journal of Ophthalmology. 2021;14(11):1756. | Included |  |
| 63 | Getachew D, Woldekidan F, Ayele G, Bekele Y, Sleshi S, Tekalgn E, et al. High prevalence of active trachoma and associated factors among school-aged children in Southwest Ethiopia. PLOS Neglected Tropical Diseases. 2023;17(12):e0011846. | Included |  |
| 64 | Kassim, Kemal, et al. "Prevalence of active trachoma and associated risk factors among children of the pastoralist population in Madda Walabu rural district, Southeast Ethiopia: a community-based cross-sectional study." *BMC infectious diseases* 19 (2019): 1-7. | Exluded | Duplicate |
| 65 | Delelegn D, Tolcha A, Beyene H, Tsegaye B. Status of active trachoma infection among school children who live in villages of open field defecation: a comparative cross-sectional study. BMC public health. 2021 Dec;21:1-0. | Excluded | Duplicate |
| 66 | Kassim, Kemal, et al. "Prevalence of active trachoma and associated risk factors among children of the pastoralist population in Madda Walabu rural district, Southeast Ethiopia: a community-based cross-sectional study." *BMC infectious diseases* 19 (2019): 1-7. | Excluded | Duplicate |
| 67 | Asmare, Z. A., Seifu, B. L., Mare, K. U., Asgedom, Y. S., Kase, B. F., Shibeshi, A. H., ... & Asebe, H. A. (2023). Prevalence and associated factors of active trachoma among 1–9 years of age children in Andabet district, northwest Ethiopia, 2023: A multi-level mixed-effect analysis. *PLOS Neglected Tropical Diseases*, *17*(8), e0011573. | Excluded | Duplicate |
| 68 | Flueckiger, Rebecca Mann, Emanuele Giorgi, Jorge Cano, Mariamo Abdala, Olga Nelson Amiel, Gilbert Baayenda, Ana Bakhtiari et al. "Understanding the spatial distribution of trichiasis and its association with trachomatous inflammation—follicular." BMC infectious diseases 19 (2019): 1-16. | Excluded | Duplicate |
| 69 | Getachew, Dawit, et al. "High prevalence of active trachoma and associated factors among school-aged children in Southwest Ethiopia." PLOS Neglected Tropical Diseases 17.12 (2023): e0011846. | Excluded | Duplicate |
| 70 | ABDULWHAB NA, ELHAG WI. Molecular Detection of Chlamydia Trachomatis Associated with Ocular Infection among Children in Gadarif State-Sudan. | Excluded | Outcome of interest not reported and the study done outside Ethiopia |
| 71 | Mohamed H, Weldegebreal F, Mohammed J, Gemechu A. Trachoma and Associated Factors among School Age Children 4-9 Years in Dire Dawa Administration, Eastern Ethiopia. East African Journal of Health and Biomedical Sciences. 2019;3(2):45-54. | Included |  |
| 72 | Delelegn D, Tolcha A, Beyene H, Tsegaye B. Status of active trachoma infection among school children who live in villages of open field defecation: a comparative cross-sectional study. BMC public health. 2021;21:1-10. | Included |  |
| 73 | Olamiju F, Isiyaku S, Olobio N, Mogaji H, Achu I, Muhammad N, Boyd S, Bakhtiari A, Ebenezer A, Jimenez C, Solomon AW. Prevalence of Trachoma following implementation of the SAFE strategy in three local government areas of Taraba State, North Eastern Nigeria. Ophthalmic epidemiology. 2023 Nov 2;30(6):619-27. | Excluded | study done outside Ethiopia |
| 74 | Chen, Xinyi, Beatriz Munoz, Harran Mkocha, Meraf A. Wolle, and Sheila K. West. "Children as messengers of health knowledge? Impact of health promotion and water infrastructure in schools on facial cleanliness and trachoma in the community." *PLoS neglected tropical diseases* 15, no. 2 (2021): e0009119. | Excluded | Outcome of interest not reported and the study done outside Ethiopia |
| 75 | Altaseb T, Lingerew M, Adane M. Prevalence of trachomatous inflammation-follicular and associated factors among children aged 1-9 years in northeastern Ethiopia. BMC pediatrics. 2024 Feb 19;24(1):128. | Excluded | Duplicate |
| 76 | Tanywe, A.C., Matchawe, C., Fernandez, R. and Lapkin, S., 2019. Perceptions and practices of community members relating to trachoma in Africa: a qualitative systematic review protocol. *JBI Evidence Synthesis*, *17*(11), pp.2350-2356. | Excluded | Outcome of interest not reported and the study done outside Ethiopia |
| 77 | Seyum D, Fetene N, Kifle T, Negash H, Kabeto T, Gebre M, Data T, Tadele T, Abayo G, Wondimu A, Butcher R. Prevalence of trachoma from 66 impact surveys in 52 woredas of Southern Nations, Nationalities and Peoples’ and Sidama Regions of Ethiopia, 2017–2019. Ophthalmic epidemiology. 2023 Nov 2;30(6):637-46. | Excluded | Duplicate |
| 78 | Ramadhani, A.M., Derrick, T., Macleod, D., Massae, P., Mafuru, E., Malisa, A., Mbuya, K., Roberts, C.H., Makupa, W., Mtuy, T. and Bailey, R.L., 2019. Progression of scarring trachoma in Tanzanian children: A four-year cohort study. *PLoS neglected tropical diseases*, *13*(8), p.e0007638. | Excluded | Outcome of interest not reported and the study done outside Ethiopia |
| 79 | Tuke D, Etu E, Shalemo E. Active trachoma prevalence and related variables among children in a pastoralist community in southern Ethiopia in 2021: a community-based cross-sectional study. The American Journal of Tropical Medicine and Hygiene. 2023 Jan 9;108(2):252. | Excluded | Duplicate |
| 80 | Caplan N, Sanka BC, Mulat A, Brener DT, Baum S, Seifu A, Kesete NZ, Bruck M, Wohlgemuth LG, Debela MM, Weekes RB. Motivating school communities towards behavior change and local ownership: a gamification intervention to prevent trachoma at primary schools in southern Ethiopia. International Health. 2023 Dec;15(Supplement_2):ii38-43. | Excluded | Outcome of interest not reported |
| 81 | Stakeholder’s perception on the slow progress towards trachoma elimination and suggested recommendations for future intervention: An interpretive qualitative study in Bugna District, Northeast Ethiopia | Excluded | Outcome of interest not reported |
| 82 | Nasir MA, Elsawy F, Omar A, Haque SO, Nadir R. Eliminating trachoma by 2020: assessing progress in Nigeria. Cureus. 2020 Jul 29;12(7). | Excluded | Outcome of interest not reported and the study done outside Ethiopia |
| 83 | Tefera, A., Tadesse, F., Seife, F. and Mamuye, N., 2022. Modeling time to stop trachoma MDA in persistent districts of Ethiopia (Comparison of cox proportional hazard regression and machine learning models). | Excluded | Outcome of interest not reported |
| 84 | Melkie, Gashaw, Muluken Azage, and Genet Gedamu. "Prevalence and associated factors of active trachoma among children aged 1-9 years old in mass drug administration graduated and non-graduated districts in Northwest Amhara region, Ethiopia: A comparative cross-sectional study." *Plos one* 15.12 (2020): e0243863. | Excluded | Duplicate |
| 85 | Gross, L.L., 2019. *Oromo Ethiopians Perceptions of the Prevalence, Causes, Treatment and Prevention of Trachoma* (Doctoral dissertation, Walden University). | Excluded | Outcome of interest not reported |
| 86 | Bah MM, Sakho F, Goepogui A, Nieba LC, Cisse A, Courtright P, Harte AJ, Burgert-Brucker C, Jimenez C, Lama PL, Sagno M. The Prevalence of Trachomatous Trichiasis in People Aged 15 Years and Over in Six Evaluation Units of Gaoual, Labé, Dalaba and Beyla Districts, Guinea. Ophthalmic epidemiology. 2024 Nov 1;31(6):526-33. | Excluded | Outcome of interest not reported and the study done outside Ethiopia |
| 87 | Evidence for contamination with C. trachomatis in the household environment of children with active Trachoma: A cross-sectional study in Kongwa, Tanzania | Excluded | Outcome of interest not reported and the study done outside Ethiopia |
| 88 | Gower EW, Munoz B, Rajak S, Habtamu E, West SK, Merbs SL, Harding JC, Alemayehu W, Callahan EK, Emerson PM, Gebre T. Pre-operative trichiatic eyelash pattern predicts post-operative trachomatous trichiasis. PLoS neglected tropical diseases. 2019 Oct 7;13(10):e0007637. | Excluded | Outcome of interest not reported |
| 89 | West SK. Milestones in the fight to eliminate trachoma. Ophthalmic and Physiological Optics. 2020 Mar;40(2):66-74. | Excluded | Outcome of interest not reported |
| 90 | Oldenburg CE, Aragie S, Amza A, Solomon AW, Brogdon J, Arnold BF, Keenan JD, Lietman TM. Can we eradicate trachoma? A survey of stakeholders. British Journal of Ophthalmology. 2021 Aug 1;105(8):1059-62. | Excluded | Outcome of interest not reported |
| 91 | Wolle, M.A., Muñoz, B.E., Naufal, F., Kashaf, M.S., Mkocha, H. and West, S.K., 2021. Risk factors for the progression of trachomatous scarring in a cohort of women in a trachoma low endemic district in Tanzania. *PLoS neglected tropical diseases*, *15*(11), p.e0009914. | Excluded | study done outside Ethiopia |
| 92 | Burr SE, Hart J, Samikwa L, Chaima D, Cooley G, Martin D, Masika M, Solomon AW, Bailey RL, Kalua K. Pgp3 seroprevalence and associations with active trachoma and ocular Chlamydia trachomatis infection in Malawi: cross-sectional surveys in six evaluation units. PLoS neglected tropical diseases. 2019 Oct 28;13(10):e0007749. | Excluded | Outcome of interest not reported and the study done outside Ethiopia |
| 93 | Lietman TM, Ayele B, Gebre T, Zerihun M, Tadesse Z, Emerson PM, Nash SD, Porco TC, Keenan JD, Oldenburg CE. Frequency of mass azithromycin distribution for ocular chlamydia in a trachoma endemic region of Ethiopia: a cluster randomized trial. American journal of ophthalmology. 2020 Jun 1;214:143-50. | Excluded | Outcome of interest not reported |
| 94 | Gebretnsae H, Mamo N, Teklemariam T, Fenta K, Gebrehiwet T, Berhe A, Gebreselasie F, Demoz K. Knowledge, attitudes, and practices about trachoma in rural communities of Tigray Region, Northern Ethiopia: implications for prevention and control. Journal of environmental and public health. 2020;2020(1):3270530. | Excluded | Outcome of interest not reported |
| 95 | Kassaw MW, Tegegne KM, Ahmed M, Tegegne KD. Stakeholder’s perspective on the slow progress towards elimination of trachoma as a public health problem, and suggested recommendations for future intervention improvements: A pilot case study in Bugna district, 2020, Northeast Ethiopia. | Excluded | Outcome of interest not reported |
| 96 | Asmare, Z. A., Seifu, B. L., Mare, K. U., Asgedom, Y. S., Kase, B. F., Shibeshi, A. H., ... & Asebe, H. A. (2023). Prevalence and associated factors of active trachoma among 1–9 years of age children in Andabet district, northwest Ethiopia, 2023: A multi-level mixed-effect analysis. *PLOS Neglected Tropical Diseases*, *17*(8), e0011573. | Excluded | Duplicate |
| 97 | de Brito CM, Barbosa CC, de Andrade SM, de Oliveira AL, Montarroyos UR, Ferraz C, de Toledo Vieira M, Lopes MD, Gouveia GC, de Medeiros ZM. Household survey of trachoma among children living in Pernambuco, Brazil. Pathogens. 2019 Nov 25;8(4):263. | Excluded | study done outside Ethiopia |
| 98 | Martin DL, Saboyà-Díaz MI, Abashawl A, Alemayeh W, Gwyn S, Hooper PJ, Keenan J, Kalua K, Szwarcwald CL, Nash S, Oldenburg C. The use of serology for trachoma surveillance: Current status and priorities for future investigation. PLoS neglected tropical diseases. 2020 Sep 24;14(9):e0008316. | Excluded | Outcome of interest not reported |
| 99 | Sasanami M, Amoah B, Diori AN, Amza A, Souley AS, Bakhtiari A, Kadri B, Szwarcwald CL, Ferreira Gomez DV, Almou I, Lopes MD. Using model-based geostatistics for assessing the elimination of trachoma. PLoS neglected tropical diseases. 2023 Jul 28;17(7):e0011476. | Excluded | Outcome of interest not reported |
| 100 | Geleta, Daniel, and Netsanet Workneh. "Prevalence and Determinants of Trachomatous Trichiasis towards Elimination of Trachoma in Ethiopia: A Systematic Review and Meta-Analysis." (2021). | Excluded | Outcome of interest not reported |
| 101 | Mekonnen, Jemal, Jeylan Kassim, Muluneh Ahmed, and Negeso Gebeyehu. "Prevalence of active Trachoma and associated factors among children 1–9 years old at Arsi Negele town, West Arsi zone, Oromia regional state, Southern Ethiopia." *Plos one* 17, no. 10 (2022): e0273808. | Excluded | Duplicate |
| 102 | Wu TJ, Reynolds MM. Trachoma, the world's leading infectious cause of blindness: The remaining gap in care and access to basic handwashing facilities. European Journal of Ophthalmology. 2023 Jul;33(4):1576-82. | Excluded | Outcome of interest not reported |
| 103 | Caplan N, Sanka BC, Mulat A, Brener DT, Baum S, Seifu A, Kesete NZ, Bruck M, Wohlgemuth LG, Debela MM, Weekes RB. Motivating school communities towards behavior change and local ownership: a gamification intervention to prevent trachoma at primary schools in southern Ethiopia. International Health. 2023 Dec;15(Supplement_2):ii38-43. | Excluded | Outcome of interest not reported |
| 104 | Churko C, Bekele Kassahun A, Getachew T, Bokicho B, Terefe HD, Dagne S, Yohanes T. Prevalence of Post-Operative Trichiasis in Southern Ethiopia, 2021: A Community Based Cross Sectional Study. Clinical Ophthalmology. 2023 Dec 31:2975-82. | Excluded | Outcome of interest not reported |
| 105 | Tadesse Z, Callahan K, Emerson PM, Aragie S, Gebeyehu W, Freeman MC, Cevallos V, Melo J, Wittberg DM, Porco TC, Lietman TM. Sanitation, Water, and Instruction in Face-washing for Trachoma I. | Excluded | Outcome of interest not reported |
| 106 | Abebe, Thomas Ayalew, and Gudina Terefe Tucho. "The impact of access to water supply and sanitation on the prevalence of active trachoma in Ethiopia: A systematic review and meta-analysis." *PLoS Neglected Tropical Diseases* 15, no. 9 (2021): e0009644. | Excluded | Duplicate |
| 107 | Maciel AM, Almeida NM, Silva AC, Almeida PC. Factors associated with trachoma treatment and control treatment in schools of municipality of the Northeast Region, Brazil. Revista Brasileira de Epidemiologia. 2020 Feb 21;23:e200011. | Excluded | Outcome of interest not reported and the study done outside Ethiopia |
| 108 | Amechi NJ, Odama RI, Agada SA, Kenechukwu CO, Ezeh CO. Scopus Review of the Incidence, Treatment and Control of the Spread of Trachoma Species in Nigeria in the Past 5 Decades. Am J Med Public Health. 2021; 2 (4).;1026. | Excluded | Outcome of interest not reported |
| 109 | Baayenda G, Mugume F, Mubangizi A, Turyaguma P, Tukahebwa EM, Byakika S, Kahwa B, Kusasira D, Bakhtiari A, Boyd S, Butcher R. Baseline prevalence of trachoma in refugee settlements in Uganda: Results of 11 population-based surveys. Ophthalmic Epidemiology. 2023 Nov 2;30(6):580-90. | Excluded | study done outside Ethiopia |
| 110 | Delelegn D, Tolcha A, Beyene H, Tsegaye B. Status of active trachoma infection among school children who live in villages of open field defecation: a comparative cross-sectional study. BMC public health. 2021 Dec;21:1-0. | Excluded | Duplicate |
| 111 | Miller HA, López de Mesa CB, Talero SL, Meza Cárdenas M, Ramírez SP, Moreno-Montoya J, Porras A, Trujillo-Trujillo J. Prevalence of trachoma and associated factors in the rural area of the department of Vaupés, Colombia. PLoS One. 2020 May 19;15(5):e0229297. | Excluded | study done outside Ethiopia |
| 112 | \| Abayo G, Gessesse GW, Asaminew T. Prevalence and pattern of ocular morbidity among school children in southern Ethiopia. Ethiopian Journal of Health Sciences. 2021 Jul 1;31(4). \| \| --- \| | Excluded | Duplicate |
| 113 | Saboyá-Díaz MI, Carey Angeles CA, Avellaneda Yajahuanca RD, Meléndez Ruíz SK, Cabrera R, Honorio Morales HA, Pachas PE, Guardo M, Renneker KK, Muñoz BE, West SK. Associated factors of the co-occurrence of trachoma and soil-transmitted helminthiases in children 1 to 9 years old in rural communities of the Amazon basin in Loreto Department, Peru: Results from a population-based survey. PLoS neglected tropical diseases. 2022 Jul 25;16(7):e0010532. | Excluded | study done outside Ethiopia |
| 114 | CELESTINE A, FELIX H, DOREEN O. Factors Predisposing Children Aged 0-9 Years to Trachoma in Kirindon Division, Trans Mara Sub-County, Kenya. eajahme. 2021 Aug 1;5(5). | Excluded | study done outside Ethiopia |
| 115 | Belsti, Y., Fekadu, S.A. and Assem, A.S., 2021. Active trachoma prevalence and its associated factors among children aged 1-9 years in rural residents of Lare District, Southwest Ethiopia. *International Journal of Ophthalmology*, *14*(11), p.1756. | Excluded | Duplicate |
| 116 | Eye diseases and Blindness Alemayehu, Wondu; Cherinet, Assefa the Ecology Of Health And Disease In Ethiopia 2019;():237-250 Routledge 2019 | Excluded | Outcome of interest not reported |
| 117 | Bucumi V, Muhimpundu E, Issifou AA, Akweyu S, Burn N, Willems J, Niyongabo J, Elvis A, Koizan G, Harte A, Boyd S. Baseline, Impact and Surveillance Trachoma Prevalence Surveys in Burundi, 2018–2021. Ophthalmic Epidemiology. 2024 Nov 1;31(6):543-52. | Excluded | study done outside Ethiopia |
| 118 | Mwangi G, Harding-Esch E, Kabona G, Watitu T, Mpyet C, Gemechu A, Abdeta A, Wamyil-Mshelia T, Ajege G, Kelly M, Abony M. Explaining the continuing high prevalence of trachomatous trichiasis unknown to the health system in evaluation units: a mixed methods explanatory study in four trachoma-endemic countries. International health. 2023 Dec;15(Supplement_2):ii44-52. | Excluded | study done outside Ethiopia |
| 119 | Tuke D, Etu E, Shalemo E. Active trachoma prevalence and related variables among children in a pastoralist community in southern Ethiopia in 2021: a community-based cross-sectional study. The American Journal of Tropical Medicine and Hygiene. 2023 Jan 9;108(2):252. | Excluded | Duplicate |
| 120 | Maritim P, Zulu JM, Jacobs C, Chola M, Chongwe G, Zyambo J, Halwindi H, Michelo C. Factors shaping the implementation of the SAFE strategy for trachoma using the Consolidated Framework for Implementation Research: a systematic review. Global health action. 2019 Jan 1;12(1):1570646. | Excluded | Wrong study design |
| 121 | Greenland K, White S, Sommers K, Biran A, Burton MJ, Sarah V, Alemayehu W. Selecting behaviour change priorities for trachoma ‘F’and ‘E’interventions: A formative research study in Oromia, Ethiopia. PLoS neglected tropical diseases. 2019 Oct 9;13(10):e0007784. | Excluded | Outcome of interest not reported |
| 122 | Renneker KK, Abdala M, Addy J, Al-Khatib T, Amer K, Badiane MD, Batcho W, Bella L, Bougouma C, Bucumi V, Chisenga T. Global progress toward the elimination of active trachoma: an analysis of 38 countries. The Lancet Global Health. 2022 Apr 1;10(4):e491-500. | Excluded | Outcome of interest not reported and the study done outside Ethiopia |
| 123 | Elshafie BE, Elsanosi MS, El Amin A, Butcher R, Willis R, Bakhtiari A, Jimenez C, Dejene M, Solomon AW, Harding-Esch EM, Binnawi KH. Trachoma prevalence in four localities of Darfur Region, Sudan, following one round of antibiotic mass drug administration. Ophthalmic Epidemiology. 2023 Nov 2;30(6):571-9. | Excluded | study done outside Ethiopia |
| 124 | Kebede F, Jamal M. Retracted: Prevalence of active trachoma infection and associated factors post‐war resettled population in raya kobo districts, North East Ethiopia: A community‐based cross‐sectional study in 2022. Health Science Reports. 2023 Aug;6(8):e1486. | Excluded | Retracted |
| 125 | Szwarcwald CL, Lopes MD, Borges de Souza Junior PR, Vaz Ferreira Gómez D, Luna EJ, da Silva de Almeida W, Damacena GN, Ribeiro Favacho JD, Germano de Frias P, Butcher R, Boyd S. Population prevalence of trachoma in nine rural non-indigenous evaluation units of Brazil. Ophthalmic Epidemiology. 2023 Nov 2;30(6):561-70. | Excluded | study done outside Ethiopia |
| 126 | Mahmud H, Haile BA, Tadesse Z, Gebresillasie S, Shiferaw A, Zerihun M, Liu Z, Callahan EK, Cotter SY, Varnado NE, Oldenburg CE. Targeted Mass Azithromycin Distribution for Trachoma: A Community-Randomized Trial (TANA II). Clinical Infectious Diseases. 2023 Aug 1;77(3):388-95. | Excluded | Wrong study design |
| 127 | Delelegn D, Tolcha A, Beyene H, Tsegaye B. Status of active trachoma infection among school children who live in villages of open field defecation: a comparative cross-sectional study. BMC public health. 2021 Dec;21:1-0. | Excluded | Duplicate |
| 128 | Flueckiger RM, Courtright P, Abdala M, Abdou A, Abdulnafea Z, Al-Khatib TK, Amer K, Amiel ON, Awoussi S, Bakhtiari A, Batcho W. The global burden of trichiasis in 2016. PLoS neglected tropical diseases. 2019 Nov 25;13(11):e0007835. | Excluded | Outcome of interest not reported |
| 129 | Atekem K, Harding-Esch EM, Martin DL, Downs P, Palmer SL, Kaboré A, Kelly M, Bovary A, Sarr A, Nguessan K, James F. High prevalence of trachomatous inflammation–follicular with no trachomatous trichiasis: can alternative indicators explain the epidemiology of trachoma in Côte d’Ivoire?. International Health. 2023 Dec;15(Supplement_2):ii3-11. | Excluded | The study done outside Ethiopia |
| 130 | Bilchut AH, Burroughs HR, Oldenburg CE, Lietman TM. Trachoma Control: A Glass Half Full?. The American Journal of Tropical Medicine and Hygiene. 2023 Jan 9;108(2):237. | Excluded | Outcome of interest not reported |
| 131 | Reacher MH, Muñoz B, Alghassany A, Daar AS, Elbualy M, Taylor HR. A controlled trial of surgery for trachomatous trichiasis of the upper lid. Archives of Ophthalmology. 1992 May 1;110(5):667-74. | Excluded | Non observational Study design and study period ouside eligible period |
| 132 | Tanywe AC, Green H, Fernandez R. Perceptions and practices of community members relating to trachoma in Africa: a qualitative systematic review. JBI evidence synthesis. 2022 Oct 1;20(10):2445-74. | Excluded | Outcome of interest not reported |
| 133 | Borlase A, Blumberg S, Callahan EK, Deiner MS, Nash SD, Porco TC, Solomon AW, Lietman TM, Prada JM, Hollingsworth TD. Modelling trachoma post-2020: opportunities for mitigating the impact of COVID-19 and accelerating progress towards elimination. Transactions of the Royal Society of Tropical Medicine and Hygiene. 2021 Mar;115(3):213-21. | Excluded | Outcome of interest not reported |
| 134 | Kassaw, M. W., Abebe, A. M., Tegegne, K. D., Getu, M. A., & Bihonegn, W. T. (2020). Prevalence and associations of active trachoma among rural preschool children in Wadla district, northern Ethiopia. *BMC ophthalmology*, *20*, 1-10. | Excluded | Duplicate |
| 135 | Senyonjo L, Aboe A, Bailey R, Agyemang D, Marfo B, Wanye S, Schmidt E, Addy J, Blanchet K. Operational adaptations of the trachoma pre-validation surveillance strategy employed in Ghana: a qualitative assessment of successes and challenges. Infectious diseases of poverty. 2019 Dec;8:1-1. | Excluded | Outcome of interest not reported and the study done outside Ethiopia |
| 136 | Environmental factors and hygiene behaviors associated with facial cleanliness and trachoma in Kongwa, Tanzania | Excluded | Outcome of interest not reported and the study done outside Ethiopia |
| 137 | Shobiso MG, Hussen MS, Munaw MB, Tilahun MM. Trachoma prevention practice and associated factors in rural Lemo District, southern Ethiopia, 2021. Ethiopian Journal of Health Sciences. 2023 Jan 1;33(1). | Excluded | Outcome of interest not reported |
| 138 | Czerniewska A, Versteeg A, Shafi O, Dumessa G, Aga MA, Last A, MacLeod D, Sarah V, Dodson S, Negussu N, Sori BK. Comparison of face washing and face wiping methods for trachoma control: a pilot study. The American journal of tropical medicine and hygiene. 2020 Feb 10;102(4):740. | Excluded | Outcome of interest not reported |
| 140 | Tedijanto C, Aragie S, Tadesse Z, Haile M, Zeru T, Nash SD, Wittberg DM, Gwyn S, Martin DL, Sturrock HJ, Lietman TM. Predicting future ocular Chlamydia trachomatis infection prevalence using serological, clinical, molecular, and geospatial data. medRxiv. 2021 Jul 22:2021-07. | Excluded | Outcome of interest not reported |
| 141 | Tedijanto C, Aragie S, Tadesse Z, Haile M, Zeru T, Nash SD, Wittberg DM, Gwyn S, Martin DL, Sturrock HJ, Lietman TM. Predicting future community-level ocular Chlamydia trachomatis infection prevalence using serological, clinical, molecular, and geospatial data. PLoS neglected tropical diseases. 2022 Mar 11;16(3):e0010273. | Excluded | Outcome of interest not reported |
| 142 | Sullivan KM, Harding-Esch EM, Keil AP, Freeman MC, Batcho WE, Bio Issifou AA, Bucumi V, Bella AL, Epee E, Bobo Barkesa S, Seife Gebretsadik F. Exploring water, sanitation, and hygiene coverage targets for reaching and sustaining trachoma elimination: G-computation analysis. PLoS neglected tropical diseases. 2023 Feb 13;17(2):e0011103. | Excluded | Outcome of interest not reported |
| 143 | Genet, A., Dagnew, Z., Melkie, G., Keleb, A., Motbainor, A., Mebrat, A. and Leshargie, C.T., 2022. Prevalence of active trachoma and its associated factors among 1–9 years of age children from model and non-model kebeles in Dangila district, northwest Ethiopia. *Plos one*, *17*(6), p.e0268441. | Excluded | Duplicate |
| 144 | Xiong T, Yue Y, Li WX, Choonara I, Qazi S, Chen HJ, Tang J, Shi J, Wang H, Zeng LN, Xia B. Effectiveness of azithromycin mass drug administration on trachoma: a systematic review. Chinese medical journal. 2021 Dec 20;134(24):2944-53. | Excluded | Outcome of interest not reported and the study done outside Ethiopia |
| 145 | Renneker KK, Abdala M, Addy J, Al-Khatib T, Amer K, Badiane MD, Batcho W, Bella L, Bougouma C, Bucumi V, Chisenga T. Elimination Delayed is Not Elimination Denied: Progress Toward GET2020. | Excluded | Outcome of interest not reported |
| 146 | Hammou J, Guagliardo SA, Obtel M, Razine R, Haroun AE, Youbi M, Bellefquih AM, White M, Gwyn S, Martin DL. Post-validation survey in two districts of Morocco after the elimination of trachoma as a public health problem. The American Journal of Tropical Medicine and Hygiene. 2022 Mar 28;106(5):1370. | Excluded | Outcome of interest not reported and the study done outside Ethiopia |
| 147 | Mahmud H, Haile BA, Tadesse Z, Gebresillasie S, Shiferaw A, Zerihun M, Keenan JD, Lietman T. Comparing targeted azithromycin treatment strategies in a trachoma hyperendemic area. Investigative Ophthalmology & Visual Science. 2022 Jun 1;63(7):3565-A0452. | Excluded | Outcome of interest not reported |
| 148 | WoldeKidan E, Daka D, Legesse D, Laelago T, Betebo B. Prevalence of active trachoma and associated factors among children aged 1 to 9 years in rural communities of Lemo district, southern Ethiopia: community based cross sectional study. BMC infectious diseases. 2019 Dec;19:1-8. | Excluded | Duplicate |
| 149 | Saber Osman D, El-Sayed Hassan S, Mohammed Said K, Mohammed Abd El-Aziz S. Mothers' Knowledge and Practices regarding Care of their Children Suffering from Blinding Trachoma. Journal of Nursing Science Benha University. 2022 Jan 1;3(1):165-77. | Excluded | Outcome of interest not reported and the study done outside Ethiopia |
| 150 | Alshamahi EY, Al-Eryani SA, Jaadan BM, Al-Shamahy HA, Al Haddad AA, Al-Zazai BA. The national campaign for the mass treatment of trachoma: monitoring coverage and practices of Mass Drug Administration (MDA) in Yemen-follow-up study. The national campaign for the mass treatment of trachoma: monitoring coverage and practices of Mass Drug Administration (MDA) in Yemen-follow-up study. 2021. | Excluded | Outcome of interest not reported and the study done outside Ethiopia |
| 151 | Macleod CK, Binnawi KH, Elshafie BE, Sadig HE, Hassan A, Cocks N, Willis R, Chu B, Solomon AW, Global Trachoma Mapping Project. Unimproved water sources and open defecation are associated with active trachoma in children in internally displaced persons camps in the Darfur States of Sudan. Transactions of the Royal Society of Tropical Medicine and Hygiene. 2019 Oct 11;113(10):599-609. | Excluded | Outcome of interest not reported and the study done outside Ethiopia |
| 152 | Tedijanto C, Solomon AW, Martin DL, Nash SD, Keenan JD, Lietman TM, Lammie PJ, Aiemjoy K, Amza A, Aragie S, Arzika AM. Monitoring transmission intensity of trachoma with serology. Nature communications. 2023 Jun 5;14(1):3269. | Excluded | Duplicate |
| 153 | Ten Years After Mass Treatment with Two Doses of Azithromycin for Trachoma Elimination in Rombo District–Kilimanjaro: Is Trachoma Still Eliminated? A Case Study of Kahe Mpya Sub-Village | Excluded | study done outside Ethiopia |
| 154 | Keenan JD, Gebresillasie S, Stoller NE, Haile BA, Tadesse Z, Cotter SY, Ray KJ, Aiemjoy K, Porco TC, Callahan EK, Emerson PM. Linear growth in preschool children treated with mass azithromycin distributions for trachoma: A cluster-randomized trial. PLoS neglected tropical diseases. 2019 Jun 5;13(6):e0007442. | Excluded | Outcome of interest not reported |
| 155 | Tuke D, Etu E, Shalemo E. Active trachoma prevalence and related variables among children in a pastoralist community in southern Ethiopia in 2021: a community-based cross-sectional study. The American Journal of Tropical Medicine and Hygiene. 2023 Jan 9;108(2):252. | Excluded | Duplicate |
| 156 | Bekuma TT, Mosisa Kebebew G, Desalegn Waktole Z, Markos Cafo J, Wirtu D, Gaddisa S. Coverage assessment survey following trachoma mass drug administration (MDA) in six districts of Oromia, Western Ethiopia, 2017. PLoS neglected tropical diseases. 2019 Dec 16;13(12):e0007924. | Excluded | Outcome of interest not reported |
| 157 | Greenland K, Czerniewska A, Guye M, Legesse D, Ahmed Mume A, Shafi Abdurahman O, Abraham Aga M, Miecha H, Shumi Bejiga G, Sarah V, Burton M. Seasonal variation in water use for hygiene in Oromia, Ethiopia, and its implications for trachoma control: An intensive observational study. PLoS neglected tropical diseases. 2022 May 13;16(5):e0010424. | Excluded | Outcome of interest not reported |
| 158 | Amza A, Kadri B, Nassirou B, Arzika AM, Austin A, Nyatigo F, Lebas E, Arnold BF, Lietman TM, Oldenburg CE. Azithromycin Reduction to Reach Elimination of Trachoma (ARRET): study protocol for a cluster randomized trial of stopping mass azithromycin distribution for trachoma. BMC ophthalmology. 2021 Dec;21:1-6. | Excluded | Outcome of interest not reported and the study done outside Ethiopia |
| 159 | Melkie, Gashaw, Muluken Azage, and Genet Gedamu. "Prevalence and associated factors of active trachoma among children aged 1-9 years old in mass drug administration graduated and non-graduated districts in Northwest Amhara region, Ethiopia: A comparative cross-sectional study." *Plos one* 15.12 (2020): e0243863. | Excluded | Duplicate |
| 160 | O'Brien KS, Emerson P, Hooper PJ, Reingold AL, Dennis EG, Keenan JD, Lietman TM, Oldenburg CE. Antimicrobial resistance following mass azithromycin distribution for trachoma: a systematic review. The Lancet Infectious Diseases. 2019 Jan 1;19(1):e14-25. | Excluded | Outcome of interest not reported and non-observational design |
| 161 | Nayel Y, Taylor M, Montasser AS, Elsherif M, Diab MM. Perceptions of ophthalmologists on the impact of trachoma in Egypt: a mixed-methods, nationwide survey. BMC Infectious Diseases. 2023 Jan 17;23(1):27. | Excluded | Outcome of interest not reported and the study done outside Ethiopia |
| 162 | World Health Organization. Report of the 21st meeting of the WHO alliance for the global elimination of trachoma by 2020, Geneva, Switzerland, 20-22 April 2017. InReport of the 21st meeting of the WHO alliance for the global elimination of trachoma by 2020, Geneva, Switzerland, 20-22 April 2017 2019. | Excluded | Wrong study design |
| 163 | Flueckiger, R.M., 2019. *Exploring the spatial heterogeneity of trachomatous trichiasis* (Doctoral dissertation, London School of Hygiene & Tropical Medicine). | Excluded | Outcome of interest not reported and the study done outside Ethiopia |
| 164 | Genet, A., Dagnew, Z., Melkie, G., Keleb, A., Motbainor, A., Mebrat, A. and Leshargie, C.T., 2022. Prevalence of active trachoma and its associated factors among 1–9 years of age children from model and non-model kebeles in Dangila district, northwest Ethiopia. *Plos one*, *17*(6), p.e0268441. | Excluded | Duplicate |
| 165 | \| Churko C, Asfaw MA, Zerdo Z. Knowledge and attitude of community towards trachoma and trichiasis in Arba Minch Zuria district, Gamo Zone, Southern Ethiopia, 2019. \| \| --- \| | Excluded | Outcome of interest not reported |
| 166 | Sata E, Seife F, Ayele Z, Murray SA, Wickens K, Le P, Zerihun M, Melak B, Chernet A, Jensen KA, Gessese D. Wait and watch: A trachoma surveillance strategy from Amhara region, Ethiopia. PLOS Neglected Tropical Diseases. 2024 Feb 22;18(2):e0011986. | Excluded | Outcome of interest not reported |
| 167 | Karmaoui A, El Jaafari S, Chaachouay H, Hajji L. Socio-ecological factors influencing vulnerability to trachoma disease: a new tool applied in five pre-saharan provinces, Morocco. GeoJournal. 2023 Jun;88(3):2669-90. | Excluded | Outcome of interest not reported and the study done outside Ethiopia |
| 168 | Ageed A, Khan M. Eliminating trachoma in Africa: the importance of environmental interventions. Cureus. 2024 Jan 16;16(1). | Excluded | Outcome of interest not reported and the study done outside Ethiopia |
| 169 | Tidwell JB, Fergus C, Gopalakrishnan A, Sheth E, Sidibe M, Wohlgemuth L, Jain A, Woods G. Integrating face washing into a school-based, handwashing behavior change program to prevent trachoma in Turkana, Kenya. The American journal of tropical medicine and hygiene. 2019 Aug 5;101(4):767. | Excluded | Outcome of interest not reported and the study done outside Ethiopia |
| 170 | Whitson CC, Nute AW, Hailemariam B, Deathe AR, Astale T, Ayele Z, Gessese D, Sata E, Zerihun M, Melak B, Haile M. Photographic grading for trachoma diagnosis within trachoma impact surveys in Amhara region, Ethiopia. Transactions of The Royal Society of Tropical Medicine and Hygiene. 2023 Feb;117(2):111-7. | Excluded | Outcome of interest not reported |
| 171 | Last A, Versteeg B, Shafi Abdurahman O, Robinson A, Dumessa G, Abraham Aga M, Shumi Bejiga G, Negussu N, Greenland K, Czerniewska A, Thomson N. Detecting extra-ocular Chlamydia trachomatis in a trachoma-endemic community in Ethiopia: Identifying potential routes of transmission. PLoS neglected tropical diseases. 2020 Mar 4;14(3):e0008120. | Excluded | Duplictae |
| 172 | Hu V, Caswell R, Last A, Burton M, Mabey D. Trachoma and Inclusion conjunctivitis. InHunter's tropical medicine and emerging infectious diseases 2020 Jan 1 (pp. 421-428). Elsevier. | Excluded | Outcome of interest not reported and the study done outside Ethiopia |
| 173 | Solomon AW, Kello AB, Bangert M, West SK, Taylor HR, Tekeraoi R, Foster A. The simplified trachoma grading system, amended. Bulletin of the World Health Organization. 2020 Sep 3;98(10):698. | Excluded | Outcome of interest not reported |
| 174 | Kashaf MS, Muñoz BE, Mkocha H, Wolle MA, Naufal F, West SK. Incidence and progression of trachomatous scarring in a cohort of children in a formerly hyper-endemic district of Tanzania. PLoS neglected tropical diseases. 2020 Oct 5;14(10):e0008708. | Excluded | Outcome of interest not reported and the study done outside Ethiopia |
| 175 | Renneker KK, Abdala M, Addy J, Al-Khatib T, Amer K, Badiane MD, Batcho W, Bella L, Bougouma C, Bucumi V, Chisenga T. Global progress toward the elimination of active trachoma: an analysis of 38 countries. The Lancet Global Health. 2022 Apr 1;10(4):e491-500. | Excluded | Duplicate |
| 176 | Adafrie Y, Redae G, Zenebe D, Adhena G. Uptake of trachoma trichiasis surgery and associated factors among trichiasis-diagnosed clients in southern Tigray, Ethiopia. Clinical Ophthalmology. 2021 May 10:1939-48. | Excluded | Outcome of interest not reported |
| 177 | Sayed LR, Mohammed A, Ahmed A, D Mohammed M. Effect of Health Educational Program on Mothers’ Knowledge and Practices RegardingCare of Children with Trachoma. Minia Scientific Nursing Journal. 2021 Dec 30;10(1):35-45. | Excluded | Outcome of interest not reported and the study done outside Ethiopia |
| 178 | Muche N, Wasihun Y, Wondiye H, Bogale EK, Anagaw TF. Behavioral Responses for Face Cleanliness Message to Prevent Trachoma Among Mothers Having Children Age 1–9 Years Old, in Fogera District, Northwest Ethiopia: An Application of Extended Parallel Process Model. International Journal of General Medicine. 2023 Dec 31:1927-41. | Excluded | Outcome of interest not reported |
| 179 | Morberg DP, Alemayehu W, Melese M, Lakew T, Sisay A, Zhou Z, Cevallos V, Oldenburg CE, Porco TC, Lietman TM, Keenan JD. A longitudinal analysis of chlamydial infection and trachomatous inflammation following mass azithromycin distribution. Ophthalmic epidemiology. 2019 Jan 2;26(1):19-26. | Excluded | Study conducted outside of the eligible period |
| 180 | Abebe, Thomas Ayalew, and Gudina Terefe Tucho. "The impact of access to water supply and sanitation on the prevalence of active trachoma in Ethiopia: A systematic review and meta-analysis." *PLoS Neglected Tropical Diseases* 15, no. 9 (2021): e0009644. | Excluded | Duplicate |
| 181 | Mahmud H, Landskroner E, Amza A, Aragie S, Godwin WW, de Hostos Barth A, O’Brien KS, Lietman TM, Oldenburg CE. Stopping azithromycin mass drug administration for trachoma: A systematic review. PLoS Neglected Tropical Diseases. 2021 Jul 8;15(7):e0009491. | Excluded | Outcome of interest not reported and non observational design |
| 182 | Belsti, Y., Fekadu, S.A. and Assem, A.S., 2021. Active trachoma prevalence and its associated factors among children aged 1-9 years in rural residents of Lare District, Southwest Ethiopia. *International Journal of Ophthalmology*, *14*(11), p.1756. | Excluded | Duplicate |
| 183 | Gallini JW, Sata E, Zerihun M, Melak B, Haile M, Zeru T, Gessese D, Ayele Z, Tadesse Z, Callahan EK, Nash SD. Optimizing cluster survey designs for estimating trachomatous inflammation–follicular within trachoma control programs. International Journal of Infectious Diseases. 2022 Mar 1;116:101-7. | Excluded | Outcome of interest not reported and the study done outside Ethiopia |
| 184 | Aysheshim, Amsalu. "Prevalence and Associated Factors of Postoperative Trichiasis Among Adults in Ayehu Guagusa District, North-West Ethiopia: Community Based Crosssectional Study." PhD diss., 2022. | Excluded | Outcome of interest not reported |
| 185 | Abebe, Thomas Ayalew, and Gudina Terefe Tucho. "The impact of access to water supply and sanitation on the prevalence of active trachoma in Ethiopia: A systematic review and meta-analysis." *PLoS Neglected Tropical Diseases* 15, no. 9 (2021): e0009644. | Excluded | Duplicate |
| 186 | Abera T, Tilahun W, Waqjira I. Trachoma prevention practice among mothers with child age of under-9 years and factors associated in rural district of Oromia Region, Ethiopia: Community based cross-sectional study. World Journal of Advanced Research and Reviews. 2021;10(1):245-57. | Excluded | Outcome of interest not reported |
| 187 | Abayo G, Gessesse GW, Asaminew T. Prevalence and pattern of ocular morbidity among school children in southern Ethiopia. Ethiopian Journal of Health Sciences. 2021 Jul 1;31(4). | Excluded | Outcome of interest not reported |
| 188 | Yimam, Abdu Tabor, Gizachew Tadesse Wassie, and Getu Degu Alene. "The magnitude and associated factors of postoperative trichiasis among adults who underwent trachomatous trichiasis surgery in Ambassel District, North-East Ethiopia." (2020). | Excluded | Outcome of interest not reported |
| 189 | Wolle MA, Muñoz BE, Mgboji G, Naufal F, Kashaf MS, Mkocha H, West SK. Gender differences in trachomatous scarring prevalence in a formerly trachoma hyperendemic district in Tanzania. PLoS neglected tropical diseases. 2024 Jan 26;18(1):e0011861. | Excluded | Outcome of interest not reported |
| 190 | Kayiwa D, Murungu R, Watako D, Radooli MO, Sembuche J, Bolawole O. Contribution of Hygiene Behavior Change interventions in trachoma elimination efforts in Uganda: A case study of Napak and Nakapiripirit districts. OIDA International Journal of Sustainable Development. 2020 Jul 17;13(02):75-92. | Excluded | Outcome of interest not reported |
| 191 | Kanyi S, Hydara A, Sillah A, Mpyet C, Harte A, Bakhtiari A, Willis R, Jimenez C, Aboe A, Bailey R, Harding-Esch EM. The Gambia Trachomatous Trichiasis Surveys: Results from Five Evaluation Units Confirm Attainment of Trachoma Elimination Thresholds. Ophthalmic epidemiology. 2024 Nov 1;31(6):534-42. | Excluded | Outcome of interest not reported |
| 192 | Solomon AW, Burton MJ, Gower EW, Harding-Esch EM, Oldenburg CE, Taylor HR, Traoré L. Trachoma. Nature Reviews Disease Primers. 2022 May 26;8(1):32. | Excluded | Outcome of interest not reported |
| 193 | Flueckiger RM, Giorgi E, Cano J, Abdala M, Amiel ON, Baayenda G, Bakhtiari A, Batcho W, Bennawi KH, Dejene M, Elshafie BE. Understanding the spatial distribution of trichiasis and its association with trachomatous inflammation—follicular. BMC infectious diseases. 2019 Dec;19:1-6. | Excluded | Outcome of interest not reported |
| 194 | Xiong T, Yue Y, Li W, Zeng L, Choonara I, Qazi S, Tang J, Qu Y, Chen H, Mu D. The Implementation Strategy of Mass Azithromycin Administration on Infectious Diseases Prevalence and Mortality in Children: A Systematic Review and Meta-Analysis. Available at SSRN 3460667. 2019 Sep 24. | Excluded | Outcome of interest not reported |
| 195 | Last A, Versteeg B, Shafi Abdurahman O, Robinson A, Dumessa G, Abraham Aga M, Shumi Bejiga G, Negussu N, Greenland K, Czerniewska A, Thomson N. Detecting extra-ocular Chlamydia trachomatis in a trachoma-endemic community in Ethiopia: Identifying potential routes of transmission. PLoS neglected tropical diseases. 2020 Mar 4;14(3):e0008120. | Excluded | Outcome of interest not reported |
| 196 | Gebretnsae H, Mamo N, Teklemariam T, Fenta K, Gebrehiwet T, Berhe A, Gebreselasie F, Demoz K. Knowledge, attitudes, and practices about trachoma in rural communities of Tigray Region, Northern Ethiopia: implications for prevention and control. Journal of environmental and public health. 2020;2020(1):3270530. | Excluded | Outcome of interest not reported |
| 197 | World Health Organization. Evaluation of the national trachoma programme of Ethiopia. InEvaluation of the national trachoma programme of Ethiopia 2019. | Excluded | Outcome of interest not reported |
| 200 | Renneker KK, Abdala M, Addy J, Al-Khatib T, Amer K, Badiane MD, Batcho W, Bella L, Bougouma C, Bucumi V, Chisenga T. Global progress toward the elimination of active trachoma: an analysis of 38 countries. The Lancet Global Health. 2022 Apr 1;10(4):e491-500. | Excluded | Duplicate |
| 201 | Gebrie A, Alebel A, Zegeye A, Tesfaye B, Wagnew F. Prevalence and associated factors of active trachoma among children in Ethiopia: a systematic review and meta-analysis. BMC infectious diseases. 2019 Dec;19:1-2. | Excluded | Wrong study design and study period outside eligible period |
| 202 | Renneker KK, Emerson PM, Hooper PJ, Ngondi JM. Forecasting the elimination of active trachoma: An empirical model. PLoS Neglected Tropical Diseases. 2022 Jul 11;16(7):e0010563. | Excluded | Outcome of interest not reported |
| 203 | Genet, A., Dagnew, Z., Melkie, G., Keleb, A., Motbainor, A., Mebrat, A. and Leshargie, C.T., 2022. Prevalence of active trachoma and its associated factors among 1–9 years of age children from model and non-model kebeles in Dangila district, northwest Ethiopia. *Plos one*, *17*(6), p.e0268441. | Excluded | Duplicate |
| 204 | Sata E, Nute AW, Astale T, Gessese D, Ayele Z, Zerihun M, Chernet A, Melak B, Jensen KA, Haile M, Zeru T. Twelve-year longitudinal trends in trachoma prevalence among children aged 1–9 years in Amhara, Ethiopia, 2007–2019. The American journal of tropical medicine and hygiene. 2021 Jan 18;104(4):1278. | Excluded | Outcome of interest not reported |
| 205 | Mtuy TB, Burton MJ, Mwingira U, Ngondi JM, Seeley J, Lees S. Knowledge, perceptions and experiences of trachoma among Maasai in Tanzania: Implications for prevention and control. PLoS neglected tropical diseases. 2019 Jun 24;13(6):e0007508. | Excluded | Outcome of interest not reported and the study done outside Ethiopia |
| 206 | Ripotolim MM, Omwenga EO, Mbeke AM. The social demographic factors associated with trachoma infection among children aged 1-9 years in Kapenguria Sub County in West Pokot County; Kenya. East African Medical Journal. 2020;97(9):3020-9. | Excluded | Outcome of interest not reported and the study done outside Ethiopia |
| 207 | Taylor HR, Burton MJ, Haddad D, West S, Wright H. Trachoma. The Lancet. 2014 Dec 13;384(9960):2142-52. | Excluded | Outcome of interest not reported |
| 208 | Evans JR, Solomon AW, Kumar R, Perez Á, Singh BP, Srivastava RM, Harding‐Esch E. Antibiotics for trachoma. Cochrane Database of Systematic Reviews. 2019(9). | Excluded | Outcome of interest not reported |
| 209 | Renneker KK, Abdala M, Addy J, Al-Khatib T, Amer K, Badiane MD, Batcho W, Bella L, Bougouma C, Bucumi V, Chisenga T. Global progress toward the elimination of active trachoma: an analysis of 38 countries. The Lancet Global Health. 2022 Apr 1;10(4):e491-500. | Excluded | Duplicate |
| 210 | Adamu MD, Mohammed Jabo A, Orji P, Zhang Y, Isiyaku S, Olobio N, Muhammad N, Mshelia Auta L, Willis R, Bakhtiari A, Jimenez C. Baseline prevalence of trachoma in 13 local government areas of Borno State, Nigeria. Ophthalmic epidemiology. 2023 Nov 2;30(6):628-36. | Excluded | Outcome of interest not reported and the study done outside Ethiopia |
| 211 | Aragie S, Wittberg DM, Tadesse W, Dagnew A, Hailu D, Chernet A, Melo JS, Aiemjoy K, Haile M, Zeru T, Tadesse Z. Water, sanitation, and hygiene for control of trachoma in Ethiopia (WUHA): a two-arm, parallel-group, cluster-randomised trial. The Lancet Global Health. 2022 Jan 1;10(1):e87-95. | Excluded | Outcome of interest not reported |
| 212 | Brito CM, Medeiros ZM, Barbosa CC, Montarroyos UR, Ferraz C, Vieira MD, Lopes MD, Gouveia GC. Prevalence of trachoma in Pernambuco State, Brazil (2014-2015). Revista do Instituto de Medicina Tropical de São Paulo. 2021 Apr 12;63:e29. | Excluded | Outcome of interest not reported and the study done outside Ethiopia |
| 213 | Semahegn A, Manyazewal T, Getachew E, Fekadu B, Assefa E, Kassa M, Davey G, Hopkins M, Araya M, Woldehanna T, Hanlon C. Burden of neglected tropical diseases and access to medicine and diagnostics in Ethiopia: a scoping review. Systematic Reviews. 2023 Aug 14;12(1):140. | Excluded | Outcome of interest not reported |
| 214 | Harte AJ, Ghasemian E, Pickering H, Houghton J, Chernet A, Sata E, Yismaw G, Zeru T, Tadesse Z, Callahan EK, Nash SD. Unravelling Chlamydia trachomatis diversity in Amhara, Ethiopia: MLVA-ompA sequencing as a molecular typing tool for trachoma. PLOS Neglected Tropical Diseases. 2024 Apr 25;18(4):e0012143. | Excluded | Outcome of interest not reported |
| 215 | Adamu MD, Mohammed Jabo A, Orji P, Zhang Y, Isiyaku S, Olobio N, Muhammad N, Barem B, Willis R, Bakhtiari A, Jimenez C. Baseline prevalence of trachoma in 21 local government areas of Adamawa State, North East Nigeria. Ophthalmic Epidemiology. 2023 Nov 2;30(6):599-607. | Excluded | the study done outside Ethiopia |
| 216 | Hoffman JJ, Habtamu E, Rono H, Tadesse Z, Wondie T, Minas T, Gashaw B, Callahan EK, MacLeod D, Burton MJ. 3D images as a field grader training tool for trachomatous trichiasis: a diagnostic accuracy study in Ethiopia. PLoS neglected tropical diseases. 2019 Jan 24;13(1):e0007104. | Excluded | Outcome of interest not reported |
| 217 | Beyene GA, Beyene NA, Fekadu GA. Factors associated with active trachoma among children in ebinat district, South Gondar Zone, North West Ethiopia: A community-based cross-sectional study. medRxiv. 2022 Feb 7:2022-02. | Excluded | Outcome of interest not reported |
| 218 | Berkley JA. Mass antibiotic distribution to reduce mortality among preschool children?. Archives of disease in childhood. 2019 Mar 1;104(3):227-8. | Excluded | Outcome of interest not reported |
| 219 | Feyisa T, Bekele D, Tura B, Adem A, Nugusu F. To eliminate trachoma: Azithromycin mass drug administration coverage and associated factors among adults in Goro district, Southeast Ethiopia. PLoS Neglected Tropical Diseases. 2022 Jun 27;16(6):e0010169. | Excluded | Outcome of interest not reported |
| 220 | Al-Eryani SA, Alshamahi EY, Al-Shamahy HA, Al-Moyed KA, Al Shawkany AA, Al-Ankoshy AA. Prevalence and risk factors for Trachoma among primary school children in Sana’a city, Yemen. Universal Journal of Pharmaceutical Research. 2021 Sep 15. | Excluded | the study done outside Ethiopia |
| 221 | Khokhar AR, Iqbal T, Hussain M, Rehman QU. Seasonal variation in trachoma prevalence among children, District Dera Ghazi Khan of Punjab, Pakistan. JPMA. The Journal of the Pakistan Medical Association. 2021 Jan 1;71(1 (B)):201-4. | Excluded | the study done outside Ethiopia |
| 222 | Pickering H, Chernet A, Sata E, Zerihun M, Williams CA, Breuer J, Nute AW, Haile M, Zeru T, Tadesse Z, Bailey RL. Genomics of Ocular Chlamydia trachomatis after 5 years of SAFE interventions for trachoma in Amhara, Ethiopia. The Journal of infectious diseases. 2022 Mar 15;225(6):994-1004. | Excluded | Outcome of interest not reported |
| 223 | Adimassu NF, Assem AS, Fekadu SA. Postoperative trachomatous trichiasis: a systematic review and meta-analysis study. International Health. 2023 Nov;15(6):623-9. | Excluded | Outcome of interest not reported |
| 224 | Mwangi, Grace Wangari. "Post-operative Trachomatous Trichiasis in Africa: a systematic review and online survey." (2019). | Excluded | Outcome of interest not reported and the study done outside Ethiopia |
| 225 | WoldeKidan E, Daka D, Legesse D, Laelago T, Betebo B. Prevalence of active trachoma and associated factors among children aged 1 to 9 years in rural communities of Lemo district, southern Ethiopia: community based cross sectional study. BMC infectious diseases. 2019 Dec;19:1-8. | Excluded | Duplicate |
| 226 | Sanders AM, Makoy S, Deathe AR, Ohidor S, Jesudason TC, Nute AW, Odongi P, Boniface L, Abuba S, Delahaut AS, Sebit W. Cost and community acceptability of enhanced antibiotic distribution approaches for trachoma in the Republic of South Sudan: enhancing the A in SAFE (ETAS) study protocol. BMC ophthalmology. 2023 Feb 6;23(1):51. | Excluded | Outcome of interest not reported and the study done outside Ethiopia |
| 227 | Aragie S, Gebresillasie S, Chernet A, Shiferaw A, Tadesse Z, Zerihun M, Varnado NE, Cotter SY, Wittberg DM, Zhou Z, Callahan EK. Community hand-dug wells for trachoma: a cluster-randomized trial. The American Journal of Tropical Medicine and Hygiene. 2021 Feb 1;104(4):1271. | Excluded | Outcome of interest not reported and non observational design |
| 228 | Harding-Esch EM, Holland MJ, Schémann JF, Sissoko M, Sarr B, Butcher RM, Molina-Gonzalez S, Andreasen AA, Mabey DC, Bailey RL. Facial cleanliness indicators by time of day: results of a cross-sectional trachoma prevalence survey in Senegal. Parasites & Vectors. 2020 Dec;13:1-1. | Excluded | Outcome of interest not reported and the study done outside Ethiopia |
| 229 | Abd Elatey SS, El-Megeed A, Abd El-Gawad H, Mohamed AA, El-Mordy A, Abdallah M. Educational Health Program for Mothers regarding Care of Their Preschool Children with Trachoma in Benha City. Benha Journal of Applied Sciences. 2023 Apr 1;8(4):179-89. | Excluded | Outcome of interest not reported and the study done outside Ethiopia |
| 230 | Dulal S, Mishra SK, Taylor HR. Neglected Tropical Diseases and Trachoma. InSouth-East Asia Eye Health: Systems, Practices, and Challenges 2021 Aug 19 (pp. 229-244). Singapore: Springer Singapore. | Excluded | Outcome of interest not reported and the study done outside Ethiopia |
| 231 | Sylla A, Bakayoko S, Lamah PL, Ouendouno A, Sylla A, Diabate RC, Thera JP. Epidemiology of Trachoma in Health District of Fria in 2014. | Excluded | Outcome of interest not reported and the study done outside Ethiopia |
| 232 | \| Gupta N, Vashist P, Senjam SS, Gupta V, Wadhwani M, Manna S, Grover S, Bhardwaj A. Current status of trachoma in India: Results from the national trachoma prevalence survey. Indian Journal of Ophthalmology. 2022 Sep 1;70(9):3260-5. \| \| --- \| | Excluded | Outcome of interest not reported and the study done outside Ethiopia |
| 233 | Harding-Esch EM, Burgert-Brucker CR, Jimenez C, Bakhtiari A, Willis R, Bejiga MD, Mpyet C, Ngondi J, Boyd S, Abdala M, Abdou A. Tropical Data: approach and methodology as applied to trachoma prevalence surveys. Ophthalmic epidemiology. 2023 Nov 2;30(6):544-60. | Excluded | Outcome of interest not reported and the study done outside Ethiopia |
| 234 | Harding-Esch E, Solomon A. Prevalence of trachoma following mass drug administration and community-based surgical service provision in two local government areas of Taraba State, North Eastern Nigeria. Ophthalmic epidemiology. 2023 Mar 30. | Excluded | Outcome of interest not reported and the study done outside Ethiopia |
| 235 | Kahsay BN, Kassa SM, Terefe YA. New intervention model to eliminate the spread of trachoma in hyper-endemic community: Based on a mathematical model. Applied Mathematical Modelling. 2021 Feb 1;90:568-81. | Excluded | Outcome of interest not reported |
| 236 | Jamal M, Kebede F. Exploring multi-level risk factors and post-war burdens of trachomatous trichiasis among displaced population in Raya Kobo districts, implication for urgent action. International Journal of Ophthalmology. 2023 Aug 18;16(8):1299. | Excluded | Outcome of interest not reported |
| 237 | Harding-Esch EM, Holland MJ, Schémann JF, Sillah A, Sarr B, Christerson L, Pickering H, Molina-Gonzalez S, Sarr I, Andreasen AA, Jeffries D. Impact of a single round of mass drug administration with azithromycin on active trachoma and ocular Chlamydia trachomatis prevalence and circulating strains in The Gambia and Senegal. Parasites & vectors. 2019 Dec;12:1-9. | Excluded | Duplicate |
| 238 | Hirpesa, Genet Mulugeta. "Ophthalmology Care in Ethiopia: a Health Economic Evaluation." Master's thesis, The University of Bergen, 2022. | Excluded | the study done outside Ethiopia |
| 239 | Harding-Esch EM, Holland MJ, Schémann JF, Sillah A, Sarr B, Christerson L, Pickering H, Molina-Gonzalez S, Sarr I, Andreasen AA, Jeffries D. Impact of a single round of mass drug administration with azithromycin on active trachoma and ocular Chlamydia trachomatis prevalence and circulating strains in The Gambia and Senegal. Parasites & vectors. 2019 Dec;12:1-9. | Excluded | Outcome of interest not reported and the study done outside Ethiopia |
| 240 | Abebe TA, Tucho GT. The impact of access to water supply and sanitation on the prevalence of active trachoma in Ethiopia: A systematic review and meta-analysis. PLoS Neglected Tropical Diseases. 2021 Sep 9;15(9):e0009644. | Excluded | Outcome of interest not reported |
| 241 | Moyo, George. "Global burden of trichiasis in women as compared to men: Findings from the Global Trachoma Mapping Project." (2019). | Excluded | Outcome of interest not reported |
| 242 | Khan AA, Florea VV, Hussain A, Jadoon Z, Boisson S, Willis R, Dejene M, Bakhtiari A, Mpyet C, Pavluck AL, Gillani M. Prevalence of Trachoma in Pakistan: results of 42 population-based prevalence surveys from the global trachoma mapping project. Ophthalmic epidemiology. 2020 Mar 3;27(2):155-64. | Excluded | the study done outside Ethiopia |
| 243 | Melo JS, Aragie S, Chernet A, Tadesse Z, Dagnew A, Hailu D, Haile M, Zeru T, Wittberg DM, Nash SD, Callahan EK. Targeted antibiotics for trachoma: a cluster-randomized trial. Clinical Infectious Diseases. 2021 Sep 15;73(6):979-86. | Excluded | Outcome of interest not reported |
| 244 | Al-Khatib T, Bella AL, Saboyá-Díaz MI, Solomon AW. Trachoma: The last decade?. Ophthalmic Epidemiology. 2023 Nov 2;30(6):541-3. | Excluded | Outcome of interest not reported and the study done outside Ethiopia |
| 245 | Hassan, Rawan Sharaf Eldein Elamein, Mohamed Abdulmonem Salih Aabdeen, Razan Sharaf Eldein Elamein Hassan, Sagad Omer Obeid Mohamed, Fadwa Mohammed Saad, and Haidar AbuAhmed Mohamed. "Trachoma in Sudan: case series from two eye care hospitals and a review of the literature." (2020). | Excluded | Outcome of interest not reported and the study done outside Ethiopia |
| 246 | Al-Shamahi EY, Al-Shamahi EH, Al-Moyed KA, Al-Shamahy HA, Al-Ankoshy AA. Journal of Ophthalmology Research Reviews & Reports. 2023 | Excluded | Outcome of interest not reported and the study done outside Ethiopia |
| 247 | Wolle MA, Misra N, Naufal F, Saheb Kashaf M, Munoz BE, Mkocha H, Funga N, West SK. The Association Between the Severity of Trachomatous Scarring and Trachomatous Trichiasis Severity in Surgical Patients in Tanzania. Ophthalmic Epidemiology. 2024 Nov 1;31(6):561-7. | Excluded | Outcome of interest not reported and the study done outside Ethiopia |
| 248 | Amoah B, Fronterre C, Johnson O, Dejene M, Seife F, Negussu N, Bakhtiari A, Harding-Esch EM, Giorgi E, Solomon AW, Diggle PJ. Model-based geostatistics enables more precise estimates of neglected tropical-disease prevalence in elimination settings: mapping trachoma prevalence in Ethiopia. International journal of epidemiology. 2022 Apr 1;51(2):468-78. | Excluded | Outcome of interest not reported |
| 249 | Seyum D, Fetene N, Kifle T, Negash H, Kabeto T, Gebre M, Data T, Tadele T, Abayo G, Wondimu A, Butcher R. Prevalence of trachoma from 66 impact surveys in 52 woredas of Southern Nations, Nationalities and Peoples’ and Sidama Regions of Ethiopia, 2017–2019. Ophthalmic epidemiology. 2023 Nov 2;30(6):637-46. | Excluded | Study conducted outside of the eligible period |
| 250 | Nash SD, Astale T, Nute AW, Bethea D, Chernet A, Sata E, Zerihun M, Gessese D, Ayenew G, Ayele Z, Melak B. Population-based prevalence of Chlamydia trachomatis infection and antibodies in four districts with varying levels of trachoma endemicity in Amhara, Ethiopia. The American journal of tropical medicine and hygiene. 2020 Oct 26;104(1):207. | Excluded | Study conducted outside of the eligible period |
| 251 | Gebeyehu G, Mideksa S, Oumer A, Abegaz K. Magnitude of trachoma cases and associated factors among ophthalmic clients attending Menelik II Comprehensive Referral hospital, Addis Ababa, Ethiopia. Ethiopian Journal of Public Health and Nutrition (EJPHN). 2024 Jul 31;7(2):105-12. | Excluded | Study conducted on adult population |
| 252 | Mosenia A, Haile BA, Shiferaw A, Gebresillasie S, Gebre T, Zerihun M, Tadesse Z, Emerson PM, Callahan EK, Zhou Z, Lietman TM. When the Neighboring Village is Not Treated: Role of Geographic Proximity to Communities Not Receiving Mass Antibiotics for Trachoma. Clinical Infectious Diseases. 2023 Mar 15;76(6):1038-42. | Excluded | Outcome of interest not reported |
| 253 | Churko C, Asfaw MA, Zerdo Z. Knowledge, attitude, practices and associated factors towards trachoma among people living in Arba Minch Zuria District, Gamo Zone, Southern Ethiopia. Clinical Ophthalmology. 2021 Jul 16:3075-85. | Excluded | Outcome of interest not reported |
| 254 | Altherr FM, Nute AW, Zerihun M, Sata E, Stewart AE, Gessese D, Melak B, Astale T, Ayenew G, Callahan EK, Chanyalew M. Associations between Water, Sanitation and Hygiene (WASH) and trachoma clustering at aggregate spatial scales, Amhara, Ethiopia. Parasites & vectors. 2019 Dec;12:1-1. | Excluded | Study conducted outside of the eligible period |
| 255 | Tedijanto C, Aragie S, Gwyn S, Wittberg DM, Zeru T, Tadesse Z, Chernet A, Thompson IJ, Nash SD, Lietman TM, Martin DL. Seroreversion to Chlamydia trachomatis Pgp3 antigen among children in a hyperendemic region of Amhara, Ethiopia. The Journal of Infectious Diseases. 2024 Aug 15;230(2):293-7. | Excluded | Outcome of interest not reported |
| 256 | Asmare ZA, Assefa NL, Abebe D, Nigatu SG, Alimaw YA. Trachoma prevention practice and associated factors among mothers having children aged under nine years in Andabet district, northwest Ethiopia, 2022: A multi-level analysis. PLOS Neglected Tropical Diseases. 2023 Jun 30;17(6):e0011433. | Excluded | Outcome of interest not reported |
| 257 | Lakew S, Asefa G, Zerdo Z. Assessment of the status of improved F&E trachoma control practices among children of agro-pastoralists in Southern Ethiopia: a mixed design survey using theory of triadic influences. BMC Public Health. 2023 Mar 23;23(1):556. | Excluded | Outcome of interest not reported |
| 258 | Basha GW, Woya AA, Tekile AK. Prevalence and risk factors of active trachoma among primary school children of Amhara Region, Northwest Ethiopia. Indian Journal of Ophthalmology. 2020 May 1;68(5):750-4. | Excluded | Study conducted outside of the eligible period |
| 259 | Reda G, Yemane D, Gebreyesus A. Prevalence and associated factors of active trachoma among 1–9 years old children in Deguatemben, Tigray, Ethiopia, 2018: community cross-sectional study. BMC ophthalmology. 2020 Dec;20:1-9. | Excluded | Study conducted outside of the eligible period |
| 260 | Kassaw MW, Abebe AM, Tegegne KD, Getu MA, Bihonegn WT. Prevalence and associations of active trachoma among rural preschool children in Wadla district, northern Ethiopia. BMC ophthalmology. 2020 Dec;20:1-0. | Excluded | Study conducted outside of the eligible period |
| 261 | Lorato MM. Prevalence of Active Trachoma among Primary School Children in Yigalem Town, Ethiopia. EC Ophthalmology. 2021;12:23-9. | Excluded | Study conducted outside of the eligible period |
| 262 | Stewart AE, Zerihun M, Gessese D, Melak B, Sata E, Nute AW, Astale T, Endeshaw T, Teferi T, Tadesse Z, Callahan EK. Progress to eliminate trachoma as a public health problem in Amhara National Regional State, Ethiopia: results of 152 population-based surveys. The American journal of tropical medicine and hygiene. 2019 Sep 23;101(6):1286. | Excluded | Study conducted outside of the eligible period |
| 263 | Alambo MM, Lake EA, Bitew Workie S, Wassie AY. Prevalence of active trachoma and associated factors in Areka Town, south Ethiopia, 2018. Interdisciplinary Perspectives on Infectious Diseases. 2020;2020(1):8635191. | Excluded | Study conducted outside of the eligible period |
| 264 | Adane B, Malede A, Sewunet B, Kumlachew L, Moges M, Woretaw L, Temesgen T, Bewket Y, Gete M, Yirdaw G, Ayele A. Determinants of Trachomatous Inflammation-Follicular Among Children Aged 1 to 9 Years Old in a Rural Area of Gozamn District, Northwestern Ethiopia: A Matched Case-Control Study. Environmental Health Insights. 2023 Apr;17:11786302231169941. | Excluded | Study conducted outside of the eligible period |
| 265 | Seyum D, Fetene N, Kifle T, Negash H, Kabeto T, Gebre M, Data T, Tadele T, Abayo G, Wondimu A, Butcher R. Prevalence of trachoma from 66 impact surveys in 52 woredas of Southern Nations, Nationalities and Peoples’ and Sidama Regions of Ethiopia, 2017–2019. Ophthalmic epidemiology. 2023 Nov 2;30(6):637-46. | Excluded | Study conducted outside of the eligible period |
| 266 | Miecha H, Dejene M, Adugna D, Kebede A, Yadeta D, Alemayehu A, Abateneh A, Wondimu A, Dayessa M, Shafi M, Taye E. Prevalence of trachoma after implementation of trachoma elimination interventions in Oromia regional State, Ethiopia: Results of impact surveys in 131 evaluation units covering 139 districts. Ophthalmic epidemiology. 2023 Nov 2;30(6):647-54. | Excluded | Study conducted outside of the eligible period |
| 267 | Tedijanto, Christine, et al. "Predicting future community-level ocular Chlamydia trachomatis infection prevalence using serological, clinical, molecular, and geospatial data." PLoS neglected tropical diseases 16.3 (2022): e0010273. | Excluded | Duplicate |
| 268 | Ayelgn K, Guadu T, Getachew A. Low prevalence of active trachoma and associated factors among children aged 1–9 years in rural communities of Metema District, Northwest Ethiopia: a community based cross-sectional study. Italian Journal of Pediatrics. 2021 May 17;47(1):114. | Excluded | Study conducted outside of the eligible period |
| 269 | Tsegay T, Mengistu Y, Nigussie T. Application of ordinal logistic regression analysis in determining risk factors of active trachoma among rural children of aged 1–9 years old in Kaffa Zone, Southwest Ethiopia. ARC J Public Health Community Med. 2019;4(1):20-8. | Excluded | Study conducted outside of the eligible period |
| 270 | Nash SD, Chernet A, Moncada J, Stewart AE, Astale T, Sata E, Zerihun M, Gessese D, Melak B, Ayenew G, Ayele Z. Ocular Chlamydia trachomatis infection and infectious load among pre-school aged children within trachoma hyperendemic districts receiving the SAFE strategy, Amhara region, Ethiopia. PLoS neglected tropical diseases. 2020 May 18;14(5):e0008226. | Excluded | Outcome of interest not reported |
| 271 | Abebe AM, Tegegne KD, Getu MA, Bihonegn WT. Prevalence and Risk Factors of Active Trachoma among Rural Preschool Children in Wadla District, Northern Ethiopia: A Community Based Cross-Sectional Study. | Excluded | Study conducted outside of the eligible period |
| 272 | Reda G, Yemane D, Gebreyesus A. Prevalence and associated factors of active trachoma among 1–9 years old children in Deguatemben, Tigray, Ethiopia, 2018: community cross-sectional study. BMC ophthalmology. 2020 Dec;20:1-9. | Excluded | Study conducted outside of the eligible period |
| 273 | Miecha H, Dejene M, Adugna D, Kebede A, Yadeta D, Alemayehu A, Abateneh A, Dayessa M, Shafi M, Taye E, Balcha L. Prevalence of Trachoma in Pre-validation Surveillance Surveys in 11 Evaluation Units (Covering 12 Districts) in Oromia Regional State, Ethiopia: Results from 2018− 2020. Ophthalmic epidemiology. 2023 Nov 2;30(6):655-62. | Excluded | Study conducted outside of the eligible period |
| 274 | Nash SD, Chernet A, Weiss P, Nute AW, Zerihun M, Sata E, Gessese D, Jensen KA, Ayele Z, Melak B, Zeru T. Prevalence of ocular Chlamydia trachomatis infection in Amhara region, Ethiopia, after 8 years of trachoma control interventions. The American Journal of Tropical Medicine and Hygiene. 2023 Jan 9;108(2):261. | Excluded | Study conducted outside of the eligible period |
| 275 | WoldeKidan E, Daka D, Legesse D, Laelago T, Betebo B. Prevalence of active trachoma and associated factors among children aged 1 to 9 years in rural communities of Lemo district, southern Ethiopia: community based cross sectional study. BMC infectious diseases. 2019 Dec;19:1-8. | Excluded | Study conducted outside of the eligible period |
| 276 | Asfaw M, Zolfo M, Negussu N, Tadesse F, Tadele T, Sisay A, Seyum D, Gezmu T, Senkoro M, Owiti P, Adriaensen W. Towards the trachoma elimination target in the Southern region of Ethiopia: How well is the SAFE strategy being implemented?. The Journal of Infection in Developing Countries. 2020 Jun 30;14(06.1):3S-9S. | Excluded | Outcome of interest not reported |
| 277 | Ashine BN, Tesfahun E, Sigate SG, Gebreegziabher ZA, Mekuria AD. Clinically active trachoma and its associated factors among one to nine year old children in Tarmaber district, Amhara region, Ethiopia: community based crossectional study design. | Excluded | Study conducted outside of the eligible period |
| 278 | Atsbha SG. A review of the prevalence of trachoma, its control program and challenges in Ethiopia. International Journal of Drug Regulatory Affairs. 2023;11(1):54-60. | Excluded | Outcome of interest not reported |
| 279 | Meron Y, Mandefro S, Haymanot M. Active Trachoma and Associated Factors among Children (Aged 1-9 years) in Haramaya District, Eastern Ethiopia. East African Journal of Health and Biomedical Sciences. 2022 May 25;6(1):1-0. | Excluded | Study conducted outside of the eligible period |
| 280 | Khan AA, Florea VV, Hussain A, Jadoon Z, Boisson S, Willis R, Dejene M, Bakhtiari A, Mpyet C, Pavluck AL, Gillani M. Prevalence of Trachoma in Pakistan: results of 42 population-based prevalence surveys from the global trachoma mapping project. Ophthalmic epidemiology. 2020 Mar 3;27(2):155-64. | Excluded | study done outside Ethiopia |
| 281 | Aragie S, Tadesse W, Dagnew A, Hailu D, Dubie M, Wittberg DM, Melo JS, Haile M, Zeru T, Freeman MC, Nash SD. Changing hygiene behaviours: a cluster-randomized trial, Ethiopia. Bulletin of the World Health Organization. 2021 Aug 30;99(11):762. | Excluded | Outcome of interest not reported |
| 282 | Flueckiger RM, Courtright P, Abdala M, Abdou A, Abdulnafea Z, Al-Khatib TK, Amer K, Amiel ON, Awoussi S, Bakhtiari A, Batcho W. The global burden of trichiasis in 2016. PLoS neglected tropical diseases. 2019 Nov 25;13(11):e0007835. | Excluded | Duplicate |
| 283 | Pickering H, Chernet A, Sata E, Zerihun M, Williams CA, Breuer J, Nute AW, Haile M, Zeru T, Tadesse Z, Bailey RL. Genomics of Ocular Chlamydia trachomatis after 5 years of SAFE interventions for trachoma in Amhara, Ethiopia. The Journal of infectious diseases. 2022 Mar 15;225(6):994-1004. | Excluded | Outcome of interest not reported |
| 284 | Chweya RN, Onyango CA, Saigilu S, Mwangi C, Gachohi JM. Spatial and network mapping of comorbidity with trachoma and visual-impairing NCDs in a pastoralist community in Kenya: implications for SDGs and UHC. International Health. 2024 Jan;16(1):35-44. | Excluded | Outcome of interest not reported and the study done outside Ethiopia |
| 285 | Alemayehu W, Cherinet A. Eye diseases and Blindness. InThe Ecology Of Health And Disease In Ethiopia 2019 Jul 11 (pp. 237-250). Routledge. | Excluded | Outcome of interest not reported |
| 286 | Shifarew H, Negash L. Risk Factors for Blindness and Partial Blindness in the Amhara Region of Ethiopia: Spatial and Regression Analyses. Med Discoveries. 2023;2(9):1071. | Excluded | Outcome of interest not reported |
| 287 | West SK. Milestones in the fight to eliminate trachoma. Ophthalmic and Physiological Optics. 2020 Mar;40(2):66-74. | Excluded | Outcome of interest not reported |
| 289 | Nesemann JM, Seider MI, Snyder BM, Maamari RN, Fletcher DA, Haile BA, Tadesse Z, Varnado NE, Cotter SY, Callahan EK, Emerson PM. Comparison of smartphone photography, single-lens reflex photography, and field-grading for trachoma. The American journal of tropical medicine and hygiene. 2020 Oct 5;103(6):2488. | Excluded | Outcome of interest not reported |
| 290 | Gebeyehu G, Mideksa S, Oumer A, Abegaz K. Magnitude of trachoma cases and associated factors among ophthalmic clients attending Menelik II Comprehensive Referral hospital, Addis Ababa, Ethiopia. Ethiopian Journal of Public Health and Nutrition (EJPHN). 2024 Jul 31;7(2):105-12. | Excluded | Conducted among adult population |
| 291 | Abdul NA. *Molecular Detection and Characterizations of Chlamydia trachomatis Ocular Infection in Al-Gadarif State–Sudan* (Doctoral dissertation, Al-Neelain University). | Excluded | Outcome of interest not reported |
| 292 | YENEGETA B. *Detection and Grading of Trachoma Using Deep Convolutional Neural Network* (Doctoral dissertation | Excluded | Outcome of interest not reported |
| 293 | Anley DT, Anteneh RM, Tegegne YS, Ferede OL, Zemene MA, Angaw DA, Teym A. Prevalence of visual impairment and associated factors among children in Ethiopia: Systematic review and meta-analysis. PloS one. 2022 Jul 21;17(7):e0271433. | Excluded | Outcome of interest not reported |
| 294 | Al-Shamahi EY, Al-Shamahi EH, Al-Moyed KA, Al-Shamahy HA, Al-Ankoshy AA. Journal of Ophthalmology Research Reviews & Reports. | Excluded | Outcome of interest not reported |
| 295 | Wazir JF, Rana S, Javaid N, Nargus S. Distribution Pattern of Trachoma in Pakistan and Monitoring the Effects of Water Availability upon Disease prevalence-case study of Khyber Pakhtunkhwa. Pakistan Journal of Medical & Health Sciences. 2023 Jun 16;17(06):41-. | Excluded | Outcome of interest not reported and the study done outside Ethiopia |
| 296 | Porth JM, Deiotte E, Dunn M, Bashshur R. A review of the literature on the global epidemiology of corneal blindness. Cornea. 2019 Dec 1;38(12):1602-9. | Excluded | Duplicate |
| 297 | Wazir JF, Nargus S, Rana S. Distribution Pattern of Trachoma in Pakistan and Monitoring the Effects of Water Availability upon Disease prevalence. Pakistan Journal of Medical & Health Sciences. 2022 Dec 11;16(11):66-. | Excluded | Outcome of interest not reported and the study done outside Ethiopia |
| 298 | Naqvi FA, Das JK, Salam RA, Raza SF, Lassi ZS, Bhutta ZA. Interventions for neglected tropical diseases among children and adolescents: a meta-analysis. Pediatrics. 2022 May 1;149(Supplement 6). | Excluded | Non-observational study design |
| 299 | Blumberg S, Borlase A, Prada JM, Solomon AW, Emerson P, Hooper PJ, Deiner MS, Amoah B, Hollingsworth TD, Porco TC, Lietman TM. Implications of the COVID-19 pandemic in eliminating trachoma as a public health problem. Transactions of the Royal Society of Tropical Medicine and Hygiene. 2021 Mar;115(3):222-8. | Excluded | Outcome of interest not reported |
| 300 | Meneghim RL, Madeira NG, Ribolla PE, Padovani CR, Schellini SA. Flies as possible vectors of inflammatory trachoma transmission in a Brazilian municipality. Revista do Instituto de Medicina Tropical de São Paulo. 2021 Sep 3;63:e66. | Excluded | Outcome of interest not reported and the study done outside Ethiopia |
| 301 | Gupta N, Vashist P, Senjam SS, Gupta V, Wadhwani M, Manna S, Grover S, Bhardwaj A. Current status of trachoma in India: Results from the national trachoma prevalence survey. Indian Journal of Ophthalmology. 2022 Sep 1;70(9):3260-5. | Excluded | the study done outside Ethiopia |
| 302 | Hassan, Rawan Sharaf Eldein Elamein, Mohamed Abdulmonem Salih Aabdeen, Razan Sharaf Eldein Elamein Hassan, Sagad Omer Obeid Mohamed, Fadwa Mohammed Saad, and Haidar AbuAhmed Mohamed. "Trachoma in Sudan: case series from two eye care hospitals and a review of the literature." (2020). | Excluded | Outcome of interest not reported and the study done outside Ethiopia |
| 303 | Al-Shamahi EY, Al-Shamahi EH, Al-Moyed KA, Al-Shamahy HA, Al-Ankoshy AA. Journal of Ophthalmology Research Reviews & Reports. 2023 | Excluded | Outcome of interest not reported |
| 304 | Wolle MA, Misra N, Naufal F, Saheb Kashaf M, Munoz BE, Mkocha H, Funga N, West SK. The Association Between the Severity of Trachomatous Scarring and Trachomatous Trichiasis Severity in Surgical Patients in Tanzania. Ophthalmic Epidemiology. 2024 Nov 1;31(6):561-7. | Excluded | Outcome of interest not reported and the study done outside Ethiopia |
| 305 | Amoah B, Fronterre C, Johnson O, Dejene M, Seife F, Negussu N, Bakhtiari A, Harding-Esch EM, Giorgi E, Solomon AW, Diggle PJ. Model-based geostatistics enables more precise estimates of neglected tropical-disease prevalence in elimination settings: mapping trachoma prevalence in Ethiopia. International journal of epidemiology. 2022 Apr 1;51(2):468-78. | Excluded | Outcome of interest not reported |
| 306 | Seyum D, Fetene N, Kifle T, Negash H, Kabeto T, Gebre M, Data T, Tadele T, Abayo G, Wondimu A, Butcher R. Prevalence of trachoma from 66 impact surveys in 52 woredas of Southern Nations, Nationalities and Peoples’ and Sidama Regions of Ethiopia, 2017–2019. Ophthalmic epidemiology. 2023 Nov 2;30(6):637-46. | Excluded | Study conducted outside of the eligible period |
| 307 | Hassan, Rawan Sharaf Eldein Elamein, Mohamed Abdulmonem Salih Aabdeen, Razan Sharaf Eldein Elamein Hassan, Sagad Omer Obeid Mohamed, Fadwa Mohammed Saad, and Haidar AbuAhmed Mohamed. "Trachoma in Sudan: case series from two eye care hospitals and a review of the literature." (2020). | Excluded | Duplicate |
| 308 | Markos M, Kefyalew B, Tesfaye HB. Pooled prevalence of blindness in Ethiopia: a systematic review and meta-analysis. BMJ Open Ophthalmology. 2022 Jun 6;7(1). | Excluded | Outcome of interest not reported |
| 309 | Grygiel-Górniak B, Folga BA. Chlamydia trachomatis—An Emerging Old Entity?. Microorganisms. 2023 May 14;11(5):1283. | Excluded | Outcome of interest not reported |
| 310 | TRACHOME IE, LA RÉGION ED, DU CAMEROUN NO, LES CAS DES DISTRICTS DP, REY TE. Kah Evans NGHA. Revue Internationale Dônni. 2023 Dec;3(2). | Excluded | Outcome of interest not reported |
| 311 | Borlase A, Prada JM, Crellen T. Modelling morbidity for neglected tropical diseases: the long and winding road from cumulative exposure to long-term pathology. Philosophical Transactions of the Royal Society B. 2023 Oct 9;378(1887):20220279. | Excluded | Outcome of interest not reported |
| 312 | Mtuy TB, Bardosh K, Ngondi J, Mwingira U, Seeley J, Burton M, Lees S. Understanding hard-to-reach communities: local perspectives and experiences of trachoma control among the pastoralist Maasai in northern Tanzania. Journal of Biosocial Science. 2021 Nov;53(6):819-38. | Excluded | Outcome of interest not reported and the study done outside Ethiopia |
| 313 | Kelly-Hope LA, Sanders AM, Harding-Esch E, Willems J, Ahmed F, Vincer F, Hill R. Complex emergencies and the control and elimination of neglected tropical diseases in Africa: developing a practical approach for implementing safe and effective mapping and intervention strategies. Conflict and Health. 2021 Dec;15:1-2. | Excluded | Outcome of interest not reported |
| 314 | Mtuy T. *Maasai Response to Mass Drug Administration for Trachoma in a Changing Political Economy in Tanzania* (Doctoral dissertation, London School of Hygiene & Tropical Medicine). | Excluded | Outcome of interest not reported and the study done outside Ethiopia |
| 315 | Chen X, Munoz B, Mkocha H, Gaydos CA, Dize L, Quinn TC, West SK. Risk of seroconversion and seroreversion of antibodies to Chlamydia trachomatis pgp3 in a longitudinal cohort of children in a low trachoma prevalence district in Tanzania. PLoS neglected tropical diseases. 2022 Jul 13;16(7):e0010629. | Excluded | Outcome of interest not reported and the study done outside Ethiopia |
| 316 | Tedijanto C, Solomon AW, Martin DL, Nash SD, Keenan JD, Lietman TM, Lammie PJ, Aiemjoy K, Amza A, Aragie S, Arzika AM. Monitoring transmission intensity of trachoma with serology. Nature communications. 2023 Jun 5;14(1):3269. | Excluded | Outcome of interest not reported |
| 317 | Blindness EF, Filariasis L. Disease Elimination—Successes and Challenges. The Principles and Practice of Disease Eradication. 2024:141. | Excluded | Outcome of interest not reported |
| 318 | Bhat A, Jhanji V. Bacterial conjunctivitis. Infections of the Cornea and Conjunctiva. 2021:1-6. | Excluded | Outcome of interest not reported |
| 319 | Brady CJ, Cockrell RC, Aldrich LR, Wolle MA, West SK. A Virtual Reading Center Model Using Crowdsourcing to Grade Photographs for Trachoma: Validation Study. Journal of medical Internet research. 2023 Apr 6;25:e41233. | Excluded | Outcome of interest not reported |
| 320 | Makonnen E, Gelibo T, Agedew E, Bekele A, Misker D, Worku S, Tadele A, Mekonnen Y, Belay A, Challa F, Awoke T. Patterns of biochemical markers, metabolic syndrome and their predictors among adult population in Moringa stenopetala consuming and non-consuming areas in Southern Ethiopia: A comparative cross-sectional study. Ethiopian Journal of Public Health and Nutrition (EJPHN). 2023 Jul 31;6(2):94-106. | Excluded | Outcome of interest not reported |
| 321 | Ghasemian E, Ramadhani A, Harte A, Mafuru E, Derrick T, Mtuy T, Massae P, Malissa A, Breuer J, Pickering H, Bailey RL. Evolutionary Dynamics in the Genome of Ocular Chlamydia trachomatis Strains from Northern Tanzania, pre-and post-Mass Drug Administration. | Excluded | Outcome of interest not reported and the study done outside Ethiopia |
| 322 | Ezediuno LO, Onile OS, Oladipo EK, Majolagbe ON, Jimah EM, Senbadejo TY. Informatics in Medicine Unlocked. | Excluded | Outcome of interest not reported |
| 323 | Memirie ST, Demeshko A, Habtemichael M, Mesele T, Haileselassie A, Baker P, NORHEIM O, Drake T. New Compact for Financing Health Services in Ethiopia. Center for Global Development; 2024 Sep 11. | Excluded | Outcome of interest not reported |
| 324 | Melaku T, Gashaw M, Chelkeba L, Berhane M, Bekele S, Lemi G, Wakjira T, Tesfaw G, Mekonnen Z, Ali S, Kroidl A. Evaluation of adult outpatient antibiotics use at Jimma Medical Center (With defined daily doses for usage metrics). Infection and Drug Resistance. 2021 Apr 28:1649-58. | Excluded | Outcome of interest not reported |
| 325 | Khan SA, Nabeel K, Muhammad I, Batool S, Javed S. Awareness of parents regarding eye diseases and eye care needs among children of Tehsil Babuzai, District Swat. Pakistan Journal of Ophthalmology. 2023 Jun 30;39(3). | Excluded | Outcome of interest not reported and the study done outside Ethiopia |
| 326 | Konishi T, Sonoda K, Hayashi K, PENG Y, Yamauchi T. Sanitation facilities, water quality, and child health in a hunter-gatherer, semi-sedentary village in Cameroon. Sanitation Value Chain. 2022;6(1):23-38. | Excluded | Outcome of interest not reported and the study done outside Ethiopia |
| 327 | Ayelgn, Kessete, Tadesse Guadu, and Atalay Getachew. "Low prevalence of active trachoma and associated factors among children aged 1–9 years in rural communities of Metema District, Northwest Ethiopia: a community based cross-sectional study." *Italian Journal of Pediatrics* 47.1 (2021): 114. | Excluded | Duplicate |
| 328 | Ayalew AA. Effects of undernutrition on treatment outcomes among adults living with HIV in Northwest Ethiopia: A longitudinal study. University of Technology Sydney (Australia); 2023. | Excluded | Outcome of interest not reported |
| 329 | Tedijanto, Christine, et al. "Predicting future community-level ocular Chlamydia trachomatis infection prevalence using serological, clinical, molecular, and geospatial data." PLoS neglected tropical diseases 16.3 (2022): e0010273. | Excluded | Duplicate |
| 330 | Oyeyemi OT, Ogundahunsi O, Schunk M, Fatem RG, Shollenberger LM. Neglected tropical disease (NTD) diagnostics: current development and operations to advance control. Pathogens and Global Health. 2024 Jan 2;118(1):1-24. | Excluded | Outcome of interest not reported |
| 331 | Labrea VN, Raupp IT, Fragomeni FW, Pieta MP, Santoro LL, De Maman RS, da Silveira BL. Avoidable blindness: a review on its leading causes worldwide Cegueira evitável: uma revisão sobre suas principais causas no mundo. Brazilian Journal of Health Review. 2022 Mar;5(2):6332-45. | Excluded | Outcome of interest not reported and the study done outside Ethiopia |
| 332 | Tapia-Barredo L, Delgadillo MA, Paulino-Ramírez R. Geospatial distribution of Chikungunya and Zika virus outbreaks in the Dominican Republic: two sides of the same coin?. Transactions of the Royal Society of Tropical Medicine and Hygiene. 2019. | Excluded | Outcome of interest not reported and the study done outside Ethiopia |
| 333 | Kanda A. *Framework for Selection and Use of Appropriate Rural Sanitation Technologies in Low-Income settingsIngs* (Doctoral dissertation, University of Pretoria (South Africa)). | Excluded | Outcome of interest not reported and the study done outside Ethiopia |
| 334 | Favacho JD, Leite KK, Jacomasso T, Farias AB, Franco Filho LC, Gomes ST, Dos Reis HS, Mota GD, Schluga PH, Tassi WS, Rampazzo RD. Validation of a New Duplex Real-Time Polymerase Chain Reaction for Chlamydia trachomatis DNA Detection in Ocular Swab Samples. Diagnostics. 2024 Apr 25;14(9):892. | Excluded | Outcome of interest not reported and the study done outside Ethiopia |
| 335 | McPherson S, Endamalaw G, Balche TT, Mabey D, Marks M, McCleod D, Solomon A, Kebede B, Negessu N, Deribe K, Aseffa A. Study Proposal. | Excluded | Outcome of interest not reported |
| 336 | Lopes MJ, da Silva ET, Ca J, Gonçalves A, Rodrigues A, Mandjuba C, Nakutum J, D’Alessandro U, Achan J, Logan J, Bailey R. Perceptions, attitudes and practices towards scabies in communities on the Bijagós Islands, Guinea-Bissau. Transactions of The Royal Society of Tropical Medicine and Hygiene. 2020 Jan 6;114(1):49-56. | Excluded | Outcome of interest not reported and the study done outside Ethiopia |
| 337 | Caruso BA, Sclar GD, Routray P, Majorin F, Nagel C, Clasen T. A cluster-randomized multi-level intervention to increase latrine use and safe disposal of child feces in rural Odisha, India: the Sundara Grama research protocol. BMC Public Health. 2019 Dec;19:1-9. | Excluded | Outcome of interest not reported and the study done outside Ethiopia |
| 338 | Amanuel K, Maleda T, Ermias S, Shiferaw L. Caring Behavior and Associated Factors among Nurses Working in Selected Public Hospitals in Southern Ethiopia. East African Journal of Health and Biomedical Sciences. 2023;7(2):1-0. | Excluded | Outcome of interest not reported |
| 339 | Birhan M, Syoum A, Ibrahim SM, Fentahun T, Mohammed A, Berhane N, Bitew M, Gelaye E, Atanaw MB, Getachew B, Dessalegn B. Research Article Serological Evidence of Infectious Laryngotracheitis Infection and Associated Risk Factors in Chickens in Northwestern Ethiopia. | Excluded | Outcome of interest not reported |
| 340 | de Brito CM, Barbosa CC, de Andrade SM, de Oliveira AL, Montarroyos UR, Ferraz C, de Toledo Vieira M, Lopes MD, Gouveia GC, de Medeiros ZM. Household survey of trachoma among children living in Pernambuco, Brazil. Pathogens. 2019 Nov 25;8(4):263. | Excluded | Duplicate |
| 341 | Moncada S, Bambrick H, Briguglio M. The health impacts of a community biogas facility in an informal Urban settlement: does training matter?. Journal of Development Effectiveness. 2019 Apr 3;11(2):189-202. | Excluded | Outcome of interest not reported |
| 342 | Delgadillo MA, Tapia-Barredo L, Paulino-Ramírez R. Schistosomiasis mansoni in La Hispaniola: revisiting a neglected tropical disease in the Caribbean. Transactions of the Royal Society of Tropical Medicine and Hygiene. 2019. | Excluded | Outcome of interest not reported and the study done outside Ethiopia |
| 343 | Hamelmal A, Sisay H, Nesredin A, Shiferaw L. Barriers to Implementation of Nursing Rounds among Nurses Working in Public Hospitals in Harar, Eastern Ethiopia: A Qualitative Descriptive Study. East African Journal of Health and Biomedical Sciences. 2024 Jun 28;8(1):17-26. | Excluded | Outcome of interest not reported |
| 344 | Hart JD, Kalua K, Keenan JD, Lietman TM, Bailey RL. Effect of mass treatment with azithromycin on causes of death in children in Malawi: secondary analysis from the MORDOR trial. The American journal of tropical medicine and hygiene. 2020 Apr 27;103(3):1319. | Excluded | Outcome of interest not reported and the study done outside Ethiopia |
| 345 | Faramand TH, Dale K, Ivankovich M, Roberts K, Hall ML, Foster AA. Desk Review on Gender Issues Affecting Neglected Tropical Diseases. | Excluded | Outcome of interest not reported |
| 346 | Delea MG. *Social constructs, behaviour change, and the uptake of community-based WASH interventions: metrics and analytical approaches for measuring collective efficacy* (Doctoral dissertation, London School of Hygiene & Tropical Medicine). | Excluded | Outcome of interest not reported |
| 347 | Fong IW, Fong IW. Mass Drug Treatment of Tropical Diseases: Is It Really Progress?. Current Trends and Concerns in Infectious Diseases. 2020:217-39. | Excluded | Outcome of interest not reported and the study done outside Ethiopia |
| 348 | Mazalo JV. Fatores de risco associados a ocorrência de tracoma em comunidade assistida pelo programa" Um estudante uma família" da Universidade Lúrio, Moçambique. Saúde e meio ambiente: revista interdisciplinar. 2021 Jun 29;10:158-69. | Excluded | Outcome of interest not reported and the study done outside Ethiopia |
| 349 | Shafi Abdurahman O, Last A, Macleod D, Habtamu E, Versteeg B, Dumessa G, Guye M, Nure R, Adugna D, Miecha H, Greenland K. Trachoma risk factors in Oromia Region, Ethiopia. PLoS neglected tropical diseases. 2023 Nov 7;17(11):e0011679. | Excluded | Duplicate |
| 350 | Ayelgn, Kessete, Tadesse Guadu, and Atalay Getachew. "Low prevalence of active trachoma and associated factors among children aged 1–9 years in rural communities of Metema District, Northwest Ethiopia: a community based cross-sectional study." *Italian Journal of Pediatrics* 47.1 (2021): 114. | Excluded | Duplciate |
| 351 | Tedijanto C, Solomon AW, Martin DL, Nash SD, Keenan JD, Lietman TM, Lammie PJ, Aiemjoy K, Amza A, Aragie S, Arzika AM. Monitoring transmission intensity of trachoma with serology. Nature communications. 2023 Jun 5;14(1):3269. | Excluded | Duplicate |
| 352 | Prevalence and Factors Associated with Active Trachoma among Children 1-9 years of Age in the Catchment Population of Tora Primary Hospital, Silte zone, Southern Ethiopia, 2020 | Excluded | Duplicate |
| 353 | Cassivi A, Guilherme S, Bain R, Tilley E, Waygood EO, Dorea C. Drinking water accessibility and quantity in low and middle-income countries: A systematic review. International Journal of Hygiene and Environmental Health. 2019 Aug 1;222(7):1011-20. | Excluded | Outcome of interest not reported and the study done outside Ethiopia |
| 354 | Howard G, Bartram J, Williams A, Overbo A, Geere JA. Domestic water quantity, service level and health. | Excluded | Outcome of interest not reported and the study done outside Ethiopia |
| 355 | Basha GW, Woya AA, Tekile AK. Prevalence and risk factors of active trachoma among primary school children of Amhara Region, Northwest Ethiopia. Indian Journal of Ophthalmology. 2020 May 1;68(5):750-4. | Excluded | Duplicate |
| 356 | Shafi Abdurahman O, Last A, Macleod D, Habtamu E, Versteeg B, Dumessa G, Guye M, Nure R, Adugna D, Miecha H, Greenland K. Trachoma risk factors in Oromia Region, Ethiopia. PLoS neglected tropical diseases. 2023 Nov 7;17(11):e0011679. | Exlcuded | Duplicate |
| 357 | Hart JD, Kalua K, Keenan JD, Lietman TM, Bailey RL. Cost-effectiveness of mass treatment with azithromycin for reducing child mortality in Malawi: secondary analysis from the MORDOR trial. The American journal of tropical medicine and hygiene. 2020 Apr 27;103(3):1283. | Excluded | Outcome of interest not reported and the study done outside Ethiopia |
| 358 | Agler E, Crigler M. Under the Big Tree: Extraordinary Stories from the Movement to End Neglected Tropical Diseases. JHU Press; 2019 Jan 15. | Excluded | Outcome of interest not reported |
| 359 | Marks M, Sammut T, Cabral MG, Teixeira da Silva E, Goncalves A, Rodrigues A, Manjuba C, Nakutum J, Ca J, D’Alessandro U, Achan J. The prevalence of scabies, pyoderma and other communicable dermatoses in the Bijagos Archipelago, Guinea-Bissau. PLoS neglected tropical diseases. 2019 Nov 18;13(11):e0007820. | Excluded | Outcome of interest not reported and the study done outside Ethiopia |
| 360 | Ocular Chlamydia trachomatis infection and infectious load among pre-school aged children within trachoma hyperendemic districts receiving the SAFE strategy, Amhara region, Ethiopia | Excluded | Duplicate |
| 361 | Ellalie KC, Masséssé SO, Koffi BP, Siméon KA, Kouakou AA, François-Xavier KO. Prévalence du trachome dans le district sanitaire de Danané (Côte d’Ivoire)/Prevalence of trachoma in the Danane Health District (Côte d’Ivoire). | Excluded | the study done outside Ethiopia |
| 362 | Nji E, Traore DA, Ndi M, Joko CA, Doyle DA. BioStruct-Africa: empowering Africa-based scientists through structural biology knowledge transfer and mentoring–recent advances and future perspectives. Synchrotron Radiation. 2019 Sep 1;26(5):1843-50. | Excluded | Outcome of interest not reported |
| 363 | Ssemanda I, Banda J, Jacobs C, Tukei O. INNOVATIVE RESEARCH AND KNOWLEDGE. | Excluded | Outcome of interest not reported |
| 364 | Tapia-Barredo L, Paulino-Ramírez R. Clinical and demographic characteristics of malaria infections in the Dominican Republic. Transactions of the Royal Society of Tropical Medicine and Hygiene. 2019. | Excluded | Outcome of interest not reported and the study done outside Ethiopia |
| 365 | Woreta M, Zewudie K, Gebremariam A, Freeman MC. Maryann G. Delea1, Jedidiah S. Snyder1, Mulusew Belew2, Bethany A. Caruso1, Joshua V. Garn3, Gloria D. Sclar1. | Excluded | Outcome of interest not reported |
| 366 | Abeje BT, Salau AO, Belay G, Chhabra G, Kaushik K, Braide SL. Braille Recognition of Geez Numbers Using an Optimal Deep Learning Algorithm. In2024 2nd International Conference on Advancement in Computation & Computer Technologies (InCACCT) 2024 May 2 (pp. 198-203). IEEE. | Excluded | Outcome of interest not reported |
| 367 | Aquino-Canchari CR, Chavez-Bustamante SG. The 100 most cited articles on trachoma: a bibliometric analysis. International Ophthalmology. 2023 Nov;43(11):4235-46. | Excluded | Citation |
| 368 | Yenegeta B, Assabie Y. TrachomaNet: Detection and grading of trachoma using texture feature based deep convolutional neural network. Multimedia Tools and Applications. 2023 Jan;82(3):4209-34. | Excluded | Outcome of interest not reported |
| 369 | Aquino-Canchari CR, Chavez-Bustamante SG, Zarate-Chavez ML. Scientific production of trachoma worldwide: a bibliometric analysis during the period 1886 to 2022/Producción científica de tracoma a nivel mundial: un análisis bibliométrico durante el periodo 1886 a 2022. InAnales de la facultad de medicina 2023 Dec 22 (Vol. 84, No. 4, pp. NA-NA). Universidad Nacional Mayor de San Marcos. | Excluded | Outcome of interest not reported |
| 370 | Kelly JD, Polo MR, Zoure HG, Oldenburg CE, Keenan JD, Porco TC, Lietman TM. Assessing Onchocerciasis Subcriticality from Pre-Intervention Cross-Sectional Surveys. The American journal of tropical medicine and hygiene. 2020 May 26;103(1):287. | Excluded | Outcome of interest not reported |
| 371 | Shafi Abdurahman O, Last A, Macleod D, Habtamu E, Versteeg B, Dumessa G, Guye M, Nure R, Adugna D, Miecha H, Greenland K. Trachoma risk factors in Oromia Region, Ethiopia. PLoS neglected tropical diseases. 2023 Nov 7;17(11):e0011679. | Excluded | duplicate |
| 372 | Colding-Jørgensen JT, Muheki E, Baayenda G, Harding-Esch E. Assessing water, sanitation and hygiene access and use in Nabilatuk District, Uganda: A cross-sectional study of different data collection methods. Hygiene. 2023 Mar 30;3(2):65-84. | Excluded | Outcome of interest not reported and the study done outside Ethiopia |
| 373 | Wondim MG, Dessie BA. Continuous assessment: the missing link in Ethiopian Universities. Cogent Education. 2024 Dec 31;11(1):2404778. | Excluded | Outcome of interest not reported and the study done outside Ethiopia |
| 374 | Sanders AM, Dixon R, Stuck L, Kelly M, Woods G, Muheki EM, Baayenda G, Masika M, Kafanikhale H, Mwingira U, Wohlgemuth L. Evaluation of facial cleanliness and environmental improvement activities: Lessons learned from Malawi, Tanzania, and Uganda. PLoS Neglected Tropical Diseases. 2021 Nov 29;15(11):e0009962. | Excluded | Outcome of interest not reported and the study done outside Ethiopia |
| 375 | Berihu G, Wellay T, GebreEgziabher L, Tewele A, Zewdie Y, Gebretnsae H, Birhane K, Mitiku M, Lemlem A, Dhufera A, Solomon T. Prevalence of pulmonary tuberculosis and associated risk factors among people working in two Marble Stone Factories in Tigray Region, Northern Ethiopia. Ethiopian Journal of Public Health and Nutrition (EJPHN). 2023 Jan 30;6(1):42-8. | Excluded | Outcome of interest not reported |
|  | Basha GW, Woya AA, Tekile AK. Prevalence and risk factors of active trachoma among primary school children of Amhara Region, Northwest Ethiopia. Indian Journal of Ophthalmology. 2020 May 1;68(5):750-4. | Excluded | duplicate |
| 376 | Lamah L, Kolié D, Zoumanigui A, Diallo NK, Camara M, Manet H, Millimouno TM, Camara BS, Tounkara A, Delamou A. Determinants of low coverage of the free surgical care programme for trachomatous trichiasis in rural Guinea in 2022. Tropical Medicine and Infectious Disease. 2024 Oct 11;9(10):239. | Excluded | Outcome of interest not reported and the study done outside Ethiopia |
| 377 | Alrasheed S. Systematic review and meta-analysis of childhood visual impairment in the Eastern Mediterranean Region. Eastern Mediterranean Health Journal. 2023 Jun 1;29(6). | Excluded | Outcome of interest not reported and the study done outside Ethiopia |
| 378 | Ngatse JA, Ndziessi G, Missamou F, Kinouani R, Hemilembolo M, Pion SD, Bork KA, Abena AA, Boussinesq M, Chesnais CB. Historical overview and geographical distribution of neglected tropical diseases amenable to preventive chemotherapy in the Republic of the Congo: A systematic review. PLoS Neglected Tropical Diseases. 2022 Jul 11;16(7):e0010560. | Excluded | Outcome of interest not reported and the study done outside Ethiopia |
| 379 | Zhong G, Brunham RC, de la Maza LM, Darville T, Deal C. National Institute of Allergy and Infectious Diseases workshop report:“Chlamydia vaccines: The way forward”. Vaccine. 2019 Nov 28;37(50):7346-54. | Excluded | Outcome of interest not reported and the study done outside Ethiopia |
| 380 | Schaal LF. Prevalência de tracoma em Jaú e fatores associados. | Excluded | the study done outside Ethiopia |
| 381 | Versteeg B, Vasileva H, Houghton J, Last A, Shafi Abdurahman O, Sarah V, Macleod D, Solomon AW, Holland MJ, Thomson N, Burton MJ. Viability PCR shows that non-ocular surfaces could contribute to transmission of Chlamydia trachomatis infection in trachoma. PLOS Neglected Tropical Diseases. 2020 Jul 15;14(7):e0008449. | Excluded | Outcome of interest not reported |
| 382 | Lord MG. In Defense of Mothers: Why Pregnant and Breastfeeding Women Should Be Included in Mass Drug Administration Programs. Journal of Women's Health. 2022 Sep 1;31(9):1219-21. | Excluded | Outcome of interest not reported and the study done outside Ethiopia |
| 383 | Adhikari S, Hunter E, van de Vossenberg J, Thomas J. A review of latrine front-end characteristics associated with microbial infection risk; reveals a lack of pathogen density data. International Journal of Hygiene and Environmental Health. 2023 Sep 1;254:114261. | Excluded | Outcome of interest not reported |
| 384 | Ocular Chlamydia trachomatis infection and infectious load among pre-school aged children within trachoma hyperendemic districts receiving the SAFE strategy, Amhara region, Ethiopia | Excluded | Duplicate |
| 385 | Tedijanto, Christine, et al. "Predicting future community-level ocular Chlamydia trachomatis infection prevalence using serological, clinical, molecular, and geospatial data." PLoS neglected tropical diseases 16.3 (2022): e0010273. | Excluded | Duplicate |
| 386 | Markos M, Kefyalew B, Tesfaye HB. Pooled prevalence of blindness in Ethiopia: a systematic review and meta-analysis. BMJ Open Ophthalmology. 2022 Jun 6;7(1). | Excluded | Outcome of interest not reported and non observational design |
| 387 | Grygiel-Górniak B, Folga BA. Chlamydia trachomatis—An Emerging Old Entity?. Microorganisms. 2023 May 14;11(5):1283. | Excluded | Outcome of interest not reported and the study done outside Ethiopia |
| 388 | TRACHOME IE, LA RÉGION ED, DU CAMEROUN NO, LES CAS DES DISTRICTS DP, REY TE. Kah Evans NGHA. Revue Internationale Dônni. 2023 Dec;3(2). | Excluded | Outcome of interest not reported and the study done outside Ethiopia |
| 389 | Borlase A, Prada JM, Crellen T. Modelling morbidity for neglected tropical diseases: the long and winding road from cumulative exposure to long-term pathology. Philosophical Transactions of the Royal Society B. 2023 Oct 9;378(1887):20220279. | Excluded | Outcome of interest not reported and the study done outside Ethiopia |
| 390 | Mtuy TB, Bardosh K, Ngondi J, Mwingira U, Seeley J, Burton M, Lees S. Understanding hard-to-reach communities: local perspectives and experiences of trachoma control among the pastoralist Maasai in northern Tanzania. Journal of Biosocial Science. 2021 Nov;53(6):819-38. | Excluded | Outcome of interest not reported and the study done outside Ethiopia |
| 391 | Kelly-Hope LA, Sanders AM, Harding-Esch E, Willems J, Ahmed F, Vincer F, Hill R. Complex emergencies and the control and elimination of neglected tropical diseases in Africa: developing a practical approach for implementing safe and effective mapping and intervention strategies. Conflict and Health. 2021 Dec;15:1-2. | Excluded | Outcome of interest not reported and the study done outside Ethiopia |
| 392 | Mtuy T. *Maasai Response to Mass Drug Administration for Trachoma in a Changing Political Economy in Tanzania* (Doctoral dissertation, London School of Hygiene & Tropical Medicine). | Excluded | Outcome of interest not reported and the study done outside Ethiopia |
| 393 | Chen X, Munoz B, Mkocha H, Gaydos CA, Dize L, Quinn TC, West SK. Risk of seroconversion and seroreversion of antibodies to Chlamydia trachomatis pgp3 in a longitudinal cohort of children in a low trachoma prevalence district in Tanzania. PLoS neglected tropical diseases. 2022 Jul 13;16(7):e0010629. | Excluded | Outcome of interest not reported and the study done outside Ethiopia |
| 394 | Tedijanto C, Solomon AW, Martin DL, Nash SD, Keenan JD, Lietman TM, Lammie PJ, Aiemjoy K, Amza A, Aragie S, Arzika AM. Monitoring transmission intensity of trachoma with serology. Nature communications. 2023 Jun 5;14(1):3269. | Excluded | Duplicate |
| 395 | Blindness EF, Filariasis L. Disease Elimination—Successes and Challenges. The Principles and Practice of Disease Eradication. 2024:141. | Excluded | Outcome of interest not reported |
| 396 | Bhat A, Jhanji V. Bacterial conjunctivitis. Infections of the Cornea and Conjunctiva. 2021:1-6. | Excluded | Outcome of interest not reported |
| 397 | Brady CJ, Cockrell RC, Aldrich LR, Wolle MA, West SK. A Virtual Reading Center Model Using Crowdsourcing to Grade Photographs for Trachoma: Validation Study. Journal of medical Internet research. 2023 Apr 6;25:e41233. | Excluded | Duplicate |
| 398 | Ndisabiye D. The Importance of Environmental Interventions in Eliminating Trachoma Infection in Africa: The Case of Gashoho Health District, Burundi. | Excluded | Outcome of interest not reported and the study done outside Ethiopia |
| 399 | Ghasemian E, Faal N, Pickering H, Sillah A, Breuer J, Bailey RL, Mabey D, Holland MJ. Genomic insights into local-scale evolution of ocular Chlamydia trachomatis strains within and between individuals in Gambian trachoma-endemic villages. Microbial Genomics. 2024 Mar 6;10(3):001210. | Excluded | Outcome of interest not reported and the study done outside Ethiopia |
| 400 | Adam HM, Azmerawu A, Kenee FB. Women’s Access to and Utilization of Sanitation and their Determinant Factors in Some Selected Rural Areas of East Gojjam Zone, North West Ethiopia. Ethiopian Journal of the Social Sciences and Humanities. 2021;17(2):55-90. | Excluded | Outcome of interest not reported |
| 401 | Gupta-Wright M. *Elimination at work: towards an anthropology of trachoma in Malawi* (Doctoral dissertation, London School of Hygiene & Tropical Medicine). | Excluded | Outcome of interest not reported and the study done outside Ethiopia |
| 402 | Osman MA, Alshafie BM. Post-operative Trichiasis; one year after surgery in Baladyat Elgadarif from November 2013 to 2014. | Excluded | Outcome of interest not reported and the study done outside Ethiopia |
| 403 | Delea MG, Snyder JS, Woreta M, Zewudie K, Solomon AW, Freeman MC. Development and reliability of a quantitative personal hygiene assessment tool. International journal of hygiene and environmental health. 2020 Jun 1;227:113521. | Excluded | Outcome of interest not reported |
| 404 | Gichuki PM, Kimani BW, Kanyui T, Okoyo C, Watitu T, Omondi WP, Njomo DW. Using community-based participatory approaches to improve access to mass drug administration for trachoma elimination in a pastoral conflict area of Kenya. PLOS Neglected Tropical Diseases. 2024 Nov 11;18(11):e0012653. | Excluded | Outcome of interest not reported and the study done outside Ethiopia |
| 405 | Paulet E, Contreras V, Galhaut M, Rosenkrands I, Holland M, Burton M, Dietrich J, Gallouet AS, Bosquet N, Relouzat F, Langlois S. Multimodal mucosal and systemic immune characterization of a novel non-human primate trachoma model highlights the critical role of local immunity during acute phase disease. bioRxiv. 2023 Dec 6:2023-12. | Excluded | Outcome of interest not reported |
| 406 | Wittberg DM. Sanitation, Water, and Instruction in Face-washing for Trachoma Statistical Analysis Plan. | Excluded |  |
| 407 | Reda G, Yemane D, Gebreyesus A. Prevalence and associated factors of active trachoma among 1–9 years old children in Deguatemben, Tigray, Ethiopia, 2018: community cross-sectional study. BMC ophthalmology. 2020 Dec;20:1-9. | Excluded | Duplicate |
| 408 | Ayelgn, Kessete, Tadesse Guadu, and Atalay Getachew. "Low prevalence of active trachoma and associated factors among children aged 1–9 years in rural communities of Metema District, Northwest Ethiopia: a community based cross-sectional study." *Italian Journal of Pediatrics* 47.1 (2021): 114. | Excluded | Duplicate |
| 409 | Kamuyu MK, Kelly M, Somerville S. A secondary analysis to determine countries and districts eligible for documented full geographic coverage for trichiasis case finding and outreaches. International Health. 2023 Dec;15(Supplement_2):ii53-7. | Excluded | Outcome of interest not reported and the study done outside Ethiopia |
| 410 | Karmaoui A, El Jaafari S, Chaachouay H, Hajji L. Socio-ecological factors influencing vulnerability to trachoma disease: a new tool applied in five pre-saharan provinces, Morocco. GeoJournal. 2023 Jun;88(3):2669-90. | Excluded | Outcome of interest not reported and the study done outside Ethiopia |
| 411 | Flueckiger RM. *Exploring the spatial heterogeneity of trachomatous trichiasis* (Doctoral dissertation, London School of Hygiene & Tropical Medicine). | Excluded | Outcome of interest not reported and the study done outside Ethiopia |
| 412 | Baayenda G, Mugume F, Mubangizi A, Turyaguma P, Tukahebwa EM, Byakika S, Kahwa B, Kusasira D, Bakhtiari A, Boyd S, Butcher R. Baseline prevalence of trachoma in refugee settlements in Uganda: Results of 11 population-based surveys. Ophthalmic Epidemiology. 2023 Nov 2;30(6):580-90. | Excluded | Outcome of interest not reported and the study done outside Ethiopia |
| 413 | Yirdaw G, Dessie A, Azanaw J, Birhan TA. Latrine utilization and its associated factors in urban slums dwellers of Gondar City, Northwest Ethiopia: A community-based cross-sectional study. Environmental Health Insights. 2023 Oct;17:11786302231203067 | Excluded | Outcome of interest not reported |
| 414 | Mahmud H, Haile BA, Tadesse Z, Gebresillasie S, Shiferaw A, Zerihun M, Liu Z, Callahan EK, Cotter SY, Varnado NE, Oldenburg CE. Targeted Mass Azithromycin Distribution for Trachoma: A Community-Randomized Trial (TANA II). Clinical Infectious Diseases. 2023 Aug 1;77(3):388-95. | Excluded | Duplicate |
| 415 | Manyazewal T, Davey G, Hanlon C, Newport MJ, Hopkins M, Wilburn J, Bakhiet S, Mutesa L, Semahegn A, Assefa E, Fekadu A. Innovative technologies to address neglected tropical diseases in African settings with persistent sociopolitical instability. Nature Communications. 2024 Nov 27;15(1):10274. | Excluded | Outcome of interest not reported |
| 416 | Atekem K, Harding-Esch EM, Martin DL, Downs P, Palmer SL, Kaboré A, Kelly M, Bovary A, Sarr A, Nguessan K, James F. High prevalence of trachomatous inflammation–follicular with no trachomatous trichiasis: can alternative indicators explain the epidemiology of trachoma in Côte d’Ivoire?. International Health. 2023 Dec;15(Supplement_2):ii3-11. | Excluded | Outcome of interest not reported and the study done outside Ethiopia |
| 417 | El-Moneem A, Kholoud M, Hamza MN, El-Sayed SB, Soliman AM. Clinical profile and molecular detection of Chlamydia trachomatis in follicular conjunctivitis: Insights from Egypt. Microbes and Infectious Diseases. 2024 Aug 1;5(3):1176-89. | Excluded | Outcome of interest not reported and the study done outside Ethiopia |
| 418 | Mcpherson S. *Safety of the Co-Administration of Azithromycin, Albendazole and Ivermectin Versus Standard Treatment Regimens During Mass Drug Administration (MDA) in Ethiopia: A Cluster Randomized Trial* (Doctoral dissertation, London School of Hygiene & Tropical Medicine). | Excluded | Outcome of interest not reported and non observational study design |
| 419 | Alkhidir AA, Holland MJ, Elhag WI, Williams CA, Breuer J, Elemam AE, El Hussain KM, Ournasseir ME, Pickering H. Whole-genome sequencing of ocular Chlamydia trachomatis isolates from Gadarif State, Sudan. Parasites & Vectors. 2019 Dec;12:1-1. | Excluded | Outcome of interest not reported and the study done outside Ethiopia |
| 420 | Ghasemian E, Inic-Kanada A, Collingro A, Mejdoubi L, Alchalabi H, Keše D, Elshafie BE, Hammou J, Barisani-Asenbauer T. Comparison of genovars and Chlamydia trachomatis infection loads in ocular samples from children in two distinct cohorts in Sudan and Morocco. PLoS Neglected Tropical Diseases. 2021 Aug 9;15(8):e0009655. | Excluded | Outcome of interest not reported and the study done outside Ethiopia |
| 421 | Golan R, Aviram S. Cost-effectiveness Research Report: The WASH on Wheels Project. | Excluded | Outcome of interest not reported |
| 422 | Gower EW, Munoz B, Rajak S, Habtamu E, West SK, Merbs SL, Harding JC, Alemayehu W, Callahan EK, Emerson PM, Gebre T. Pre-operative trichiatic eyelash pattern predicts post-operative trachomatous trichiasis. PLoS neglected tropical diseases. 2019 Oct 7;13(10):e0007637. | Excluded | Outcome of interest not reported |
| 423 | Ahmad B, Patel BC. Continuing Education Activity. | Excluded | Outcome of interest not reported |
| 424 | Milad D, Antaki F, Robert MC, Duval R. Development and deployment of a smartphone application for diagnosing trachoma: leveraging code-free deep learning and edge artificial intelligence. Saudi Journal of Ophthalmology. 2023 Jul 1;37(3):200-6. | Excluded | Outcome of interest not reported |
| 425 | Gebreselassie, G., Negash, K., Tsegaye, S., Makonnen, M., Deneke, B., Desalegn, M., ... & Kebede, F. (2023). Prevalence of trachoma in Somali region, Ethiopia: results from trachoma impact surveys in 50 woredas. *International Health*, *15*(Supplement_2), ii30-ii37. | Excluded | Duplicate |
| 426 | Yismaw B. *The Lived Experience of Mothers with Visual Impairment Child-Rearing Practice: The Case of Seven Mothers in Gondar Town* (Doctoral dissertation). | Excluded | Outcome of interest not reported |
| 427 | Ansah D, Weaver J, Munoz B, Bloch EM, Coles CL, Lietman T, West SK. A cross-sectional study of the availability of azithromycin in local pharmacies and associated antibiotic resistance in communities in Kilosa district, Tanzania. The American journal of tropical medicine and hygiene. 2019 Mar 25;100(5):1105. | Excluded | Outcome of interest not reported and the study done outside Ethiopia |
| 428 | Tedijanto C, Solomon AW, Martin DL, Nash SD, Keenan JD, Lietman TM, Lammie PJ, Aiemjoy K, Amza A, Aragie S, Arzika AM. Monitoring transmission intensity of trachoma with serology. Nature communications. 2023 Jun 5;14(1):3269. | Excluded | Duplicate |
| 429 | Derrick T, Ramadhani AM, Macleod D, Massae P, Mafuru E, Aiweda M, Mbuya K, Makupa W, Mtuy T, Bailey RL, Mabey DC. Immunopathogenesis of progressive scarring trachoma: results of a 4-year longitudinal study in Tanzanian children. Infection and Immunity. 2020 Mar 23;88(4):10-128. | Excluded | Outcome of interest not reported and the study done outside Ethiopia |
| 430 | Parsimei RN. *Utilization Factors of Surgical and Antibiotic Treatments for Potentially Blinding Trachoma in Loodokilani Ward, Kajiado County,(Household Survey)* (Doctoral dissertation, KeMU). | Excluded | Outcome of interest not reported and the study done outside Ethiopia |
| 431 | Morberg, D. P., Alemayehu, W., Melese, M., Lakew, T., Sisay, A., Zhou, Z., ... & Keenan, J. D. (2019). A longitudinal analysis of chlamydial infection and trachomatous inflammation following mass azithromycin distribution. Ophthalmic epidemiology, 26(1), 19-26. | Excluded | Duplicate |
| 432 | Miller DD. Chlamydia Conjunctivitis. InInfections of the Cornea and Conjunctiva 2020 Nov 28 (pp. 51-63). Singapore: Springer Singapore. | Excluded | Outcome of interest not reported and the study done outside Ethiopia |
| 434 | Hassan EU, Apadinuwe SC, Bisanzio D, Dejene M, Downs P, Harding-Esch EM, Jimenez C, Kabona G, Kebede BN, Kelly M, Kivumbi P. Impact of personal protective equipment on the clarity of vision among trachoma survey graders and trichiasis surgeons in the context of COVID-19. BMJ open ophthalmology. 2023 Jun 8;8(1). | Excluded | Outcome of interest not reported and the study done outside Ethiopia |
| 435 | Jawaid M, Al-Khairy S, Siddiqui F, Azeem A. Frequency of Different Ocular Conditions Leading to Ocular Morbidity in Pediatric Age Group at Dow University Hospital. Pakistan Journal of Ophthalmology. 2021 Sep 3;37(4). | Excluded | Outcome of interest not reported and the study done outside Ethiopia |
| 436 | Hagedorn BL, Han R, McCarthy KA. One size does not fit all: an application of stochastic modeling to estimating primary healthcare needs in Ethiopia at the sub-national level. BMC Health Services Research. 2023 Oct 6;23(1):1070. | Excluded | Outcome of interest not reported |
| 437 | Muche, Natnael, et al. "Behavioral Responses for Face Cleanliness Message to Prevent Trachoma Among Mothers Having Children Age 1–9 Years Old, in Fogera District, Northwest Ethiopia: An Application of Extended Parallel Process Model." *International Journal of General Medicine* (2023): 1927-1941. | Excluded | Duplicate |
| 438 | Paulet E, Contreras V, Galhaut M, Rosenkrands I, Holland M, Burton M, Dietrich J, Gallouet AS, Bosquet N, Relouzat F, Langlois S. Multimodal mucosal and systemic immune characterization of a non-human primate trachoma model highlights the critical role of local immunity during acute phase disease. PLOS Neglected Tropical Diseases. 2024 Aug 2;18(8):e0012388. | Excluded | Outcome of interest not reported and the study done outside Ethiopia |
| 439 | Wolle MA, Misra N, Naufal F, Saheb Kashaf M, Munoz BE, Mkocha H, Funga N, West SK. The Association Between the Severity of Trachomatous Scarring and Trachomatous Trichiasis Severity in Surgical Patients in Tanzania. Ophthalmic Epidemiology. 2024 Nov 1;31(6):561-7. | Excluded | Outcome of interest not reported and the study done outside Ethiopia |
| 440 | Senbete, Lissanu, and Girmay Adhena. "Magnitude of Active Trachoma Among Host and Refugee Children in Gambella Regional State, Ethiopia." *Clinical Ophthalmology* (2024): 777-789. | Excluded | Duplicate |
| 44 | Senyonjo L, Downs P, Schmidt E, Bailey R, Blanchet K. Lessons learned for surveillance strategies for trachoma elimination as a public health problem, from the evaluation of approaches utilised by Guinea worm and onchocerciasis programmes: A literature review. PLoS neglected tropical diseases. 2021 Jan 28;15(1):e0009082. | Excluded | Outcome of interest not reported and the study done outside Ethiopia |
| 442 | Chen X, Munoz B, Wolle MA, Woods G, Odonkor M, Naufal F, Mkocha H, West SK. Environmental factors and hygiene behaviors associated with facial cleanliness and trachoma in Kongwa, Tanzania. PLoS neglected tropical diseases. 2021 Oct 28;15(10):e0009902. | Excluded | Outcome of interest not reported and the study done outside Ethiopia |
| 443 | Joye AS, Firlie MG, Wittberg DM, Aragie S, Nash SD, Tadesse Z, Dagnew A, Hailu D, Admassu F, Wondimteka B, Getachew H. Computer vision identification of trachomatous inflammation-follicular using deep learning. Cornea. 2022 May 13:10-97. | Excluded | Outcome of interest not reported |
| 444 | Delea MG, Snyder JS, Belew M, Caruso BA, Garn JV, Sclar GD, Woreta M, Zewudie K, Gebremariam A, Freeman MC. Design of a parallel cluster-randomized trial assessing the impact of a demand-side sanitation and hygiene intervention on sustained behavior change and mental well-being in rural and peri-urban Amhara, Ethiopia: Andilaye study protocol. BMC Public Health. 2019 Dec;19:1-5. | Excluded | Outcome of interest not reported |
| 445 | Adane, Balew, et al. "Determinants of Trachomatous Inflammation-Follicular Among Children Aged 1 to 9 Years Old in a Rural Area of Gozamn District, Northwestern Ethiopia: A Matched Case-Control Study." *Environmental Health Insights* 17 (2023): 11786302231169941. | Excluded | Duplicate |
| 446 | Doyore F, Garmaroudi G, Sadeghi R, Shakibazadeh E, Yaseri M, Birhanu Z, Bekele T. Application of Kingdon and Hall Models to Review Environmental Sanitation and Health Promotion Policy in Ethiopia. Ethiopian Journal of Health Sciences. 2019;29(2):277-86. | Excluded | Outcome of interest not reported |
| 447 | Rehim MH, Jamil AZ, Hameed S, Raiz S, Butt AM. Assessment of Trachoma Awareness in General Population of Sahiwal. Ophthalmology Update. 2019 Oct 1;17(4). | Excluded | Outcome of interest not reported and the study done outside Ethiopia |
| 448 | World Health Organization. WHO guideline on mass drug administration of azithromycin to children under five years of age to promote child survival. World Health Organization; 2020 Sep 30. | Excluded | Outcome of interest not reported |
| 449 | Romani L, Marks M, Sokana O, Nasi T, Kamoriki B, Cordell B, Wand H, Whitfeld MJ, Engelman D, Solomon AW, Kaldor JM. Efficacy of mass drug administration with ivermectin for control of scabies and impetigo, with coadministration of azithromycin: a single-arm community intervention trial. The Lancet Infectious Diseases. 2019 May 1;19(5):510-8. | Excluded | Outcome of interest not reported and the study done outside Ethiopia |
| 450 | Tanywe AC, Matchawe C, Fernandez R, Lapkin S. Perceptions and practices of community members relating to trachoma in Africa: a qualitative systematic review protocol. JBI Evidence Synthesis. 2019 Nov 1;17(11):2350-6. | Excluded | Outcome of interest not reported and the study done outside Ethiopia |
| 451 | Goodhew EB, Taoaba R, Harding-Esch EM, Gwyn SE, Bakhtiari A, Butcher R, Cama A, Guagliardo SA, Jimenez C, Mpyet CD, Tun K. Changes in trachoma indicators in Kiribati with two rounds of azithromycin mass drug administration, measured in serial population-based surveys. PLOS Neglected Tropical Diseases. 2023 Jul 7;17(7):e0011441. | Excluded | duplicate |
| 452 | Nayel Y, Muñoz BE, Mkocha H, West SK, Wolle MA. Expanding a photographic grading system for trachomatous scarring. International health. 2023 Dec;15(Supplement_2):ii25-9. | Excluded | Outcome of interest not reported and the study done outside Ethiopia |
| 453 | Seidu AA, Ahinkorah BO, Kissah-Korsah K, Agbaglo E, Dadzie LK, Ameyaw EK, Budu E, Hagan Jr JE. A multilevel analysis of individual and contextual factors associated with the practice of safe disposal of children’s faeces in sub-Saharan Africa. PloS one. 2021 Aug 2;16(8):e0254774. | Excluded | Outcome of interest not reported and the study done outside Ethiopia |
| 454 | Muche, Natnael, et al. "Behavioral Responses for Face Cleanliness Message to Prevent Trachoma Among Mothers Having Children Age 1–9 Years Old, in Fogera District, Northwest Ethiopia: An Application of Extended Parallel Process Model." *International Journal of General Medicine* (2023): 1927-1941. | Excluded | Duplicate |
| 455 |  | Excluded |  |
| 456 | Alshamahi EY, Al-Eryani SA, Jaadan BM, Al-Shamahy HA, Al Haddad AA, Al-Zazai BA. The national campaign for the mass treatment of trachoma: monitoring coverage and practices of Mass Drug Administration (MDA) in Yemen-follow-up study. The national campaign for the mass treatment of trachoma: monitoring coverage and practices of Mass Drug Administration (MDA) in Yemen-follow-up study. 2021. | Excluded | the study done outside Ethiopia |
| 457 | Eshete A, Haddis A, Mengistie E. Investigation of environmental and health impacts solid waste management problems and associated factors in Asella town, Ethiopia. Heliyon. 2024 Mar 30;10(6). | Excluded | Outcome of interest not reported |
| 458 | Kishiru A, Azage M, Zewale TA, Bogale KA. Latrine utilization and its associated factors among Rural Communities of North Achefer District, Amhara Region, Northwest Ethiopia. | Excluded | Outcome of interest not reported |
| 459 | EY AA, Al-Shamahy HA, KA AA. Hizam Al-Arosi SA.(2022). National Comprehensive Trachoma Treatment Campaign: Community Monitoring of Mass Drug Administration (MDA) Coverage and Practices. J. Clinical Research and Reports.;11(2):2690-1919. | Excluded | Outcome of interest not reported |
| 460 | Malik G, Dhanawat M, Wilson K. Neglected tropical diseases. InEmerging Approaches to Tackle Neglected Diseases: From Molecule to End Product 2024 Jun 21 (pp. 1-15). Bentham Science Publishers. | Excluded | Outcome of interest not reported |
| 461 | Kanda A, Ncube EJ, Voyi K. Effect of sanitation interventions on health outcomes: a systematic review of cluster-randomized controlled trials in rural communities of low-and middle-income countries. International Journal of Environmental Research and Public Health. 2021 Aug 5;18(16):8313. | Excluded | Outcome of interest not reported |
| 462 | Ndisabiye D, Waters EK, Gore R, Sidhu H. Do poor environmental conditions drive trachoma transmission in Burundi? A mathematical modelling study. The ANZIAM Journal. 2021 Oct;63(4):434-47. | Excluded | Outcome of interest not reported and the study done outside Ethiopia |
| 463 | He J, Chen A, Zou M, Young CA, Jin L, Zheng D, Jin G, Congdon N. Time trends and heterogeneity in the disease burden of trachoma, 1990–2019: a global analysis. British Journal of Ophthalmology. 2023 Mar 1;107(3):337-41. | Excluded | Outcome of interest not reported and the study done outside Ethiopia |
| 464 | Dattani S, Spooner F. Trachoma: how a common cause of blindness can be prevented worldwide. Our World in Data. 2024 Jun 17. | Excluded | Outcome of interest not reported |
| 465 | Ramadhani AM, Derrick T, Macleod D, Massae P, Malisa A, Mbuya K, Mtuy T, Makupa W, Roberts CH, Bailey RL, Mabey DC. Ocular immune responses, Chlamydia trachomatis infection and clinical signs of trachoma before and after azithromycin mass drug administration in a treatment naïve trachoma-endemic Tanzanian community. PLoS neglected tropical diseases. 2019 Jul 15;13(7):e0007559. | Excluded | Outcome of interest not reported and the study done outside Ethiopia |
| 467 | Gebreselassie, G., Negash, K., Tsegaye, S., Makonnen, M., Deneke, B., Desalegn, M., ... & Kebede, F. (2023). Prevalence of trachoma in Somali region, Ethiopia: results from trachoma impact surveys in 50 woredas. *International Health*, *15*(Supplement_2), ii30-ii37. | Excluded | duplicate |
| 468 | Mack I, Sharland M, Berkley JA, Klein N, Malhotra-Kumar S, Bielicki J. Antimicrobial resistance following azithromycin mass drug administration: potential surveillance strategies to assess public health impact. Clinical Infectious Diseases. 2020 Mar 17;70(7):1501-8. | Excluded | Outcome of interest not reported |
| 469 | Pickering H, Ramadhani AM, Massae P, Mafuru E, Malisa A, Mbuya K, Makupa W, Mtuy T, Derrick T, Houghton J, Bailey RL. The conjunctival microbiome before and after azithromycin mass drug administration for trachoma control in a cohort of Tanzanian children. Frontiers in public health. 2022 Oct 17;10:1015714. | Excluded | Outcome of interest not reported and the study done outside Ethiopia |
| 470 | Reda G, Yemane D, Gebreyesus A. Prevalence and associated factors of active trachoma among 1–9 years old children in Deguatemben, Tigray, Ethiopia, 2018: community cross-sectional study. BMC ophthalmology. 2020 Dec;20:1-9. | Excluded | Duplicate |
| 471 | Senbete, Lissanu, and Girmay Adhena. "Magnitude of Active Trachoma Among Host and Refugee Children in Gambella Regional State, Ethiopia." *Clinical Ophthalmology* (2024): 777-789. | Excluded | Duplicate |
| 472 | Martini M, Riccardi N, Simonetti O, Orsini D, Samassa F, Parodi A. “The blinding disease”. The history of trachoma in Italians between the 19th and 20th centuries: colonial or national blindness?. Pathogens and Global Health. 2024 Aug 17;118(6):499-504. | Excluded | Outcome of interest not reported and the study done outside Ethiopia |
| 473 | Novotný J, Mamo BG. Household-level sanitation in Ethiopia and its influencing factors: a systematic review. BMC Public Health. 2022 Jul 29;22(1):1448. | Excluded | Outcome of interest not reported |
| 474 | Robinson A, Versteeg B, Abdurahman OS, Clatworthy I, Shuka G, Debela D, Hordofa G, Reis de Oliveira Gomes L, Abraham Aga M, Dumessa G, Sarah V. Field-and laboratory-based studies on correlates of Chlamydia trachomatis transmission by Musca sorbens: Determinants of fly-eye contact and investigations into fly carriage of elementary bodies. PLOS Neglected Tropical Diseases. 2024 Jul 2;18(7):e0012280. | Excluded | Outcome of interest not reported and the study done outside Ethiopia |
| 475 | Mtuy TB, Burton MJ, Mwingira U, Ngondi JM, Seeley J, Lees S. Knowledge, perceptions and experiences of trachoma among Maasai in Tanzania: Implications for prevention and control. PLoS neglected tropical diseases. 2019 Jun 24;13(6):e0007508. | Excluded | Outcome of interest not reported and the study done outside Ethiopia |
| 476 | Tedijanto C, Solomon AW, Martin DL, Nash SD, Keenan JD, Lietman TM, Lammie PJ, Aiemjoy K, Amza A, Aragie S, Arzika AM. Monitoring transmission intensity of trachoma with serology. Nature communications. 2023 Jun 5;14(1):3269. | Excluded | Duplicate |
| 477 | Edwards T. *Heterogeneity in cluster randomised trials of azithromycin mass drug administration for trachoma control* (Doctoral dissertation, London School of Hygiene & Tropical Medicine). | Excluded | Outcome of interest not reported and non-observational study design |
| 478 | Handley BL, Butcher R, Taoaba R, h Roberts C, Cama A, Müeller A, Solomon AW, Tekeraoi R, Marks M. Absence of serological evidence of exposure to Treponema pallidum among children suggests yaws is no longer endemic in Kiribati. The American journal of tropical medicine and hygiene. 2019 Feb 4;100(4):940. | Excluded | Outcome of interest not reported and the study done outside Ethiopia |
| 479 | Adane, Balew, et al. "Determinants of Trachomatous Inflammation-Follicular Among Children Aged 1 to 9 Years Old in a Rural Area of Gozamn District, Northwestern Ethiopia: A Matched Case-Control Study." *Environmental Health Insights* 17 (2023): 11786302231169941. | Excluded | Duplicate |
| 480 | Idris A, Ibrahim UF, Habib SG, Duke R. Predictors of Acceptance and Barriers to the Uptake of Mass Drug Administration Program for Eliminating Trachoma in Taura District, Jigawa State Nigeria: A Mixed Methods Study. Nigerian Journal of Ophthalmology. 2024 Sep 1;32(3):88-97. | Excluded | Outcome of interest not reported and the study done outside Ethiopia |
| 481 | Mahmud H, Haile BA, Tadesse Z, Gebresillasie S, Shiferaw A, Zerihun M, Keenan JD, Lietman T. Comparing targeted azithromycin treatment strategies in a trachoma hyperendemic area. Investigative Ophthalmology & Visual Science. 2022 Jun 1;63(7):3565-A0452. | Excluded | Duplicate |
| 482 | Amza A, Kadri B, Nassirou B, Arzika AM, Austin A, Nyatigo F, Lebas E, Arnold BF, Lietman TM, Oldenburg CE. Azithromycin Reduction to Reach Elimination of Trachoma (ARRET): study protocol for a cluster randomized trial of stopping mass azithromycin distribution for trachoma. BMC ophthalmology. 2021 Dec;21:1-6. | Excluded | Outcome of interest not reported |
| 483 | Sullivan KM. *Optimizing Safe Strategy Components for Trachoma Elimination* (Doctoral dissertation, The University of North Carolina at Chapel Hill). | Excluded | Outcome of interest not reported |
| 484 | Issifou AA, Dare A, Badou GA, Harding-Esch EM, Solomon AW, Bakhtiari A, Boyd S, Jimenez C, Harte A, Burgert-Brucker CR, Sintondji FR. Twenty-Three Population-Based Trachoma Prevalence Surveys in the Central and Northern Regions of Benin, 2018–2022. Ophthalmic Epidemiology. 2024 Nov 1;31(6):498-508. | Excluded | the study done outside Ethiopia |
| 485 | ul Hassan E, Kelly M, Waititu T, Olobio N, Kabona G, Mkocha H, Kivumbi P, Mwale C, Mubangizi A, Mugume F, Baayenda G. Productivity, efficiency and gender equity of community mobilisation approaches in trichiasis campaigns: analysis of programmatic data from seven sub-Saharan African countries. International Health. 2022 Apr;14(Supplement_1):i24-8. | Excluded | Outcome of interest not reported and the study done outside Ethiopia |
| 486 | Mahmud H, Haile BA, Tadesse Z, Gebresillasie S, Shiferaw A, Zerihun M, Liu Z, Callahan EK, Cotter SY, Varnado NE, Oldenburg CE. Targeted Mass Azithromycin Distribution for Trachoma: A Community-Randomized Trial (TANA II). Clinical Infectious Diseases. 2023 Aug 1;77(3):388-95. | Excluded | Duplicate |
| 487 | Aysheshim A. *Prevalence and Associated Factors of Postoperative Trichiasis Among Adults in Ayehu Guagusa District, North-West Ethiopia: Community Based Crosssectional Study* (Doctoral dissertation). | Excluded | Outcome of interest not reported |
| 488 | Sanders AM, Makoy S, Deathe AR, Ohidor S, Jesudason TC, Nute AW, Odongi P, Boniface L, Abuba S, Delahaut AS, Sebit W. Cost and community acceptability of enhanced antibiotic distribution approaches for trachoma in the Republic of South Sudan: enhancing the A in SAFE (ETAS) study protocol. BMC ophthalmology. 2023 Feb 6;23(1):51. | Excluded | Outcome of interest not reported and the study done outside Ethiopia |
| 489 | Glagn Abdilwohab M, Hailemariam Abebo Z. High prevalence of clinically active trachoma and its associated risk factors among preschool-aged children in arba minch health and demographic surveillance site, southern Ethiopia. Clinical Ophthalmology. 2020 Nov 2:3709-18. | Excluded | Duplicate |
| 490 | Lynch KD, Apadinuwe SC, Lambert SB, Hillgrove T, Starr M, Catlett B, Ware RS, Cama A, Webster S, Harding-Esch EM, Bakhtiari A. A national survey integrating clinical, laboratory, and WASH data to determine the typology of trachoma in Nauru. PLoS neglected tropical diseases. 2022 Apr 19;16(4):e0010275 | Excluded | Outcome of interest not reported and the study done outside Ethiopia |
| 491 | Yafanna GA, Musa D, Abubakar NS, Abubakar H, Kode S, Alhassan MB. Awareness of Trachoma Prevention among People of Bolori Community Maiduguri Metropolitan Council Local Government Area of Borno State, Nigeria. Bayero Journal of Nursing and Health Care. 2022 Nov 4;4(1):949-58. | Excluded | Outcome of interest not reported and the study done outside Ethiopia |
| 492 | Solomon AW, Hooper PJ, Bangert M, Mwingira UJ, Bakhtiari A, Brady MA, Fitzpatrick C, Jones I, Kabona G, Kello AB, Millar T. The importance of failure: how doing impact surveys that fail saves trachoma programs money. The American Journal of Tropical Medicine and Hygiene. 2020 Oct 5;103(6):2481. | Excluded | Outcome of interest not reported |
| 493 | Blumberga S, Borlaseb A, Pradac JM, Solomond AW, Emersone P, Hoopere PJ, Deinera MS, Amoahf B, Hollingsworthb D, Porcoa TC, Lietman TM. Implications of the COVID-19 pandemic on eliminating trachoma as a public health problem. | Excluded | Outcome of interest not reported |
| 494 | Epee E, Tagne CF, Bakhtiari A, Boyd S, Willis R, Harte AJ, Jimenez C, Burgert-Brucker C, Goldman W, Kello AB, Palmer S. Assessing the prevalence of trachoma in the East, North, Far North and Adamaoua regions of Cameroon, 2016–2022. International Health. 2024 Nov 14:ihae071. | Excluded | Outcome of interest not reported and the study done outside Ethiopia |
| 495 | Barton A, Rosenkrands I, Pickering H, Faal N, Harte A, Joof H, Makalo P, Ragonnet M, Olsen AW, Bailey RL, Mabey DC. A systems serology approach to the investigation of infection-induced antibody responses and protection in trachoma. Frontiers in immunology. 2023 May 23;14:1178741. | Excluded | Outcome of interest not reported and the study done outside Ethiopia |
| 496 | Morberg, D. P., Alemayehu, W., Melese, M., Lakew, T., Sisay, A., Zhou, Z., ... & Keenan, J. D. (2019). A longitudinal analysis of chlamydial infection and trachomatous inflammation following mass azithromycin distribution. Ophthalmic epidemiology, 26(1), 19-26. | Excluded | duplicate |
| 497 | West SK. Toward the elimination of disease: The 2019 Weisenfeld Award lecture. Investigative Ophthalmology & Visual Science. 2019 Nov 1;60(14):4805-10. | Excluded | Outcome of interest not reported |
| 498 | Sherief ST, Sitotaw MS, Girma A. Traditional Eye Medicine and self-treatment in Rural Ethiopia. | Excluded | Outcome of interest not reported |
| 499 | Nasir MA, Elsawy F, Omar A, Haque SO, Nadir R. Eliminating trachoma by 2020: assessing progress in Nigeria. Cureus. 2020 Jul 29;12(7). | Excluded | Outcome of interest not reported and the study done outside Ethiopia |
| 500 | Baye D. Sustainable development goals (SDG) target 6.2 in Ethiopia: challenges and opportunities. Open Access Library Journal. 2021 May 6;8(5):1-28. | Excluded | Outcome of interest not reported |
| 501 | Maritim P, Zulu JM, Jacobs C, Chola M, Chongwe G, Zyambo J, Halwindi H, Michelo C. Factors shaping the implementation of the SAFE strategy for trachoma using the Consolidated Framework for Implementation Research: a systematic review. Global health action. 2019 Jan 1;12(1):1570646. | Excluded | Outcome of interest not reported |
| 502 | Cha S, Jung S, Abera T, Beyene ET, Schmidt WP, Ross I, Jin Y, Bizuneh DB. Performance of Pit Latrines and Their Herd Protection Against Diarrhea: A Longitudinal Cohort Study in Rural Ethiopia. Global Health: Science and Practice. 2024 Jun 27;12(3). | Excluded | Outcome of interest not reported |
| 503 | Mahmud H, Landskroner E, Amza A, Aragie S, Godwin WW, de Hostos Barth A, O’Brien KS, Lietman TM, Oldenburg CE. Stopping azithromycin mass drug administration for trachoma: A systematic review. PLoS Neglected Tropical Diseases. 2021 Jul 8;15(7):e0009491. | Excluded | Duplicate |
| 504 | Chavda VP, Pandya A, Kypreos E, Patravale V, Apostolopoulos V. Chlamydia trachomatis: quest for an eye-opening vaccine breakthrough. Expert Review of Vaccines. 2022 Jun 3;21(6):771-81. | Excluded | Outcome of interest not reported |
| 505 | Naufal F, Brady CJ, Wolle MA, Saheb Kashaf M, Mkocha H, Bradley C, Kabona G, Ngondi J, Massof RW, West SK. Evaluation of photography using head-mounted display technology (ICAPS) for district trachoma surveys. PLoS neglected tropical diseases. 2021 Nov 8;15(11):e0009928. | Excluded | Outcome of interest not reported |
| 506 | Sanders AM, Elshafie BE, Abdalla Z, Simmons C, Goodhew EB, Gonzalez TA, Nute AW, Mohammed A, Callahan EK, Martin DL, Nash SD. Serological responses to trachoma antigens prior to the start of mass drug administration: Results from population-based baseline surveys, North Darfur, Sudan. The American journal of tropical medicine and hygiene. 2024 Mar 19;111(3 Suppl):49. | Excluded | Outcome of interest not reported and the study done outside Ethiopia |
| 507 | Flueckiger RM, Courtright P, Abdala M, Abdou A, Abdulnafea Z, Al-Khatib TK, Amer K, Amiel ON, Awoussi S, Bakhtiari A, Batcho W. The global burden of trichiasis in 2016. PLoS neglected tropical diseases. 2019 Nov 25;13(11):e0007835. | Excluded | Duplicate |
| 508 | Lugoe N. *Effectiveness of community interventions to eliminate trachomatous inflammation–follicular in Kongwa And Mpwapwa Districts, Dodoma Region* (Doctoral dissertation, Muhimbili University of Health and Allied Sciences). | Excluded | Outcome of interest not reported and the study done outside Ethiopia |
| 509 | Oldenburg CE, Aragie S, Amza A, Solomon AW, Brogdon J, Arnold BF, Keenan JD, Lietman TM. Can we eradicate trachoma? A survey of stakeholders. British Journal of Ophthalmology. 2021 Aug 1;105(8):1059-62. | Excluded | Outcome of interest not reported |
| 510 | Abubakar Y, Egbuna C, Olatunde A, Tijjani H, Odoh UE. Medicinal Plants and Phytochemicals Effective Against Chlamydia trachomatis, the Causative Agent of Trachoma. Neglected Tropical Diseases and Phytochemicals in Drug Discovery. 2021 Sep 22:381-96. | Excluded | Outcome of interest not reported and the study done outside Ethiopia |
| 511 | Agide FD, Garmaroudi G, Sadeghi R, Shakibazadeh E, Yaseri M, Koricha ZB, Tefese TB. Application of Kingdon and Hall Models to review environmental sanitation and health promotion policy in Ethiopia: a professional perspective as a review. Ethiopian journal of health sciences. 2019;29(2). | Excluded | Outcome of interest not reported and non observational design |
| 512 | Bisanzio D, Butcher R, Turbé V, Matsumoto K, Dinesh C, Massae P, Dejene M, Jimenez C, Macleod C, Matayan E, Mpyet C. Accuracy, acceptability and feasibility of photography for use in trachoma surveys: a mixed methods study in Tanzania. International health. 2024 Jul;16(4):416-27 | Excluded | Outcome of interest not reported and the study done outside Ethiopia |
| 513 | Beyene GA, Beyene NA, Fekadu GA. Factors associated with active trachoma among children in ebinat district, South Gondar Zone, North West Ethiopia: A community-based cross-sectional study. medRxiv. 2022 Feb 7:2022-02. | Excluded | Duplicate |
| 514 | Harding-Esch EM, Holland MJ, Schémann JF, Sissoko M, Sarr B, Butcher RM, Molina-Gonzalez S, Andreasen AA, Mabey DC, Bailey RL. Facial cleanliness indicators by time of day: results of a cross-sectional trachoma prevalence survey in Senegal. Parasites & Vectors. 2020 Dec;13:1-1. | Excluded | Outcome of interest not reported and the study done outside Ethiopia |
| 515 | Mabey AC, Sokana O, Taleo F, Taylor12 HR, Anthony W. Conjunctival scarring, corneal pannus and Herbert’s pits in adolescent children in trachoma-endemic populations of the Solomon Islands and Vanuatu. | Excluded | Outcome of interest not reported and the study done outside Ethiopia |
| 516 | Kayiwa D, Murungu R, Watako D, Radooli MO, Sembuche J, Bolawole O. Contribution of Hygiene Behavior Change interventions in trachoma elimination efforts in Uganda: A case study of Napak and Nakapiripirit districts. OIDA International Journal of Sustainable Development. 2020 Jul 17;13(02):75-92. | Excluded | Outcome of interest not reported and the study done outside Ethiopia |
| 517 | Eshetu D, Kifle T, Hirigo AT. Knowledge, attitudes, and practices of hand washing among aderash primary schoolchildren in Yirgalem Town, Southern Ethiopia. Journal of Multidisciplinary Healthcare. 2020 Aug 7:759-68. | Excluded | Outcome of interest not reported |
| 518 | Senbete, Lissanu, and Girmay Adhena. "Magnitude of Active Trachoma Among Host and Refugee Children in Gambella Regional State, Ethiopia." *Clinical Ophthalmology* (2024): 777-789. | Ecluded | duplicate |
| 519 | Ramadhani AM, Derrick T, Macleod D, Massae P, Mafuru E, Malisa A, Mbuya K, Roberts CH, Makupa W, Mtuy T, Bailey RL. Progression of scarring trachoma in Tanzanian children: A four-year cohort study. PLoS neglected tropical diseases. 2019 Aug 14;13(8):e0007638. | Excluded | Outcome of interest not reported and the study done outside Ethiopia |
| 520 | Bah MM, Sakho F, Goepogui A, Nieba LC, Cisse A, Courtright P, Harte AJ, Burgert-Brucker C, Jimenez C, Lama PL, Sagno M. The Prevalence of Trachomatous Trichiasis in People Aged 15 Years and Over in Six Evaluation Units of Gaoual, Labé, Dalaba and Beyla Districts, Guinea. Ophthalmic epidemiology. 2024 Nov 1;31(6):526-33. | Excluded | Outcome of interest not reported and the study done outside Ethiopia |
| 521 | Butcher R, Tagabasoe J, Manemaka J, Bong A, Garae M, Daniel L, Roberts C, Handley BL, Hu VH, Harding-Esch EM, Bakhtiari A. Conjunctival scarring, corneal pannus, and herbert’s Pits in adolescent children in trachoma-endemic populations of the Solomon Islands and Vanuatu. Clinical Infectious Diseases. 2021 Nov 1;73(9):e2773-80. | Excluded | Outcome of interest not reported and the study done outside Ethiopia |
| 522 | Senyonjo L, Aboe A, Bailey R, Agyemang D, Marfo B, Wanye S, Schmidt E, Addy J, Blanchet K. Operational adaptations of the trachoma pre-validation surveillance strategy employed in Ghana: a qualitative assessment of successes and challenges. Infectious diseases of poverty. 2019 Dec;8:1-1. | Excluded | Outcome of interest not reported and the study done outside Ethiopia |
| 523 | Zewdie A, Dugassa W, Mannekulih E, Kaba Z, Wondimu R. Latrine utilization and associated factors among households in SebetaHawas woreda, Oromia Special Zone, Ethiopia. Eur J Clin Biomed Sci. 2021;7:44. | Excluded | Outcome of interest not reported |
| 524 | Mosenia A, Haile BA, Shiferaw A, Gebresillasie S, Gebre T, Zerihun M, Tadesse Z, Emerson PM, Callahan EK, Zhou Z, Lietman TM. When the Neighboring Village is Not Treated: Role of Geographic Proximity to Communities Not Receiving Mass Antibiotics for Trachoma. Clinical Infectious Diseases. 2023 Mar 15;76(6):1038-42. | Excluded | Outcome of interest not reported |
| 525 | Macleod CK, Bailey RL, Dejene M, Shafi O, Kebede B, Negussu N, Mpyet C, Olobio N, Alada J, Abdala M, Willis R. funding. | Excluded | Outcome of interest not reported |
| 526 | Hammou J, Guagliardo SA, Obtel M, Razine R, Haroun AE, Youbi M, Bellefquih AM, White M, Gwyn S, Martin DL. Post-validation survey in two districts of Morocco after the elimination of trachoma as a public health problem. The American Journal of Tropical Medicine and Hygiene. 2022 Mar 28;106(5):1370. | Excluded | the study done outside Ethiopia |
| 527 | Ahmad B, Zeppieri M, Patel BC. Trachoma. InStatPearls [Internet] 2024 Apr 20. StatPearls Publishing. | Excluded | Outcome of interest not reported |
| 528 | Amoah B, Fronterre C, Johnson O, Dejene M, Seife F, Negussu N, Bakhtiari A, Harding-Esch EM, Giorgi E, Solomon AW, Diggle PJ. IEA. International Journal of Epidemiology. 2022;468:478. | Excluded | Outcome of interest not reported |
| 529 | Muche, Natnael, et al. "Behavioral Responses for Face Cleanliness Message to Prevent Trachoma Among Mothers Having Children Age 1–9 Years Old, in Fogera District, Northwest Ethiopia: An Application of Extended Parallel Process Model." *International Journal of General Medicine* (2023): 1927-1941. | Excluded | Duplicate |
| 530 | Kanyi S, Hydara A, Sillah A, Mpyet C, Harte A, Bakhtiari A, Willis R, Jimenez C, Aboe A, Bailey R, Harding-Esch EM. The Gambia Trachomatous Trichiasis Surveys: Results from Five Evaluation Units Confirm Attainment of Trachoma Elimination Thresholds. Ophthalmic epidemiology. 2024 Nov 1;31(6):534-42 | Excluded | Outcome of interest not reported and the study done outside Ethiopia |
|  | Water, Sanitation, and Hygiene (WASH) Factors Influencing the Effectiveness of Mass Drug Administration to Eliminate Trachoma as a Public Health Problem in Malawi |  | Wrong setting and outcome |
| 531 | Wang EY, Kong X, Wolle M, Gasquet N, Ssekasanvu J, Mariotti SP, Bourne R, Taylor H, Resnikoff S, West S. Global trends in blindness and vision impairment resulting from corneal opacity 1984–2020: A meta-analysis. Ophthalmology. 2023 Aug 1;130(8):863-71. | Excluded | Outcome of interest not reported |
| 532 | Khan AA, Florea VV, Hussain A, Jadoon Z, Boisson S, Willis R, Dejene M, Bakhtiari A, Mpyet C, Pavluck AL, Gillani M. Prevalence of Trachoma in Pakistan: results of 42 population-based prevalence surveys from the global trachoma mapping project. Ophthalmic epidemiology. 2020 Mar 3;27(2):155-64. | Excluded | Duplicate |
| 533 | Merali FI, Schein OD. Epidemiology of corneal diseases. InFoundations of Corneal Disease: Past, Present and Future 2019 Nov 1 (pp. 307-330). Cham: Springer International Publishing. | Excluded | Outcome of interest not reported |
| 534 | Mabey D. How many neglected tropical diseases can we eliminate by 2030? RSTMH Presidential Address. Transactions of The Royal Society of Tropical Medicine and Hygiene. 2020 Jul 1;114(7):473-5. | Excluded | Outcome of interest not reported |
| 535 | Glagn Abdilwohab M, Hailemariam Abebo Z. High prevalence of clinically active trachoma and its associated risk factors among preschool-aged children in arba minch health and demographic surveillance site, southern Ethiopia. Clinical Ophthalmology. 2020 Nov 2:3709-18. | Excluded | Duplicate |
| 536 | Renneker KK, Abdala M, Addy J, Al-Khatib T, Amer K, Badiane MD, Batcho W, Bella L, Bougouma C, Bucumi V, Chisenga T. Elimination Delayed is Not Elimination Denied: Progress Toward GET2020. | Excluded | Outcome of interest not reported |
| 537 | Amza A, Kadri B, Nassirou B, Arzika A, Gebreegziabher E, Hu H, Zhong L, Chen C, Yu D, Abraham T, Liu Y. Seroepidemiology of trachoma in a low prevalence region receiving annual mass azithromycin distribution in Maradi, Niger. PLOS Neglected Tropical Diseases. 2024 Dec 9;18(12):e0012727. | Excluded | Outcome of interest not reported and the study done outside Ethiopia |
| 538 | Ofoegbu OO. Evaluation of trichiasis surgeons' performance in selected trachoma endemic African countries. | Excluded | Outcome of interest not reported and the study done outside Ethiopia |
| 539 | Seyum D, Fetene N, Kifle T, Negash H, Kabeto T, Gebre M, Data T, Tadele T, Abayo G, Wondimu A, Butcher R. Prevalence of trachoma from 66 impact surveys in 52 woredas of Southern Nations, Nationalities and Peoples’ and Sidama Regions of Ethiopia, 2017–2019. Ophthalmic epidemiology. 2023 Nov 2;30(6):637-46. | Excluded | Duplicate |
| 540 | Saboyá-Díaz MI, Carey Angeles CA, Avellaneda Yajahuanca RD, Meléndez Ruíz SK, Cabrera R, Honorio Morales HA, Pachas PE, Guardo M, Renneker KK, Muñoz BE, West SK. Associated factors of the co-occurrence of trachoma and soil-transmitted helminthiases in children 1 to 9 years old in rural communities of the Amazon basin in Loreto Department, Peru: Results from a population-based survey. PLoS neglected tropical diseases. 2022 Jul 25;16(7):e0010532. | Excluded | Outcome of interest not reported and the study done outside Ethiopia |
| 541 | Kashaf MS, Muñoz BE, Mkocha H, Wolle MA, Naufal F, West SK. Incidence and progression of trachomatous scarring in a cohort of children in a formerly hyper-endemic district of Tanzania. PLoS neglected tropical diseases. 2020 Oct 5;14(10):e0008708. | Excluded | Outcome of interest not reported and the study done outside Ethiopia |
| 542 | Kann RS, Snyder JS, Woreta M, Zewudie K, Freeman MC, Delea MG. Quantifying Factors Associated with Personal Hygiene as Measured by the qPHAT Methodology: Andilaye Trial, Ethiopia. The American Journal of Tropical Medicine and Hygiene. 2023 May 1;108(6):1277. | Excluded | Outcome of interest not reported |
| 543 | Mahmud H, Landskroner E, Amza A, Aragie S, Godwin WW, de Hostos Barth A, O’Brien KS, Lietman TM, Oldenburg CE. Stopping azithromycin mass drug administration for trachoma: A systematic review. PLoS Neglected Tropical Diseases. 2021 Jul 8;15(7):e0009491. | Excluded | Duplicate |
| 544 | Schaal LF, Meneghim RL, Ferraz LC, Padovani CR, Victoria C, Schellini SA. Trachoma: an underdiagnosed disease revealed by a survey carried out at Jaú, São Paulo. BMC ophthalmology. 2024 Jan 29;24(1):43. | Excluded | Outcome of interest not reported and the study done outside Ethiopia |
| 545 | Wolle MA, West SK. Ocular Chlamydia trachomatis infection: elimination with mass drug administration. Expert review of anti-infective therapy. 2019 Mar 4;17(3):189-200. | Excluded | Outcome of interest not reported and non-observational design |
| 546 | Amza A, Kadri B, Nassirou B, Cotter SY, Stoller NE, West SK, Bailey RL, Porco TC, Keenan JD, Lietman TM, Oldenburg CE. Community-level association between clinical trachoma and ocular chlamydia infection after MASS azithromycin distribution in a mesoendemic region of Niger. Ophthalmic epidemiology. 2019 Jul 4;26(4):231-7. | Excluded | Outcome of interest not reported and the study done outside Ethiopia |
| 547 | Hu V, Caswell R, Last A, Burton M, Mabey D. Trachoma and Inclusion conjunctivitis. InHunter's tropical medicine and emerging infectious diseases 2020 Jan 1 (pp. 421-428). Elsevier. | Excluded | Outcome of interest not reported |
| 548 | Wangui J, Mwau M. INNOVATIVE RESEARCH AND KNOWLEDGE. | Excluded | Outcome of interest not reported |
| 549 | Sherief ST, Sitotaw MS, Girma A. Prevalence of traditional eye medicine and self-treatment in Gurage Zone, Rural Ethiopia. BMC Complementary Medicine and Therapies. 2024 Jul 4;24(1):255. | Excluded | Outcome of interest not reported |
| 550 | Hirpesa GM, Kaur G, Haaland ØA, Johansson KA. Surgery for trachomatous trichiasis. | Excluded | Outcome of interest not reported |
| 551 | Hassan R, Hassan R, Mohamed S, Saad FM, Mohamed HA. Trachoma in Sudan: Risk Factors and Clinical Stages in Patients From Two Eye Care Hospitals, 2015. Cureus. 2024 Dec 25;16(12). | Excluded | Outcome of interest not reported and the study done outside Ethiopia |
| 552 | Hassan RS, Aabdeen MA, Hassan RS, Mohamed SO, Saad FM, Mohamed HA. Trachoma in Sudan: case series from two eye care hospitals and a review of the literature. | Excluded | Outcome of interest not reported and the study done outside Ethiopia |
| 553 | Sitoe HM, Oswald WE, Zita F, Fall M, Momade T, Adams MW, Flueckiger RM, McPherson S, Eyob S, Doan T, Lietman TM. Ongoing transmission of trachoma in low prevalence districts in Mozambique: results from four cross-sectional enhanced impact surveys, 2022. Scientific reports. 2024 Oct 15;14(1):22842. | Excluded | Outcome of interest not reported and the study done outside Ethiopia |
| 554 | Bucumi V, Muhimpundu E, Issifou AA, Akweyu S, Burn N, Willems J, Niyongabo J, Elvis A, Koizan G, Harte A, Boyd S. Baseline, Impact and Surveillance Trachoma Prevalence Surveys in Burundi, 2018–2021. Ophthalmic Epidemiology. 2024 Nov 1;31(6):543-52. | Excluded | Outcome of interest not reported and the study done outside Ethiopia |
| 555 | Czerniewska A, Versteeg A, Shafi O, Dumessa G, Aga MA, Last A, Macleod D, Sarah V, Dodson S, Negussu N, Sori BK. Comparison of face washing and face wiping methods for trachoma control. | Excluded | Outcome of interest not reported |
| 556 | West S, Watitu T, Senyonjo L, Ngondi J, Jesudason T. Integrating antibody and infection-based testing into trachoma surveys. Community Eye Health. 2024 Oct 2;37(123):21. | Excluded | Outcome of interest not reported |
| 557 | Borlase A, Blumberg S, Callahan EK, Deiner MS, Nash SD, Porco TC, Solomon AW, Lietman TM, Prada JM, Hollingsworth TD. Modelling trachoma post-2020: opportunities for mitigating the impact of COVID-19 and accelerating progress towards elimination. Transactions of the Royal Society of Tropical Medicine and Hygiene. 2021 Mar;115(3):213-21. | Excluded | Outcome of interest not reported |
| 558 | Sullivan KM, Harding-Esch EM, Keil AP, Freeman MC, Batcho WE, Bio Issifou AA, Bucumi V, Bella AL, Epee E, Bobo Barkesa S, Seife Gebretsadik F. Exploring water, sanitation, and hygiene coverage targets for reaching and sustaining trachoma elimination: G-computation analysis. PLoS neglected tropical diseases. 2023 Feb 13;17(2):e0011103. | Excluded | Outcome of interest not reported |
| 559 | Selby R, Jeyam A, Tate A, Kebede F, Downs P. Potential mitigating role of ivermectin on the spread of Chlamydia trachomatis by Musca sorbens. PLOS Neglected Tropical Diseases. 2023 Oct 26;17(10):e0011662. | Excluded | Outcome of interest not reported |
| 560 | Adamu MD, Mohammed Jabo A, Orji P, Zhang Y, Isiyaku S, Olobio N, Muhammad N, Barem B, Willis R, Bakhtiari A, Jimenez C. Baseline prevalence of trachoma in 21 local government areas of Adamawa State, North East Nigeria. Ophthalmic Epidemiology. 2023 Nov 2;30(6):599-607. | Excluded | Outcome of interest not reported and the study done outside Ethiopia |
| 561 | Macleod CK, Butcher R, Javati S, Gwyn S, Jonduo M, Abdad MY, Roberts CH, Keys D, Koim SP, Ko R, Garap J. Trachoma, anti-pgp3 serology, and ocular chlamydia trachomatis infection in Papua New Guinea. Clinical Infectious Diseases. 2021 Feb 1;72(3):423-30. | Excluded | Outcome of interest not reported and the study done outside Ethiopia |
| 562 | Chikwanda M, Mubita P, Munyinda N, Mwale C, Mbanefo P, Banda TC. Research Paper An association between water, sanitation, and hygiene (WASH) and prevalence of trachoma in Monze district of Southern Province, Zambia. | Excluded | Outcome of interest not reported and the study done outside Ethiopia |
| 563 | Tedijanto C, Aragie S, Tadesse Z, Haile M, Zeru T, Nash SD, Wittberg DM, Gwyn S, Martin DL, Sturrock HJ, Lietman TM. Predicting future ocular Chlamydia trachomatis infection prevalence using serological, clinical, molecular, and geospatial data. medRxiv. 2021 Jul 22:2021-07. | Excluded | Outcome of interest not reported |
| 564 | Williams LB, Prakalapakorn SG, Ansari Z, Goldhardt R. Impact and trends in global ophthalmology. Current ophthalmology reports. 2020 Sep;8:136-43. | Excluded | Outcome of interest not reported |
| 565 | Solomon AW, Kello AB, Bangert M, West SK, Taylor HR, Tekeraoi R, Foster A. The simplified trachoma grading system, amended. Bulletin of the World Health Organization. 2020 Sep 3;98(10):698. | Excluded | Outcome of interest not reported |
| 566 | Wharton-Smith A, Rassi C, Batisso E, Ortu G, King R, Endriyas M, Counihan H, Hamade P, Getachew D. Gender-related factors affecting health seeking for neglected tropical diseases: findings from a qualitative study in Ethiopia. PLoS neglected tropical diseases. 2019 Dec 12;13(12):e0007840. | Excluded | Outcome of interest not reported |
| 567 | Rolfe RJ, Shaikh H, Tillekeratne LG. Mass drug administration of antibacterials: weighing the evidence regarding benefits and risks. Infectious Diseases of Poverty. 2022 Jun 30;11(1):77. | Excluded | Outcome of interest not reported and the study done outside Ethiopia |
| 568 | Negash KH, Gasa V. Academic barriers that prevent the inclusion of learners with visual impairment in Ethiopian mainstream schools. SAGE Open. 2022 Apr;12(2):21582440221089934. | Excluded | Outcome of interest not reported |
| 569 | Damtie D, Ambelu GC, Belay DB. The Trend of Neglected Tropical Diseases in Gonji Kollela District in Amhara Region, Northwest Ethiopia: A Retrospective Study (2013-2022). | Excluded | Outcome of interest not reported |
| 570 | Maciel AM, Almeida NM, Silva AC, Almeida PC. Factors associated with trachoma treatment and control treatment in schools of municipality of the Northeast Region, Brazil. Revista Brasileira de Epidemiologia. 2020 Feb 21;23:e200011. | Excluded | Outcome of interest not reported and the study done outside Ethiopia |
