# Supplementary material for: Active trachoma among children aged 1–9 years in Ethiopia: A meta-analysis from 2019 to 2024
Source: PLoS One. 2025 May 9;20(5):e0323601. doi: 10.1371/journal.pone.0323601 (PMC12063883; doi:10.1371/journal.pone.0323601)
Supplement: S4 File — (DOCX) [file pone.0323601.s004.docx]

| **Authors** | Representativeness of the sample | Sample size justified and satisfactory | Non-respondents | Ascertainment of the exposure (risk factor) | subjects in different outcome groups are comparable, based on the study design  or analysis. Confounding factors are controlled. | Assessment of the outcome | Statistical test: | **NOS score** |
| --- | --- | --- | --- | --- | --- | --- | --- | --- |
| Asmare Z. et al | * | * | * | * | * | ** | * | 8 |
| Altaseb et al | * | * | * | * | ** | ** | * | 9 |
| Genet et al | * | * | * | * | ** | ** | * | 9 |
| Melkie et al | * | * | * | * | ** | ** | * | 9 |
| Shimelash et al | * | * | * | * | ** | ** | * | 9 |
| Mekonnen et al | * |  | * | * | * | ** | * | 7 |
| Shafi et al | * | * |  | * | * | ** | * | 7 |
| Tuke et al | * | * | * | * | * | ** | * | 8 |
| Mengiste et al | * | * | * | * | * | ** | * | 8 |
| Mohamed et al | * | * | * | * | * | ** | * | 8 |
| Delelegn et al] | * |  | * | * | * | ** | * | 7 |
| Getachew et al | * | * | * | * | ** | ** | * | 9 |
| Alemayehu et al | * | * |  | * | * | ** | * | 7 |
| Senebete et al | * | * | * | * | * | ** | * | 8 |
| Yitayeh et al | * | * | * | * | * | ** | * | 8 |
| Abdilwohab et al | * | * | * | * | * | ** | * | 9 |
| Shemsu et al | * | * | * | * | * | ** | * | 8 |
