# Supplementary material for: Active trachoma among children aged 1–9 years in Ethiopia: A meta-analysis from 2019 to 2024
Source: PLoS One. 2025 May 9;20(5):e0323601. doi: 10.1371/journal.pone.0323601 (PMC12063883; doi:10.1371/journal.pone.0323601)
Supplement: Attachment — Submitted filename: PONE-D-24-40061.pdf [file pone.0323601.s005.pdf]

# PLOS ONE

# Active trachoma among children aged 1-9 years in Ethiopia: A meta-analysis from 2019 to 2024

--Manuscript Draft--

| | |
|---|---|
| **Manuscript Number:** | PONE-D-24-40061 |
| **Article Type:** | Research Article |
| **Full Title:** | Active trachoma among children aged 1-9 years in Ethiopia: A meta-analysis from 2019 to 2024 |
| **Short Title:** | Active trachoma among children in Ethiopia |
| **Corresponding Author:** | Kibruyisfaw Weldeab Abore, M.D<br>St Paul's Hospital Millennium Medical College<br>Addis Ababa, Ethiopia ETHIOPIA |
| **Keywords:** | Active Trachoma, Children, Ethiopia |
| **Abstract:** | Background: Trachoma is a leading infectious cause of blindness and of significant public health concern targeted for elimination. This study aimed to systematically summarize the magnitude of active trachoma among children aged 1-9 in Ethiopia from 2019-2024.Methods: The review was prospectively registered on PROSPERO (Registration number: CRD42024514026). Database searches were conducted on Google Scholar, SCOPUS, PubMed, EMBASE, and AJOL for studies published between January 2019 to 31-March-2024 and with restriction to articles published only in English. Data extraction was done using a pre-prepared Excel sheet. STATA version 17 was used to perform the analysis. Heterogeneity between studies was assessed using I2 statistics and Cochrane Q. Qualitative synthesis was done to summarize the studies and random effect model was used to estimate the Pooled magnitude of active trachoma with a corresponding 95% confidence interval.Results: A total of 17 studies with 19793 subjects were included in the meta-analysis. The pooled magnitude of active trachoma among children aged 1-9 years was found to be 18.4% (95% CI: 13.88, 22.91). We found a statistically significant heterogeneity between studies. Among the regions, Southwest region was found to have the highest magnitude (44.1%) (95%CI: 41.8%, 46.4%) and Dire Dawa was found to have the lowest (4.3%) (95%CI: 2.9%, 5.7%). Conclusion: The magnitude of active trachoma is still higher than the WHO target for elimination. There was significant interregional difference in magnitude of active trachoma. Strengthening SAFE strategy and health education and promotion is recommended. |
| **Order of Authors:** | Kibruyisfaw Weldeab Abore, M.D |
| | Melat Tesfaye Asebot, MD |
| | Gifty Berhanemeskel Kebede, MD |
| | Robel Tibebu Kasaye, MD |
| | Asonya Abera Akuma, MD |
| | Mahlet Minwuyelet Dagne, MD |
| | Tewobesta Fesseha Tesfaye, MD |
| | Mahlet Tesfaye Abebe, MD |
| | Estifanos Bekele Fole, MD |
| **Opposed Reviewers:** | |
| **Additional Information:** | |
| **Question** | **Response** |
| **Financial Disclosure**<br><br>Enter a financial disclosure statement that | The author(s) received no specific funding for this work. |

*Powered by Editorial Manager® and ProduXion Manager® from Aries Systems Corporation*

| | |
|---|---|
| describes the sources of funding for the work included in this submission. Review the submission guidelines for detailed requirements. View published research articles from *PLOS ONE* for specific examples.<br><br>This statement is required for submission and **will appear in the published article** if the submission is accepted. Please make sure it is accurate.<br><br>**Funded studies**<br>Enter a statement with the following details:<br>• Initials of the authors who received each award<br>• Grant numbers awarded to each author<br>• The full name of each funder<br>• URL of each funder website<br>• Did the sponsors or funders play any role in the study design, data collection and analysis, decision to publish, or preparation of the manuscript?<br><br>Did you receive funding for this work? | |
| **Competing Interests**<br><br>Use the instructions below to enter a competing interest statement for this submission. On behalf of all authors, disclose any competing interests that could be perceived to bias this work—acknowledging all financial support and any other relevant financial or non-financial competing interests.<br><br>This statement is required for submission and **will appear in the published article** if the submission is accepted. Please make sure it is accurate and that any funding sources listed in your Funding Information later in the submission form are also declared in your Financial Disclosure statement.<br><br>View published research articles from *PLOS ONE* for specific examples. | The authors have declared that no competing interests exist. |

*Powered by Editorial Manager® and ProduXion Manager® from Aries Systems Corporation*

| | |
|---|---|
| **NO authors have competing interests**<br><br>Enter: *The authors have declared that no competing interests exist.*<br><br>**Authors with competing interests**<br><br>Enter competing interest details beginning with this statement:<br><br>*I have read the journal's policy and the authors of this manuscript have the following competing interests: [insert competing interests here]*<br><br>* typeset | |
| **Ethics Statement**<br><br>Enter an ethics statement for this submission. This statement is required if the study involved:<br><br>• Human participants<br>• Human specimens or tissue<br>• Vertebrate animals or cephalopods<br>• Vertebrate embryos or tissues<br>• Field research<br><br>Write "N/A" if the submission does not require an ethics statement.<br><br>General guidance is provided below. Consult the submission guidelines for detailed instructions. **Make sure that all information entered here is included in the Methods section of the manuscript.** | N/A |

| | |
|---|---|
| **Format for specific study types**<br><br>**Human Subject Research (involving human participants and/or tissue)**<br>• Give the name of the institutional review board or ethics committee that approved the study<br>• Include the approval number and/or a statement indicating approval of this research<br>• Indicate the form of consent obtained (written/oral) or the reason that consent was not obtained (e.g. the data were analyzed anonymously)<br><br>**Animal Research (involving vertebrate animals, embryos or tissues)**<br>• Provide the name of the Institutional Animal Care and Use Committee (IACUC) or other relevant ethics board that reviewed the study protocol, and indicate whether they approved this research or granted a formal waiver of ethical approval<br>• Include an approval number if one was obtained<br>• If the study involved *non-human primates*, add *additional details* about animal welfare and steps taken to ameliorate suffering<br>• If anesthesia, euthanasia, or any kind of animal sacrifice is part of the study, include briefly which substances and/or methods were applied<br><br>**Field Research**<br><br>Include the following details if this study involves the collection of plant, animal, or other materials from a natural setting:<br>• Field permit number<br>• Name of the institution or relevant body that granted permission | |
| **Data Availability**<br><br>Authors are required to make all data underlying the findings described fully available, without restriction, and from the time of publication. PLOS allows rare exceptions to address legal and ethical concerns. See the PLOS Data Policy and FAQ for detailed information. | Yes - all data are fully available without restriction |

| | |
|---|---|
| A Data Availability Statement describing where the data can be found is required at submission. Your answers to this question constitute the Data Availability Statement and **will be published in the article**, if accepted.<br><br>**Important:** Stating 'data available on request from the author' is not sufficient. If your data are only available upon request, select 'No' for the first question and explain your exceptional situation in the text box.<br><br>Do the authors confirm that all data underlying the findings described in their manuscript are fully available without restriction? | |
| **Describe where the data may be found in full sentences. If you are copying our sample text, replace any instances of XXX with the appropriate details.**<br><br>• If the data are **held or will be held in a public repository**, include URLs, accession numbers or DOIs. If this information will only be available after acceptance, indicate this by ticking the box below. For example: *All XXX files are available from the XXX database (accession number(s) XXX, XXX.*).<br>• If the data are all contained **within the manuscript and/or Supporting Information files**, enter the following: *All relevant data are within the manuscript and its Supporting Information files.*<br>• If neither of these applies but you are able to provide **details of access elsewhere**, with or without limitations, please do so. For example:<br><br>*Data cannot be shared publicly because of [XXX]. Data are available from the XXX Institutional Data Access / Ethics Committee (contact via XXX) for researchers who meet the criteria for access to confidential data.*<br><br>*The data underlying the results presented in the study are available from (include the name of the third party* | All relevant data are within the manuscript and its Supporting Information files. |

| | |
|---|---|
| *and contact information or URL).*<br>• This text is appropriate if the data are owned by a third party and authors do not have permission to share the data.<br><br>* typeset | |
| Additional data availability information: | |

*Powered by Editorial Manager® and ProduXion Manager® from Aries Systems Corporation*

## Cover letter

Dear editor,

Trachoma is still a public health concern in Ethiopia and the relevance of this study is multifaceted. Firstly, it updates and expands upon previous research done in 2019 by including data from the past five years, a period marked by significant socio-political upheavals in Ethiopia. This context is crucial for understanding the current state of trachoma and the additional challenges faced in controlling the disease. The various conflicts and wars in place, which from the outset were high trachoma prevalence areas warrants the investigation.

Secondly, by highlighting the ongoing high prevalence and regional disparities, the study underscores the need for tailored, region-specific public health strategies. Finally, the study's findings can inform policymakers and health organizations about the critical areas needing intervention, thereby helping to allocate resources more effectively and design more impactful health programs.

All authors have reviewed and approved the manuscript for submission. This manuscript has not been published elsewhere and it is not under consideration in any journal. The authors declare no conflict of interest and we don't have any opposed peer reviewer.

Sincerely,

Kibruyisfaw Weldeab Abore (MD, MPH)

Manuscript

Click here to access/download;Manuscript;Manuscript editted.docx

# Active trachoma among children aged 1-9 years in Ethiopia: A meta-analysis from 2019 to 2024

Kibruyisfaw Weldeab Abore[1], Melat Tesfaye Asebot[2], Gifty Berhanemeskel Kebede[1], Robel Tibebu Kasaye[1], Asonya Abera Akuma[3], Mahlet Minwuyelet Dagne[4], Tewobesta Fesseha Tesfaye[5], Mahlet Tesfaye Abebe[6], Estifanos Bekele Fole[6]

[1]Department of Ophthalmology, St. Paul's Hospital Millennium Medical College, Addis Ababa, Ethiopia

[2]Bloomberg School of Public Health, John Hopkins University, USA

[3]College of medicine and health sciences, Arbaminch University, Arbaminch, Ethiopia

[4]St. Peter's specialized hospital, Addis Ababa, Ethiopia

[5]Ministry of health Ethiopia, Addis Ababa, Ethiopia

[6]Department of Medicine, Yirgalem Hospital Medical College, Yirgalem, Ethiopia

Corresponding author:

Kibruyisaw Weldeab Abore (Kibruyisfaww@gmail.com)

# Abstract

**Background**: Trachoma is a leading infectious cause of blindness and of significant public health concern targeted for elimination. This study aimed to systematically summarize the magnitude of active trachoma among children aged 1-9 in Ethiopia from 2019-2024.

**Methods:** The review was prospectively registered on PROSPERO (Registration number: CRD42024514026). Database searches were conducted on Google Scholar, SCOPUS, PubMed, EMBASE, and AJOL for studies published between January 2019 to 31-March-2024 and with restriction to articles published only in English. Data extraction was done using a pre-prepared Excel sheet. STATA version 17 was used to perform the analysis. Heterogeneity between studies was assessed using $I^2$ statistics and Cochrane Q. Qualitative synthesis was done to summarize the studies and random effect model was used to estimate the Pooled magnitude of active trachoma with a corresponding 95% confidence interval.

**Results:** A total of 17 studies with 19793 subjects were included in the meta-analysis. The pooled magnitude of active trachoma among children aged 1-9 years was found to be 18.4% (95% CI: 13.88, 22.91). We found a statistically significant heterogeneity between studies. Among the regions, Southwest region was found to have the highest magnitude (44.1%) (95%CI: 41.8%, 46.4%) and Dire Dawa was found to have the lowest (4.3%) (95%CI: 2.9%, 5.7%).

**Conclusion:** The magnitude of active trachoma is still higher than the WHO target for elimination. There was significant interregional difference in magnitude of active trachoma. Strengthening SAFE strategy and health education and promotion is recommended.

## Introduction

Trachoma is an infectious ocular disease caused by the bacteria Chlamydia trachomatis and it is the leading infectious cause of blindness worldwide [1]. Active trachoma is defined by the presence of trachomatous inflammation–follicular (TF) and/or trachomatous inflammation–intense (TI) in at least one eye [2]. Trachoma primarily affects impoverished communities within low-income and middle-income nations. Trachoma is endemic in 57 countries globally, and it is responsible for the blindness and visual impairment of nearly 2 million people [3-5].

Africa bears the largest burden of trachoma globally with prevalence of TF ≥30% at most recent surveys [6]. Trachoma is the second most common cause of blindness in Ethiopia and still constitutes a disease of public health concern [7].

Trachoma disproportionately affects disadvantaged and marginalized groups within communities [8]. In endemic areas, active cases of the disease are common among the preschool children, with varying magnitude [9]. Children exhibiting the active stages of trachoma also serves as the reservoir for the transmission of infection [10]. Trachoma is managed through an endorsed, comprehensive strategy comprising of surgical treatment for trichiasis, antibiotic therapy, facial hygiene, and environmental improvement, collectively known as the SAFE strategy [11].

The relevance of this study is multifaceted. Firstly, it updates and expands upon previous research by including data from the past five years, a period marked by significant socio-political upheavals in Ethiopia. A previous meta-analysis done in Ethiopia in 2019 had shown the magnitude of active trachoma among children to be 26.9% [8]. This magnitude is expected to rise due to the wars, conflicts, and its associated internal displacements in Ethiopia over the past 5 years. Furthermore,

public health efforts would be affected which would negatively affect the progress towards trachoma elimination [12]. In addition, multiple studies were carried out in various regions of Ethiopia over the last five years and significant discrepancies in magnitude were seen. This concern highlights the need to combine the findings from these studies to establish evidence-based suggestions. Thus, this study aimed to systematically summarize and pool the magnitude of active trachoma among children aged 1-9 years in Ethiopia from 2019-2024.

# Method and materials

## Study design

This systematic review and meta-analysis was prospectively registered on PROSPERO (Registration number: CRD42024514026).

## Search strategy and sources

Two reviewers independently conducted searches on Google Scholar, SCOPUS, PubMed, EMBASE, and AJOL. The search was made using the terms active trachoma, children, and Ethiopia using Boolean operators 'AND' and 'OR'. The search was restricted to articles published between January 2019 to March 31 2024 and to those published in English. Literatures were rechecked after completion of the study. Preferred Reporting Items for Systematic reviews and Meta-Analyses (PRISMA) guideline was used to report the study selection steps.

## Eligibility criteria

The inclusion criteria for the study was studies that were conducted between 2019-2024, that are published in English, and that assessed the magnitude of active trachoma among children 1-9 years

in Ethiopia. Studies that did not report on the outcome of interest, studies done before 2019, those conducted outside of Ethiopia, studies which are not published in English language, studies which are not peer reviewed, and studies without retrievable full article were excluded from the study.

## Quality appraisal

The Newcastle-Ottawa scale (NOS) adapted for cross-sectional studies was used to assess the quality of studies included in the review [13]. It assess selection, comparability, and outcome domains of studies with 5, 2, and 3 stars maximum given for selection, comparability, and outcome domains respectively. Two reviewers independently assessed the quality of studies. Studies with a score of less than 7 were labeled as poor and those with ≥7 were assessed as good quality studies. A third reviewer settled disagreement in scoring between the two reviewers through discussions.

## Data extraction

Identified literature were imported to Covidence and examined against the eligibility criteria by two reviewers. A third reviewer served as a mediator when there was disagreement. Using a pre-prepared excel sheet, two reviewers with the help of a third reviewer, extracted data. We collected data regarding the name of the primary author, publication year, year of study conduct, region of the study, study design, sample size, and active trachoma magnitude with 95% confidence interval.

## Data processing and analysis

The extracted data were exported to STATA v.17 for analysis. Cochran's Q test and $I^2$ statistics were used to assess heterogeneity among studies. Using a random effects model, we computed the

pooled magnitude of active trachoma among children and presented it using a forest plot. A Subgroup analysis was conducted based on region and Meta-regression was done using sample size and year of publication as covariates. A leave one out sensitivity analysis was computed. We assessed publication bias using Funnel plot and Eggers test.

## Ethical statement

N/A

# Results

## Study screening and selection

The search strategy identified a total of 570 studies. Subsequently, 98 duplicate studies were removed. After reviewing title and abstract, 422 studies were removed. Full article screening was done on 50 articles, and 9 studies that did not report on magnitude of trachoma, 21 studies based on year of study, 1 study done on adult subjects, and 2 studies without clear study period were excluded. Finally, 17 studies were found to be eligible for the meta-analysis. (**Fig 1**)

**Fig 1. PRISMA study selection flow for systematic review and meta-analysis of active trachoma among children aged 1-9 years in Ethiopia: A meta-analysis from 2019 to 2024**

## Study characteristics

Of the studies, 5 were done in Amhara region [14-18], 3 in Oromia region [19-21], 2 from Southen region [22, 23], 3 in Gambella [24-26], 1 from South West Ethiopia region[27], 1 in Benishangul

Gumuz [28], 1 in Sidama [29], and 1 in Diredawa city [30]. Based on NOS assessment of quality, all included studies had good quality (a score ≥7). In terms of the number of study participants, the largest sample size was 5489 [25] and smallest was 178 [20]. All included studies were cross-sectional study design. (**Table 1**)

**Table 1. Characteristics of studies included for systematic review and meta-analysis on active trachoma among children aged 1-9 years in Ethiopia from 2019-2024**

| Authors | Year of publication | Region | Study design | SS | NOS score |
|---|---|---|---|---|---|
| Asmare Z. et al[16] | 2023 | Amhara | Cross-sectional | 540 | 8 |
| Altaseb et al [15] | 2024 | Amhara | Cross-sectional | 616 | 9 |
| Genet et al [14] | 2022 | Amhara | Cross-sectional | 704 | 9 |
| Melkie et al[17] | 2020 | Amhara | Cross-sectional | 678 | 9 |
| Shimelash et al[18] | 2022 | Amhara | Cross-sectional | 394 | 9 |
| Mekonnen et al[20] | 2022 | Oromia | Cross-sectional | 178 | 7 |
| Shafi et al[21] | 2023 | Oromia | Cross-sectional | 526 | 7 |
| Tuke et al[19] | 2023 | Oromia | Cross-sectional | 538 | 8 |
| Mengiste et al[28] | 2022 | Benishangul | Cross-sectional | 4590 | 7 |
| Mohamed et al[30] | 2019 | Diredawa | Cross-sectional | 823 | 8 |
| Delelegn et al[29] | 2021 | Sidama | Cross-sectional | 701 | 7 |
| Getachew et al[27] | 2023 | South West Ethiopia | Cross-sectional | 1292 | 10 |
| Alemayehu et al[25] | 2023 | Gambella | Cross-sectional | 5489 | 7 |
| Senebete et al [24] | 2024 | Gambella | Cross-sectional | 722 | 8 |
| Yitayeh et al [26] | 2021 | Gambella | Cross-sectional | 610 | 8 |
| Abdilwohab et al[22] | 2020 | Southern region | Cross-sectional | 831 | 9 |

| | | | | | |
|---|---|---|---|---|---|
| Shemsu et al[23] | 2021 | Southern region | Cross-sectional | 561 | 8 |

## Meta-analysis of Active trachoma among children 1-9 years of age in Ethiopia

We included 17 studies for the meta-analysis with a total sample size of 19793. Due to the statistically significant heterogeneity among studies, we used random effect model to estimate the pooled magnitude of active trachoma. The magnitude of active trachoma among children was found to be 18.4% (95% CI: 13.88, 22.91) (**Fig 2**). Meta-regression done using sample size and year of study found that both are not statistically significant sources of heterogeneity (**Table 2**). Subgroup analysis based on the region of the study was conducted and between studies heterogeneity persisted. Southwest region was found to have the highest magnitude of active trachoma (44.1%) while Dire Dawa City was found to have the lowest magnitude (4.3%).(**Table 3**) .

**Fig 2. Pooled magnitude of active trachoma among children aged 1-9 years in Ethiopia from 2019-2024**

**Table 2. Meta-regression analysis of factors affecting between-study heterogeneity.**

| Source | Coefficient | Std. error | p-value |
|---|---|---|---|
| Year of publication | 2.81268 | 1.87961 | 0.135 |
| Sample size | -0.02064 | 0.00180 | 0.253 |

**Table 3. Subgroup analysis based on region for active trachoma among children aged 1-9 years in Ethiopia from 2019-2024**

| Region | Pooled magnitude | 95% CI | Heterogeneity ($I^2$) |
|---|---|---|---|
| Amhara | 14% | 3.57, 24.44 | 98.87% |
| Oromia | 24.11% | 18.71, 29.5 | 76.59% |
| Southern region | 23.51% | 12.14, 34.88 | 96.13% |
| Sidama | 17.5% | 14.15, 20.85 | - |
| Gambella | 16.82% | 11.68, 21.96 | 91.43% |
| Southwest region | 44.1% | 41.8, 46.4 | - |
| Dire Dawa | 4.3% | 2.9, 5.7 | - |
| Benishangul Gumuz | 8.04% | 7.24, 8.83 | - |

# Sensitivity analysis

A leave-one-out sensitivity analysis was conducted to assess the effect of one study on the overall effect size estimate. We found that no single study had a significant effect on the overall effect size estimate. (**Table 4**)

**Table 4. A leave one out sensitivity analysis for studies on active trachoma among children aged 1-9 years in Ethiopia from 2019-2024**

| Omitted study | Effect size | [95% conf. interval] | | p-value |
|---|---|---|---|---|
| | | Upper | lower | |
| Asmare ZA, 2023 | 17.348 | 12.873 | 21.824 | 0.000 |
| Altaseb T., 2024 | 18.875 | 14.118 | 23.632 | 0.000 |
| Genet A., 2022 | 19.192 | 14.418 | 23.967 | 0.000 |
| Melkie G, 2020 | 19.043 | 14.269 | 23.817 | 0.000 |
| Shimelash A., 2022 | 18.933 | 14.207 | 23.660 | 0.000 |
| Mengistu B., 2022 | 19.084 | 13.824 | 24.343 | 0.000 |
| Mohamed H., 2019 | 19.303 | 14.556 | 24.051 | 0.000 |
| Alemayehu A., 2023 | 18.781 | 13.338 | 24.224 | 0.000 |
| Senbete L, 2024 | 18.515 | 13.830 | 23.199 | 0.000 |
| Yitayeh B, 2021 | 18.197 | 13.536 | 22.857 | 0.000 |
| Mekonnen J, 2022 | 18.195 | 13.553 | 22.838 | 0.000 |
| Shafi Abdurahman O, 2023 | 18.241 | 13.577 | 22.905 | 0.000 |
| Tuke D., 2023 | 17.732 | 13.154 | 22.309 | 0.000 |
| Delelegn D., 2021 | 18.454 | 13.760 | 23.148 | 0.000 |
| Getachew D., 2023 | 16.623 | 13.369 | 19.877 | 0.000 |
| Abdilwohab G., 2020 | 18.438 | 13.712 | 23.163 | 0.000 |
| Shemsu K, 2021 | 17.712 | 13.153 | 22.271 | 0.000 |
| Theta | 18.396 | 13.884 | 22.908 | 0.000 |

## Publication bias

We assessed publication bias using a funnel plot and Egger's test. The funnel plot was found to be slightly asymmetric to the right (**Fig 3**) and Egger's test showed a statistically significant publication bias (p-value=0.0112). Subsequently, trim and fill analysis was done with no change in the estimated effect size. The result of Egger's test was assumed to be originating from the statistically significant heterogeneity.

**Fig 3. Funnel plot assessing publication bias for active trachoma among children aged 1-9 years in Ethiopia from 2019-2024**

# Discussion

The study showed that there is a high prevalence of active trachoma among children 1-9 years in Ethiopia, with significant variation in prevalence among different regions of the country. The pooled variance is 18.4% which is markedly higher than the World Health Organization's (WHO) target of trachoma elimination of less than 5% active trachoma [4]. The finding is comparable with a previous report by Gebrie et al in 2019 which showed a pooled prevalence of 26.9% and similar disparities among regions. [8] Although their study reported a similar pattern and trend, the study period was limited to the year 2019. Our study extended from 2019-2024 which makes it relevant in capturing effects of recent occurrences such as the ongoing war and conflicts in the country over the past five years.

Previous studies have highlighted the significant consequence of war and displacement in hindering disease control by disrupting healthcare infrastructure, reducing access to clean water and sanitation, and spreading infectious diseases because of migration [5, 12]. Despite the current

ongoing war and displacement in the country, the pooled prevalence of our study is lower than the one reported 5 years ago (26.9% Vs 18.4%). One possible reason could be that data from Tigray and Afar, regions which are heavily affected by the war, are not included in our study due to a lack of data from those regions. Therefore, the reported prevalence might underestimate the true prevalence.

Several other factors may contribute to this high prevalence rate that persisted in the country despite implementing control measures to reduce the burden. The low socioeconomic status of the country is the major one which results in a lack of access to clean water and inadequate sanitation. According to UNICEF and WHO's report, 48% of the population lacks access to clean water and functional latrines, which significantly impacts hygiene practices and increases the risk of trachoma transmission [31]. Lack of hygiene is a significant factor in the spread of infection. Global studies have indicated that countries with poor sanitation and water supply have higher trachoma prevalence, underscoring the need for comprehensive public health interventions [32-34]. A study by Garn et al. demonstrated the inverse relation between adequate water and sanitation facilities and trachoma prevalence [2]. Poverty is also inversely related to education and health care access which are essential factors to the successful implementation of control measures. Trachoma was indicated as an indicator of poverty [35].

Cultural practices are another factor contributing to the high prevalence of trachoma. Some practices such as using clothes to clean eyes, inadequate showers, and facial cleanliness further been reported to increase the risk of trachoma transmission [36]. War and conflict on top of this lays a fertile ground for the increase in transmission.

To reduce Trachoma prevalence the WHO recommended SAFE (Surgery for trichiasis, Antibiotic to clear infection, Facial cleanliness, and Environmental improvement) strategy should be

strengthened [6, 37]. Building infrastructures for Water, Sanitation, and Hygiene (WASH) is also critical to hinder the transmission of trachoma [3, 6]. Special emphasis should be given to areas with higher prevalence. Empowering the public by raising awareness about the severity of the prevalence and the resulting blindness as well as the hygiene techniques could ease the burden and should be widely implemented. A due focus also should be given to expanding and equipping healthcare facilities with trained staff and resources to combat trachoma. Lastly, a robust surveillance system to monitor the prevalence and impact of intervention should be in play to monitor and evaluate the implementations.

Eleven countries, including 3 from Africa (The Gambia, Ghana, Morocco), have eliminated trachoma in 2022 [1]. This could indicate public health interventions such as the sustained SAFE strategy implementation can lead to the elimination of trachoma. These countries serve as a great example and those strategies should be tailored to the country's status and implemented to achieve the elimination target.

# Limitation

Despite its strength in shedding light on the prevalence of trachoma, our study has some limitations. The first is that the included studies are cross-sectional studies hence limiting causal inferences. Second, the exclusion of conflict-affected regions like Tigray and Afar due to a lack of recent data may result in an underestimation of the true prevalence of active trachoma in Ethiopia. This warrants cautious interpretation of the result.

# Conclusion

Although it has decreased from the previously reported magnitude, active trachoma is still higher than the WHO target for elimination and is a disease of public health concern. We recommend

strengthening the SAFE strategy to better tackle the problem as well as working with relevant stakeholders about health education and health promotion. Future research should focus on longitudinal analysis to better understand the transmission of trachoma and associated socio-demographic factors in different regions of the country. The long-term impact of the ongoing intervention should also be assessed in future studies. These studies will help tailor public health interventions based on the specific findings in the regions. Additionally, research comparing trachoma control efforts in Ethiopia with those in other high-prevalence countries can provide valuable insights into effective strategies and potential areas for improvement.

# References


1. Renneker KK, Abdala M, Addy J, Al-Khatib T, Amer K, Badiane MD, et al. Global progress toward the elimination of active trachoma: an analysis of 38 countries. The Lancet Global Health. 2022;10(4):e491-e500.

2. Garn JV, Boisson S, Willis R, Bakhtiari A, Al-Khatib T, Amer K, et al. Sanitation and water supply coverage thresholds associated with active trachoma: modeling cross-sectional data from 13 countries. PLoS neglected tropical diseases. 2018;12(1):e0006110.

3. Dye C, Mertens T, Hirnschall G, Mpanju-Shumbusho W, Newman RD, Raviglione MC, et al. WHO and the future of disease control programmes. Lancet. 2013;381(9864):413-8.

4. Organization WH. Global WHO alliance for the elimination of blinding trachoma by 2020. Weekly Epidemiological Record= Relevé épidémiologique hebdomadaire. 2012;87(17):161-8.

5. Marie. Deadly comrades: war and infectious disease. THE LANCET. 2002;360.

6. Report of the 21th meeting of the WHO alliance for the global elimination of trachoma by 2020 [Internet]. Available from: https://www.who.int/publications/i/item/who-cds-ntd-pct-2019.02 Report of the 21th meeting of the WHO alliance for the global elimination of trachoma by 2020.

7. Miecha H, Dejene M, Adugna D, Kebede A, Yadeta D, Alemayehu A, et al. Prevalence of Trachoma after Implementation of Trachoma Elimination Interventions in Oromia Regional State, Ethiopia: Results of Impact Surveys in 131 Evaluation Units Covering 139 Districts. Ophthalmic Epidemiology. 2023;30(6):647-54.

8. Gebrie A, Alebel A, Zegeye A, Tesfaye B, Wagnew F. Prevalence and associated factors of active trachoma among children in Ethiopia: a systematic review and meta-analysis. BMC Infectious Diseases. 2019;19(1):1073.

9. Ferede AT, Dadi AF, Tariku A, Adane AA. Prevalence and determinants of active trachoma among preschool-aged children in Dembia District, Northwest Ethiopia. Infectious Diseases of Poverty. 2017;6(1):128.

10. Australia CDN. CDNA National Guidelines for the Public Health Management of Trachoma. Do Health, Editor. 2014.

11. Tian L, Wang NL. Trachoma control: the SAFE strategy. Int J Ophthalmol. 2018;11(12):1887-8.

12. Marou V, Vardavas CI, Aslanoglou K, Nikitara K, Plyta Z, Leonardi-Bee J, et al. The impact of conflict on infectious disease: a systematic literature review. Conflict and Health. 2024;18(1):27.

13. Modesti PA, Reboldi G, Cappuccio FP, Agyemang C, Remuzzi G, Rapi S, et al. Panethnic differences in blood pressure in Europe: a systematic review and meta-analysis. PloS one. 2016;11(1):e0147601.

14. Genet A, Dagnew Z, Melkie G, Keleb A, Motbainor A, Mebrat A, et al. Prevalence of active trachoma and its associated factors among 1–9 years of age children from model and non-model kebeles in Dangila district, northwest Ethiopia. Plos one. 2022;17(6):e0268441.

15. Altaseb T, Lingerew M, Adane M. Prevalence of trachomatous inflammation-follicular and associated factors among children aged 1-9 years in northeastern Ethiopia. BMC pediatrics. 2024;24(1):128.

16. Asmare ZA, Seifu BL, Mare KU, Asgedom YS, Kase BF, Shibeshi AH, et al. Prevalence and associated factors of active trachoma among 1–9 years of age children in Andabet district, northwest Ethiopia, 2023: A multi-level mixed-effect analysis. PLOS Neglected Tropical Diseases. 2023;17(8):e0011573.

17. Melkie G, Azage M, Gedamu G. Prevalence and associated factors of active trachoma among children aged 1-9 years old in mass drug administration graduated and non-graduated districts in Northwest Amhara region, Ethiopia: A comparative cross-sectional study. PLoS One. 2020;15(12):e0243863.

18. Shimelash A, Alemayehu M, Dagne H, Mihiretie G, Lamore Y, Tegegne E, et al. Prevalence of active trachoma and associated factors among school age children in Debre Tabor Town, Northwest Ethiopia, 2019: a community based cross-sectional study. Italian Journal of Pediatrics. 2022;48(1):61.

19. Tuke D, Etu E, Shalemo E. Active trachoma prevalence and related variables among children in a pastoralist community in southern Ethiopia in 2021: a community-based cross-sectional study. The American Journal of Tropical Medicine and Hygiene. 2023;108(2):252.

20. Mekonnen J, Kassim J, Ahmed M, Gebeyehu N. Prevalence of active trachoma and associated factors among children 1–9 years old at Arsi Negele Town, West Arsi Zone, Oromia Regional State, Southern Ethiopia. Plos one. 2022;17(10):e0273808.

21. Shafi Abdurahman O, Last A, Macleod D, Habtamu E, Versteeg B, Dumessa G, et al. Trachoma risk factors in Oromia Region, Ethiopia. PLoS Neglected Tropical Diseases. 2023;17(11):e0011679.

22. Glagn Abdilwohab M, Hailemariam Abebo Z. High prevalence of clinically active trachoma and its associated risk factors among preschool-aged children in arba Minch health and demographic surveillance site, southern Ethiopia. Clinical Ophthalmology. 2020:3709-18.

23. Kedir S, Lemnuro K, Yesse M, Abdella B, Muze M, Mustefa A, et al. Prevalence and Factors Associated with Active Trachoma among Children 1-9 years of Age in the Catchment Population of Tora Primary Hospital, Silte zone, Southern Ethiopia, 2020. The Open Ophthalmology Journal. 2021;15(1).

24. Senbete L, Adhena G. Magnitude of Active Trachoma Among Host and Refugee Children in Gambella Regional State, Ethiopia. Clinical Ophthalmology. 2024:777-89.

25. Alemayehu A, Mekonen A, Mengistu B, Mihret A, Asmare A, Bakhtiari A, et al. Prevalence of Trachoma After Three Rounds of Antibiotic Mass Drug Administration in 13 Woredas of Gambella Region, Ethiopia. Ophthalmic epidemiology. 2023:1-9.

26. Belsti Y, Fekadu SA, Assem AS. Active trachoma prevalence and its associated factors among children aged 1-9 years in rural residents of Lare District, Southwest Ethiopia. International Journal of Ophthalmology. 2021;14(11):1756.

27. Getachew D, Woldekidan F, Ayele G, Bekele Y, Sleshi S, Tekalgn E, et al. High prevalence of active trachoma and associated factors among school-aged children in Southwest Ethiopia. PLOS Neglected Tropical Diseases. 2023;17(12):e0011846.

28. Mengistu B, Wirtu F, Alemayehu A, Alene S, Asmare A, Backers S, et al. Prevalence of trachoma in Benishangul Gumuz Region, Ethiopia, after implementation of the SAFE strategy: Results of four population-based surveys. Ophthalmic epidemiology. 2022:1-9.

29. Delelegn D, Tolcha A, Beyene H, Tsegaye B. Status of active trachoma infection among school children who live in villages of open field defecation: a comparative cross-sectional study. BMC public health. 2021;21:1-10.

30. Mohamed H, Weldegebreal F, Mohammed J, Gemechu A. Trachoma and Associated Factors among School Age Children 4-9 Years in Dire Dawa Administration, Eastern Ethiopia. East African Journal of Health and Biomedical Sciences. 2019;3(2):45-54.

31. Organization WH. 2017 annual report WHO/UNICEF joint monitoring programme for water supply, sanitation and hygiene. 2017 annual report WHO/UNICEF joint monitoring programme for water supply, sanitation and hygiene2017. p. 20-.

32. Satasuk Joy Bhosai RLB, Bruce D. Gaynor, Thomas M. Lietman. Trachoma: An Update on Prevention, Diagnosis, and Treatment. Curr Opin Ophthalmol. 2012.

33. Patel. BAMZBC. Trachoma. National Institutes of Health. 2024.

34. Yitayeh Belsti SAF, Abel Sinshaw Assem. Active trachoma prevalence and its associated factors among children aged 1-9 years in rural residents of Lare District, Southwest Ethiopia. Int J Ophthalmol. 2021;14.

35. Esmael Habtamu TW, Sintayehu Aweke, Zerihun Tadesse,, Mulat Zerihun ZZ, Kelly Callahan, Paul M. Emerson, et al. Trachoma and Relative Poverty: A Case- Control Study. PLOS Neglected Tropical Diseases. 2015.

36. Marx R. Social factors and trachoma: a review of the literature. Soc Sci Med. 1989;29(1):23-34.

37. Organization WH. Trachoma 2019 [Available from: https://www.who.int/news-room/fact-sheets/detail/trachoma.

# Supporting information

**S1. PRISMA 2020 checklist**

**S2. Data set**

Figure

Click here to access/download;Figure;Fig 1.tif

**PRISMA 2020 flow diagram for new systematic reviews which included searches of databases and registers only**

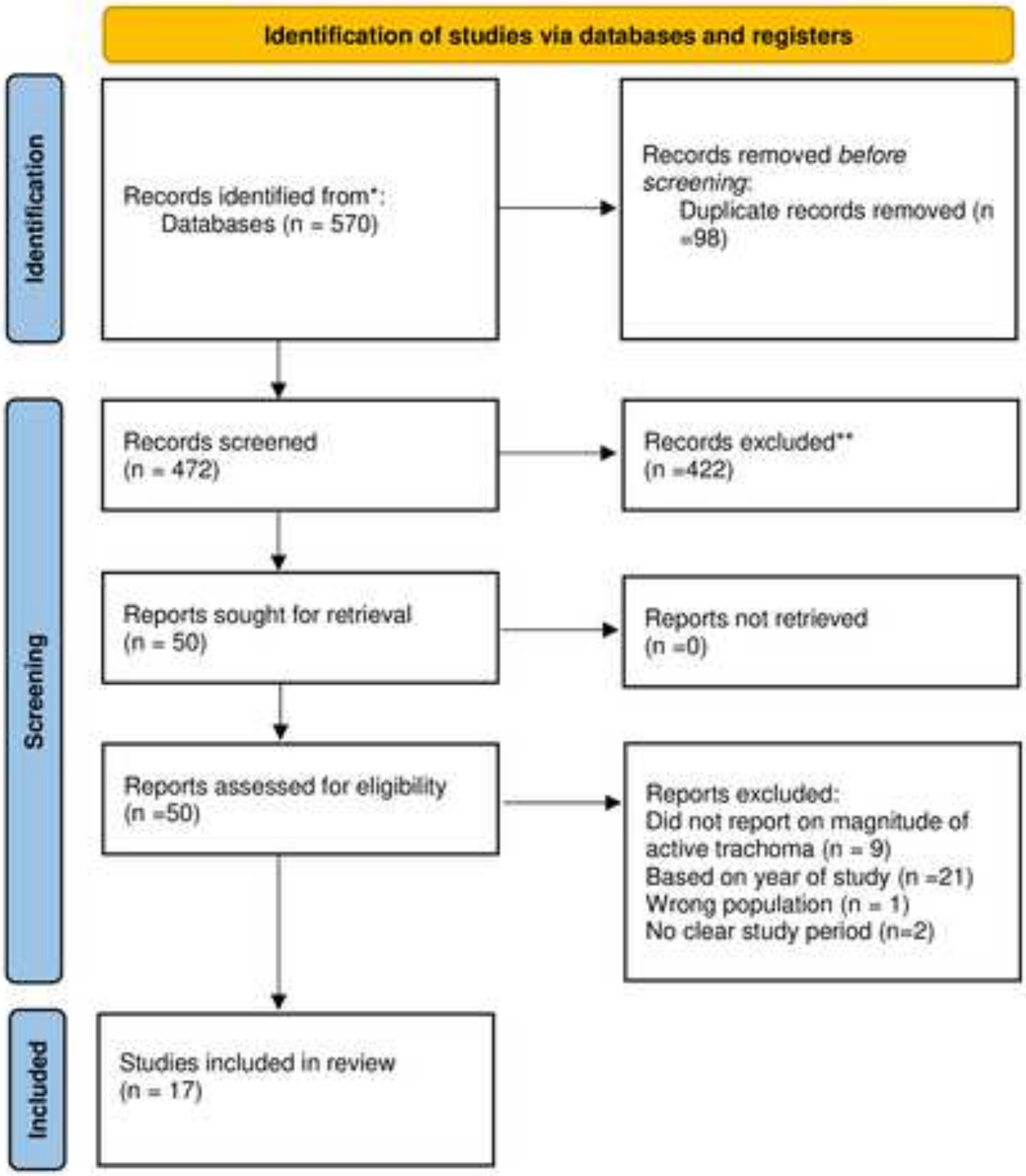


*Consider, if feasible to do so, reporting the number of records identified from each database or register searched (rather than the total number across all databases/registers).

**If automation tools were used, indicate how many records were excluded by a human and how many were excluded by automation tools.

*From:* Page MJ, McKenzie JE, Bossuyt PM, Boutron I, Hoffmann TC, Mulrow CD, et al. The PRISMA 2020 statement: an updated guideline for reporting systematic reviews. BMJ 2021;372:n71. doi: 10.1136/bmj.n71

Figure

Click here to access/download;Figure;Fig 2.tif

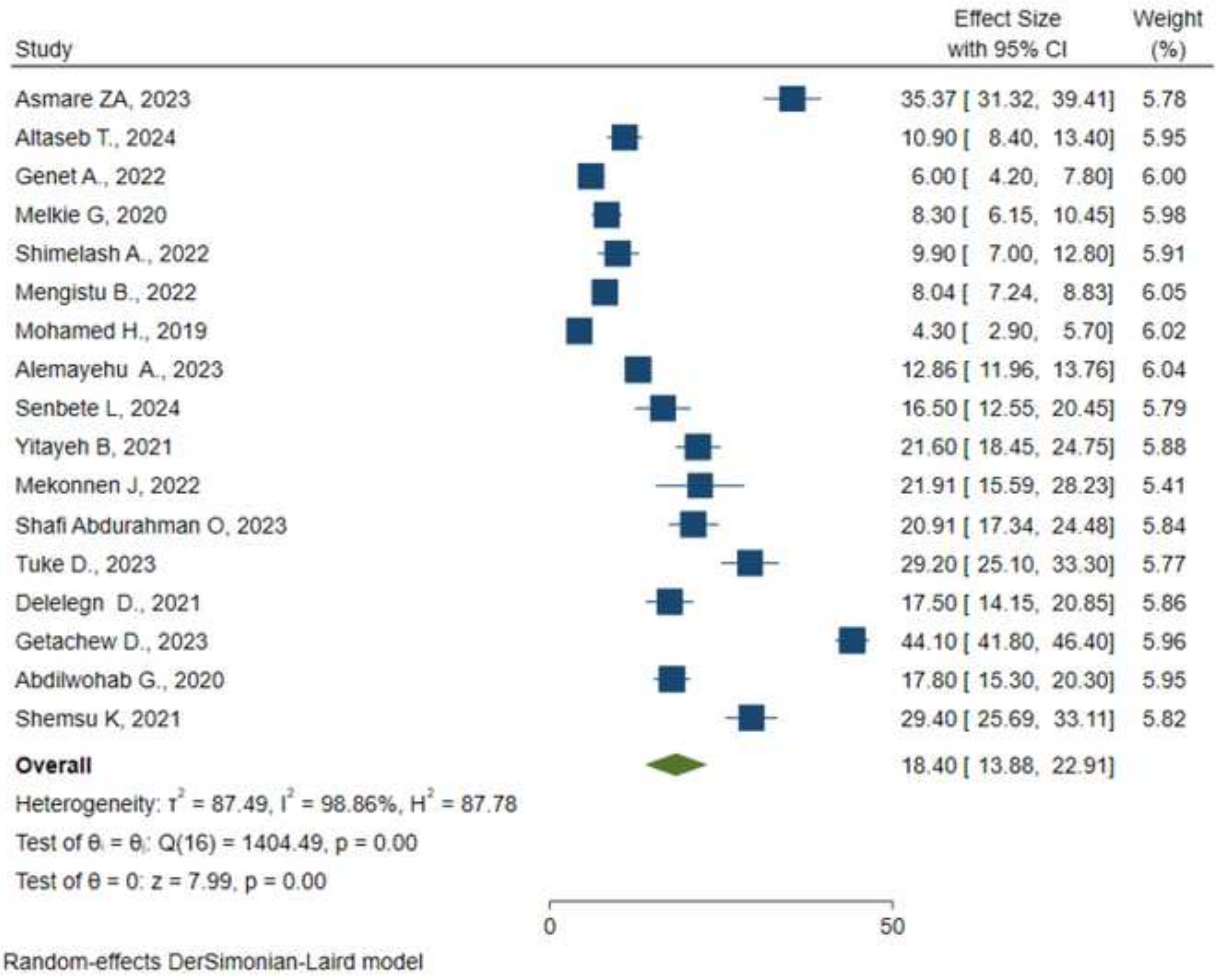

Figure

Click here to access/download;Figure;Fig 3.tif

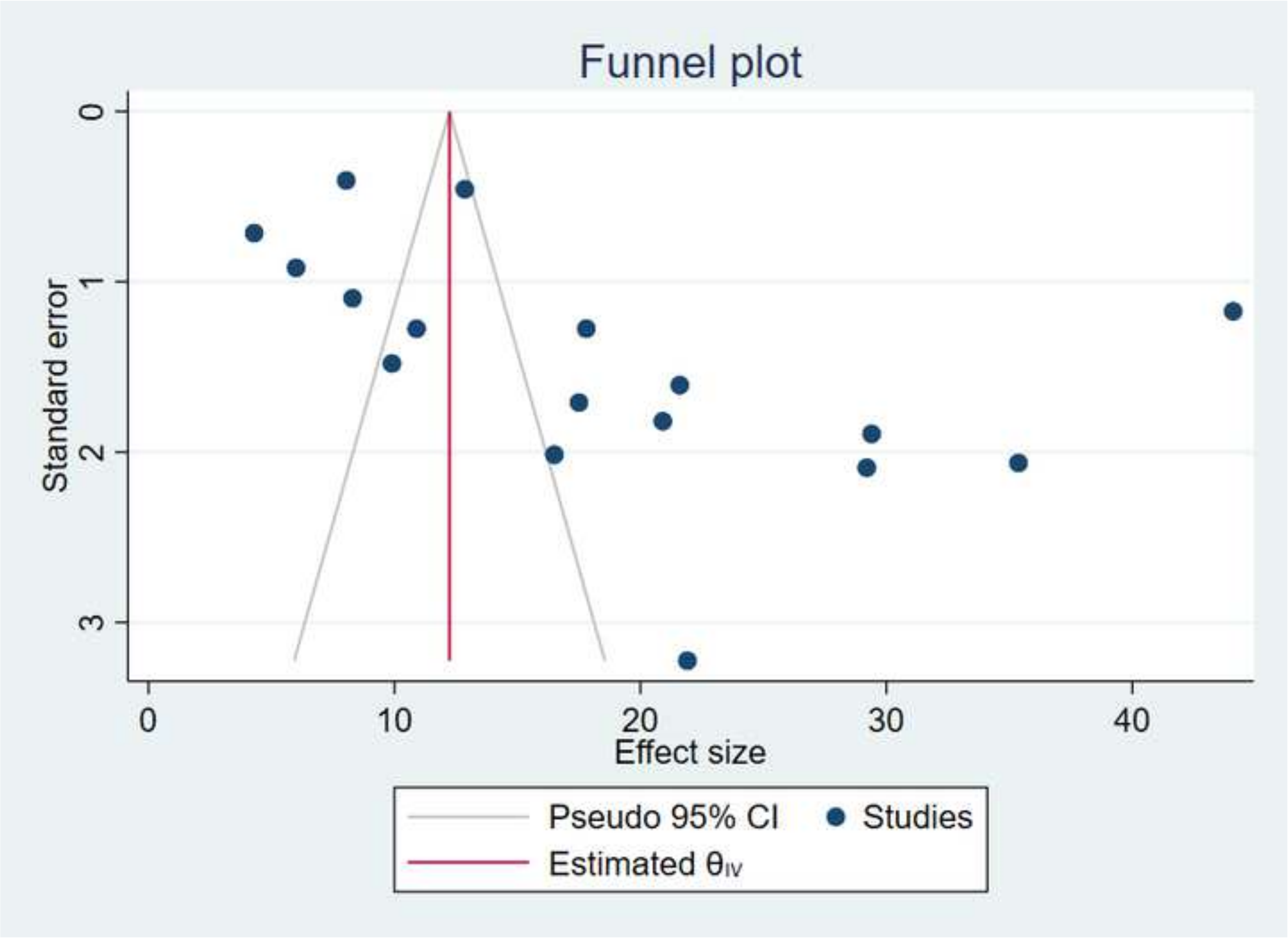

Click here to access/download
**Supporting Information**
Data set.xlsx

Click here to access/download
**Supporting Information**
PRISMA_2020_checklist.docx
